# Individual-level personality influences social foraging and collective behaviour in wild birds

**DOI:** 10.1098/rspb.2014.1016

**Published:** 2014-08-22

**Authors:** Lucy M. Aplin, Damien R. Farine, Richard P. Mann, Ben C. Sheldon

**Affiliations:** 1 Edward Grey Institute of Field Ornithology, University of Oxford, Oxford OX1 3PS, UK; 2 Research School of Biology, Australian National University, Acton, 2600 Australian Capital Territory, Australia; 3 Department of Anthropology, University of California, Davis, CA 95616, USA; 4 Smithsonian Tropical Research Institute, Ancon, Panama; 5 Department of Mathematics, Uppsala University, Box 480, 75106 Uppsala, Sweden

**Keywords:** collective decision-making, leader–follower, social information, group foraging, behavioural type, *Parus major*

## Abstract

There is increasing evidence that animal groups can maintain coordinated behaviour and make collective decisions based on simple interaction rules. Effective collective action may be further facilitated by individual variation within groups, particularly through leader–follower polymorphisms. Recent studies have suggested that individual-level personality traits influence the degree to which individuals use social information, are attracted to conspecifics, or act as leaders/followers. However, evidence is equivocal and largely limited to laboratory studies. We use an automated data-collection system to conduct an experiment testing the relationship between personality and collective decision-making in the wild. First, we report that foraging flocks of great tits (*Parus major*) show strikingly synchronous behaviour. A predictive model of collective decision-making replicates patterns well, suggesting simple interaction rules are sufficient to explain the observed social behaviour. Second, within groups, individuals with more reactive personalities behave more collectively, moving to within-flock areas of higher density. By contrast, proactive individuals tend to move to and feed at spatial periphery of flocks. Finally, comparing alternative simulations of flocking with empirical data, we demonstrate that variation in personality promotes within-patch movement while maintaining group cohesion. Our results illustrate the importance of incorporating individual variability in models of social behaviour.

## 1. Introduction

In many social species, complex and striking collective behaviour can arise from simple interaction rules [1]. Within-group responsiveness between neighbours allows for cohesion and consensus to form in movement decisions [2], predator avoidance [3] and resource exploitation [4], and thus provides important benefits for individual group members [5,6]. Although less well-studied, variation in the properties of individuals who comprise groups may be an important component of collective behaviour, with both phenotypic differences and within-group variation in social affiliations affecting decision-making processes [7–9]. In particular, if animals differ in their degree of sociality, then variation in the strength of social cohesion may mediate group-level movement, with asocial animals exerting directional ‘pulling power’ on more social individuals [10]. Such emergent leader (initiator)–follower polymorphisms [11] have been observed in the grouping behaviour of a diverse range of taxa, and leadership tendencies are increasingly proving to be consistent and repeatable within individuals [12,13]. While empirical support is limited, it is possible that variation in social behaviour within groups may also be important in balancing coordinated action with exploration, information-gathering and efficient patch exploitation [14–17].

Recent theoretical work has proposed that consistent grouping and leadership tendencies may relate to variation in individual-level personality traits [15,18]. If so, then such consistent and potentially heritable differences in personality may help characterize individuals’ roles in collective decision-making. Frequency-dependent selection on leader or follower behaviour may also help explain the persistence of variable personalities in natural populations [15,19], which to date remains one of the most contentious issues in animal personality research [20–22]. However, empirical support for a link between social behaviour and personality remains scarce, and evidence for the strength and direction of this relationship is equivocal. Most notably, sheep scored as shy in an assay for boldness were found to have a higher social attraction parameter [7] and graze closer to others [23], whereas in captive geese, bold individuals were more likely to make asocial decisions and less likely to use social information [24], though this effect was dependent on group size [25]. The evidence is clearer for studies of leadership in pairs of foraging fishes, where bold individuals were more likely to initiate movements from cover [12,26], move to new patches [27] and be less responsive to other individuals [26], tempered by effects of experience and motivation [13,28]. At the population-level, a social network approach in great tits has recently been used to demonstrate an association between social foraging and personality, with more proactive individuals more likely to move between flocks and holding shorter-term associations [29].

Within-group diversity in personality may also be an important component of efficient group movement, exploration and foraging success. In ants and social spiders, recent studies have suggested that mixed personality colonies are more successful, an effect thought to be linked to more efficient task allocation [30–32]. These group-level outcomes may further be influenced by the most extreme behavioural types present; in social spiders, the most proactive individual is the best predictor of group behaviour [33], whereas in guppies, group exploration is related to the behaviour of the least exploratory individual [17]. However, previous studies have largely been restricted to captive animals, and focused on discrete group outcomes (e.g. reproductive success [32,34]) rather than on collective movement or decision-making. We further have little knowledge of how personality may influence intragroup social interactions in the wild, where environments are dynamic and groups are comprised naturally associating individuals.

Here, we use an automated data-collection system to study flocking behaviour in a winter population of wild great tits (*Parus major*) and test the relationship between individual variation, social attraction and collective decision-making. Studies of the great tit represent one the most comprehensive examinations of the functional importance of personality to date, with personality quantified using the reactive–proactive axis common across multiple taxa. In comparison with reactive individuals, proactive individuals are more aggressive, exhibit fast and superficial exploration of novel environments, lower neophobia and increased boldness [35]. The axis is believed to reflect a trade-off between risk-taking and productivity, with individuals either prioritizing risk-prone behaviour with potentially high rewards, or more risk-averse behaviour that enhances survival [20]. As a proxy for this axis, we used an assay of exploration behaviour in a novel environment, scored on great tits temporarily taken into captivity. Exploration behaviour is repeatable [36], heritable [36], under selection [37,38] and linked to a range of life-history traits in several populations [35,38,39].

In our study, wild flocks of great tits were given a choice of four foraging locations within artificial habitat patches. Individuals were fitted with passive integrated transponder (PIT) tags, and foraging locations were fitted with radiofrequency identification (RFID) PIT-tag reading antennae. Great tits were identified in replicate habitat patches over two winters, and Bayes' rule used to calculate the probability of birds arriving at feeders as a function of the distribution of individuals across the patch. Social attraction towards the flock centre or periphery was then inferred by fitting a decision-making model of collective behaviour for each individual [40]. Collective behaviour was compared with two individual traits identified in King *et al.* [14] as likely to affect grouping and leadership tendencies: personality and dominance rank. Finally, we conducted simulations to gain an understanding of the extent to which consistent individual variation in the behaviour of group members influenced flock dynamics and patch exploitation, and compared the results of the simulations with our empirical data. We thus present complementary lines of evidences derived from experimental and computational modelling approaches to understand how individual-level personality traits influence collective decision-making behaviour.

## 2. Material and methods

### (a) Study area and population

The research was undertaken in a 385 ha area of broadleaf deciduous woodland at Wytham Woods, Oxfordshire (51°46′ N, 1°20′ W). The population of great tits in Wytham Woods has been the focus of an extensive long-term breeding survey, whereby all nestlings and adults are caught and fitted with British Trust for Ornithology metal leg-bands. Since 2007, all captured individuals have also been fitted with a uniquely coded PIT tag (IB Technology, Aylesbury), allowing automated detection of individuals with PIT-tag reading antennae. Our research was conducted in winter, when great tits form loose fission–fusion flocks that roam widely in search of ephemeral food sources. Typically, 60% of this wintering population of Wytham great tits is comprised locally born birds ringed and PIT-tagged as nestlings. The remaining immigrant birds were either caught as breeding adults in the breeding seasons previous to the study, or by using mist-nets at multi-access feeders during the autumn. Localized mist-netting was also conducted prior to field experiments, and we estimate that the proportion of PIT-tagged birds in the population exceeded 90% at the time of the study (electronic supplementary material, S1) [29].

### (b) Field methods

Over the winters of 2010/2011 and 2011/2012, we installed 20 replicated habitat patches (five and 15 in each year, respectively), spread throughout the woods. Each habitat patch comprised four identical sunflower feeders fitted with two RFID PIT-tag reading antennae (Francis Instruments, Cambridge, see Quinn *et al.* [41], Aplin *et al.* [29,42] for further details), and placed at the corners of a 50 × 50 m square. We aimed to position feeders such that individuals formed distinct subgroups at each feeder, but remained part of one overarching flock. Feeders were therefore spaced 50 m apart, as this is still within auditory and visual range of all the other feeders, but minimizes potential for individuals to feed on different feeders from the same perching location. With this in mind, feeders were also occasionally moved by up to 5 m in order to minimize differences in habitat features that have previously been found to influence feeding behaviour [43].

All feeding stations had two access holes and were filled with unhusked sunflower seed. The typical behaviour for birds feeding on this resource is to remove the seed and process it in a nearby tree. The design therefore minimized potential interference competition [44], and as these feeders also provided food at a constant rate throughout the study, there were no confounding effects of resource depletion. To allow for natural discovery of sites, each habitat patch was deployed after dark on the evening prior to the start of data collection. Patches were checked from day 2 onwards, and all four feeders removed once any one feeder was fully depleted. If no feeder was fully emptied, then the patch was removed on the morning of the fourth day and data from that day discarded, giving a maximum of three full days of data collection for each replicate.

### (c) Personality assays

Great tits were caught with mist-nets and temporarily taken into captivity at the Wytham field station over two winter seasons (2010–2012). Birds were housed overnight and then individually assayed the following morning in a novel environment with five artificial trees, before being released at the site of capture. Twelve types of behaviour (e.g. flights and hops) were recorded over 8 min, and these were compiled into a principal component (PC) analysis. PC1 described 45% of variation, and the square-root of PC1 was used in a general linear model with individual, time of year and an individual's number of previous assays as fixed effects. This resulted in a single exploration score for each individual, with individuals ranging from slow explorers (SEs) to fast explorers (FEs), also see [29,37,39,41]. Such assays have been conducted in this population since 2005 [37], with good estimates for individual repeatability (*r* = 0.34) [45]; methods are originally based on a design by Verbeek *et al.* [46]. Exploration behaviour is a proxy for the reactive–proactive personality axis in great tits, and has been previously linked to foraging behaviour [41,44].

### (d) Dominance rank

In order to test whether individual variation in collective behaviour was related to dominance, we created an estimated dominance index related to sex, age and body size. We ordered individuals as adult males, juvenile males, adult females and juvenile females, and then ranked individuals within these classes by wing length to generate an overall index, ranging from the largest adult male to the smallest juvenile female. While this is an indirect measure of dominance, there is a long history of studies in great tits to support this rank as estimated [47,48]. Age and sex are the strongest determinants of dominance in tits [49,50], whereas wing length (as a proxy for body size) has also been found to be a good predictor of both dominance and social position in this species [48,51,52]. There is likely to be a further degree of flexibility in this hierarchy related to territory proximity [52,53]; however, any bias should be mitigated by the spatial variability in replicate habitat patches.

### (e) Patch arrivals and feeder choice

We detected the arrival of individuals into the patch by combining the data from the four feeders. Individuals were deemed to arrive when they were first detected on any feeder. Subsequent arrivals were defined as detections after an absence of more than 240 s from the patch. Conversely, birds were deemed to have departed from the patch if not detected within 240 s since their last record on any feeder. We discarded the very first arrival by each individual, because we assumed that prior to that point individuals had no personal information about the patch. For each event, we recorded the feeder on which individuals subsequently arrived and departed from, and the distribution of all other individuals feeding in the patch at that time. We then used Bayes’ rule to calculate the density-dependent probability of arriving (*A*) at a feeder of density *ρ*:2.1
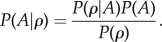


Here *P*(*ρ*) is the frequency (i.e. probability distribution) of observing a density *ρ* taken from the entire dataset of all four feeders; *P*(*A*) is the prior probability of arriving at a given feeder independent of proportion (which we fixed at *P*(*A*) = 1/4 because all feeders were of equal quality) and *P*(*ρ|A*) is the observed frequency of a density *ρ* at feeder where the individual was first detected in a given visit. We provide the posterior probability of arriving given the number of individuals present at a site in the electronic supplementary material, §S4.

### (f) Inferring the decision-making rule used in feeder choice

In order to ascertain whether the exploration behaviour was related to within- or between-individual differences in flocking, we ran generalized linear-mixed models of flock size at arrival against exploration score, with identity as a random effect (electronic supplementary material, §S3). Next, we gained an understanding of the collective behaviour of individuals by fitting the parameters of a previously published model of social decision-making [40]. This model uses three parameters, *s*, *k* and *a.* Parameter *s* refers to the weight that individuals place on the choices made by others. That is, the model assumes when an individual observes a conspecific making a choice, it behaves as if it believes that it makes a ‘good choice’ *s* times more often than a bad choice. Thus, *s* in this study refers to an attraction (i.e. towards the centre of the group) or repulsion (i.e. towards the periphery of the group) [40,54]. Although derived in the context of information about site quality, here *s* in our study could relate to a combination of predation risk and site quality [54], given that birds may not have known that all feeders were identical. Parameter *k* defines how individuals assess the attraction of each option based on the distribution of individuals across them. That is, when *k* = 1, birds make their choices based on the relative difference in the number of conspecifics at each option. When *k* = 0, decisions are made based on the absolute number of individuals on each site. Given flocks vary in size, the biological interpretation of 0 < *k* < 1 may represent birds occasionally choosing less populous sites when the flock is large (following Weber's law [55]). Finally, parameter *a* defines the non-social quality of each food source. Because our patches were made up of four identical feeders that did not deplete, the value of *a* was equal for each choice as there was no *a priori* information suggesting one feeder should be consistently chosen over another. Including *a* in the model had no qualitative effect on our result, and we set *a* = 1; see discussion in [55], e.g. eqn 17.

The effect of *s* and *k* parameters on the probability of an individual choosing a site based on the distribution of conspecifics is given by the following model (from [40]):2.2
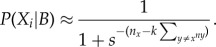


Here, *n_x_* refers to the number of individuals on feeder *x* at time *i*, and *n_y_* is the number of individuals at each of the other feeders at that time. For low values of *s* (at or near 1), this function is a shallow linear increase. As *s* increases, the function becomes sigmoidal, and the probability of picking an empty feeder asymptomatically approaches 0, whereas the probability of picking the most popular feeder asymptomatically approaches 1. We demonstrate the effects of the parameters *s* and *k* in the electronic supplementary material, §S2 and figure S2*a*. The true probability is then acquired by normalizing, and the sites are picked by probability matching, chosen in proportion to equation (2.2) [40,56] (electronic supplementary material, §2, figure S2*b*).

Finally, we calculated the best-fitting value of *s* for each individual separately, while keeping *k* constant. In this case, a *k-*value for the entire population was calculated using maximum-likelihood estimation, where the probability of each observed feeder choice was calculated given the model, summing up the log-likelihoods for each set of given parameter values (using *optim* function in R [57]). Using the likelihood surface, we also calculated the 95% confidence intervals for each individual's estimated value of *s*. Generalized linear models were used to explore the relationship between *s*, personality and dominance rank. Individuals varied in their numbers of arrivals, influencing the uncertainty of the estimated parameter. Therefore, we weighted each value of *s* by the inverse of the size of its 95% CI, but found this had no impact on the estimates of the relationship (electronic supplementary material, §S1*c*).

### (g) Testing if the model replicates the data

We ran 100 simulations, each with 100 arrival events. At each step a new individual arrived into a patch of four identical feeders and a random individual was removed. This maintained a fixed patch-level population size, which we randomly drew from the distribution of group sizes we observed. Arrival density was plotted against the theoretical asocial prediction in the same way as for the empirical data, and a thin plate spline regression was used to fit a surface of this relationship to the individual personality scores. This smoothing algorithm is a generalization of standard splines to multiple dimensions (in this case, 2) [58]; we used the Tps function in the R package *fields* [59].

### (h) Simulations of collective decision-making

Finally, we investigated properties of simulated flocks under different scenarios of *s* distributions. First, we simulated flocks as above with *s* values for each arriving bird either: (i) fixed at the value for the most FE birds, (ii) fixed at the mean value for all individuals, (iii) fixed at the value for the most SE birds, or (iv) *s* randomly sampled from the population distribution. We then tested two group properties be important; (i) diversity of feeder use and (ii) flock cohesion. Diversity of feeder use was defined as how evenly all four feeders were used, measured using the probability of interspecific encounter index [60]. This was scaled to range between 0 and 1, with 1 indicating that all four feeders were used equally. Cohesiveness was defined as the average proportion of individuals that were found in the largest subgroup for each simulation, with 1 indicating that all individuals were always at just one feeder. Four representative simulations were used to create electronic supplementary material, movies A–D. We then compared these results with the same measures taken directly from the empirical data to investigate which scenario best replicated observed flocking behaviour.

## 3. Results

### (a) Flock behaviour

**Figure 1.**
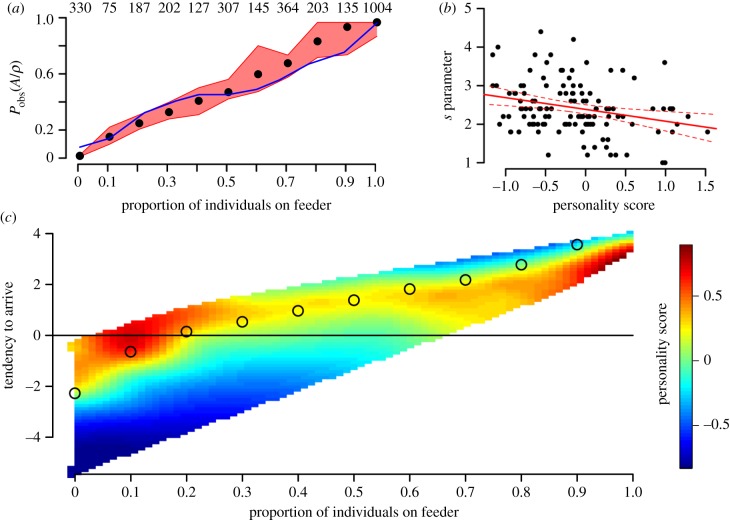
(*a*) Collective behaviour of focal individuals in the population, with birds arriving more often to high-density sites and less often to low-density sites than expected. The *y*-axis is observed probability of arriving, given the density of individuals at the feeder (see equation (2.1)). The red envelope represents maximal range of data from 1000 jack-knife randomizations with 40% of data removed. The blue line is the result of 20 000 simulated arrivals using model with social attraction parameter *s* = 1.93. Numbers represent observed arrivals for each density bin. (*b*) Relationship between-individual personality score (slow-explorer, SE to fast-explorer, FE) and social attraction *s* with 95% CI. (*c*) Mean personality score with respect to foraging location density from 10 000 simulated arrivals for each value of *s*. More SE (blue) individuals have a lower tendency to arrive at low densities and a high tendency to arrive at high densities, whereas more FE (red) individuals have less deviation from random feeder choice. Line = 0 represents expected arrivals in the patch based on random feeder choice, open circles are the observed flock-level data from (*a*), colours are mean personality in simulated data for tendency to arrive at feeders with a given density.

A total of 813 individual great tits were identified over the 20 habitat patches, with 3494 independent patch arrival decisions. These individuals arrived at highly populated feeders more frequently than expected by the theoretical asocial prediction (figure 1*a*). This result was robust to 1000 jack-knife randomizations with 40% of the data removed. Overall, maximum-likelihood estimation resulted in a population-level social attraction parameter (*s*) of 1.93 (95% confidence interval (CI) = 1.79–2.14). Parameter *k* (range 0–1) was estimated as 0.36 (95% CI = 0.31–0.42). That 0 < *k* < 1 suggests that when the number of conspecifics is high, individuals entering the patch consider the relative densities on the feeders rather than the absolute number of other individuals. The patch arrival decisions from 10 000 simulations using parameter values for *s* and *k* replicated the results well and were a good fit to the observed data (figure 1*a*: blue line). This was particularly striking given the simplicity of the predictive model, and suggests that simple interaction rules are sufficient to explain the patterns of collective behaviour observed in the study.

### (b) Individual variation in collective behaviour

A subset of the individuals observed at the habitat patches had also been previously assayed for personality (*n* = 134). In addition to personality scores, dominance rank was estimated for all individuals with known biometrics (*n* = 369). To investigate how this individual variation in dominance and personality influenced flocking behaviour, we calculated the social attraction parameter *s* for each focal individual, keeping *k* constant at the population level (*k* = 0.36). There was no relationship between dominance rank and *s* (coefficient ± s.e. 0.002 ± 0.001, *p* = 0.08; electronic supplementary material, figure S3*a*). However, among individuals with known personalities, exploration behaviour was inversely proportional to the weighting of social attraction, with more reactive (SE) individuals behaving more collectively (coefficient ± s.e. −0.20 ± 0.08, *p* = 0.008; figure 1*b*). There were also marked differences in the distribution; more proactive (FE) individuals fed significantly more at feeders with a low relative density than more reactive (SE) individuals (coefficient ± s.e. −0.07 ± 0.02, *p* < 0.001). We found no relationship between personality and the number of individuals present in the patch (coefficient ± s.e. 0.36 ± 0.87, *p* = 0.41; mean flock size = 6; electronic supplementary material, §S3*b*), feeding rates (coefficient ± s.e. 0.59 ± 0.41, *p* = 0.15; electronic supplementary material, §S1*a*) or flock size at departure (coefficient ± s.e. 0.23 ± 0.23, *p* = 0.33).

### (c) Simulations of group dynamics

**Figure 2.**
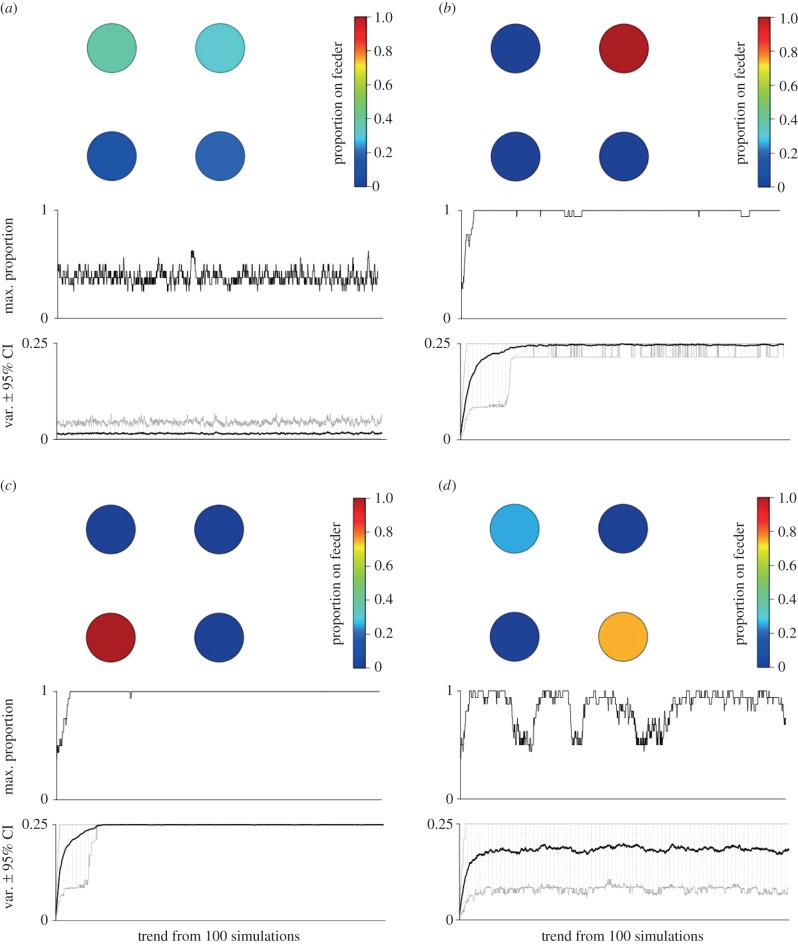
Relationship between group behaviour and personality composition: representative simulations of continuous arrivals and departures in a patch with a fixed population size of six birds, also see the electronic supplementary material, movies (S4). Legend to the right of plots shows colour range indicating proportion of population on a feeder. (*a*) Groups of only fast-exploring (FE) phenotypes never aggregate on a single feeder. (*b*) Groups of only average personality phenotypes fix at a single feeder. (*c*) Groups with slow-exploring (SE) phenotypes behave similarly to groups in (*b*), fixing at a single feeder. (*d*) Groups consisting of individuals with variable personality scores shift from one feeder to another.

**Figure 3.**
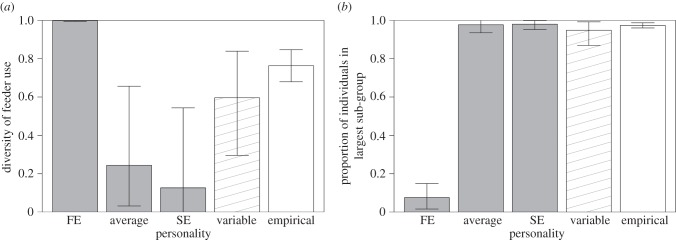
Emergent group behaviour arising from within-group variation in individual personalities, comparing observed data against four sets of simulations. Individuals in simulations were either: all fixed at *s*-value for most proactive individuals (FE); all fixed at average personality phenotype (average); all fixed at *s*-value for most reactive individuals (SE); or randomly sampled from within distribution of personality scores (variable). Horizontal bars are 95% range (simulated data) and 95% CI from 20 replicated habitat patches (empirical data). (*a*) Diversity of feeder use within patches in each simulation type, defined as how evenly all four feeders were used [60]. (*b*) Proportion of individuals in largest subgroup, representing group cohesion. Results highlight importance of intragroup variation in social information use, as also shown by empirical data.

Simulations for 10 000 arrival decisions (sampled across the range of personality scores) resulted in a clear prediction that proactive (FE) individuals should behave less collectively across all densities (figure 1*c*). To investigate how this individual variation in social attraction may affect group foraging behaviour, we then simulated continuous arrivals and departures to four identical feeders in a patch. When groups consisted of all proactive individuals (max. FE, *s* = 1), they rarely aggregated at any one feeder (figure 2*a*), with a high mean diversity of feeder use (figure 3*a*) and very low group cohesion (figure 3*b*). By contrast, groups of all reactive individuals (min. SE, *s* = 4.4) and groups where all individuals had an average phenotype (*s* = 2.2) all rapidly fixed at a single feeder and rarely shifted (figure 2*b,c*), with low diversity of feeder use (figure 3*a*) and very high group cohesiveness (figure 3*b*).

When simulated groups consisted of individuals with variable social attraction parameters (randomly selected from observed personality distribution), they showed a diversity of feeder use much greater than for all average or all SE groups (average group mean = 0.26; variable group mean = 0.58; figure 3*a*). By contrast, group cohesion was similar in variable groups to average groups and all SE groups (average group mean = 0.95; variable group mean = 0.98), and together much higher than FE groups (FE group mean = 0.17; figure 3*b*). This allowed the simulated variable flock to shift from one feeder to another as a largely integrated unit (figure 2*d*). Finally, diversity of feeder use and group cohesion was calculated for empirical data from habitat patches (*n* = 20). In this case, the observed data showed high values for both diversity of feeder use (empirical group mean = 0.76; figure 3*a*) and group cohesion (empirical group mean = 0.97; figure 3*b*), similar to simulated variable groups.

## 4. Discussion

By monitoring movement decisions in wild birds, we obtained evidence from several sources that personality is related to individual variation in collective decision-making. When wild great tits were given a choice of four identical feeders in a habitat patch, all individuals tended to arrive and move to more highly populated feeders, resulting in highly synchronous flocking behaviour. A Bayesian decision-making model of collective behaviour described these individual movements well. Simulations of this model predicted synchronous flocking behaviour, linking the individual- and group-level observations. However, while all individuals exhibited some degree of collective behaviour, it also varied with personality. More reactive (SE) individuals were more likely to choose feeders with a high relative density of individuals and had a higher weighting of social attraction *s*. By contrast, more proactive (FE) individuals were more likely to forage on the spatial periphery of flocks, and move away from areas of high density. This was independent of their estimated dominance rank, flock size or feeding rates, suggesting that there was no effect of competition or neophobia on social behaviour, despite being positively (competitiveness) or negatively (neophobia) correlated with exploration behaviour in some other contexts [44,61].

Our study is, to our knowledge, the first to show such a relationship between personality and individual differences in collective behaviour in naturally occurring wild groups. Previous captive experiments in sticklebacks, sheep and geese [7,12,24,25] also support the direction of these results, and suggest a pattern whereby reactive individuals have a greater social attraction to conspecifics and are more likely to use social information. This pattern could even potentially extend to producer–scrounger dynamics, with some studies suggesting that shy individuals are more likely to scrounge (e.g. in geese [62]). Our results are further consistent with our previous research on social behaviour of great tits, where reactive (SE) individuals tended to have stronger and more temporally stable social network associations [29], and may suggest one potential simple mechanism by which such longer-term population-level patterns may be obtained. We have extended the scope of this previous research in a number of ways by quantifying this relationship over 2 years and over a large spatial scale, in a fission–fusion population where foraging flocks are comprised naturally associating individuals of variable personalities.

Personality in great tits is thought to relate to a differential response to risk-taking, with proactive (FE) birds engaging in potentially highly rewarding behaviour with an higher associated risk, and more reactive (SE) individuals favouring a lower productivity but low-risk strategy. Flocking is also thought to be a response to shifting levels of predation and resource availability, and the observed differences in grouping tendencies are consistent with this trade-off. Great tits are vulnerable to predation from Eurasian sparrowhawks (*Accipiter nisus*) [63], which are attracted to flocks forced to aggregate at patchy food sources. By showing a high degree of social attraction towards flock mates and increasing group-level synchrony, reactive (SE) individuals may be lowering their predation risk. While in our habitat patches food was not limited, we would generally expect such behaviour to lead to an increase in competition between group members, thus leading to a risk/productivity trade-off in social behaviour that individuals may weigh differently depending on personality type.

We investigated the consequences of individual variation in social behaviour on group decision-making by simulating the patch-level behaviour of average-sized groups comprising different combinations of personality types. As expected, when groups consisted all of extreme phenotypes, they either concentrated at a single foraging location (when all reactive), or dispersed towards the lowest possible density (when all proactive). However, interestingly, when groups were comprised a single personality phenotype taken from the population average, they exhibited collective behaviour similar to reactive groups, concentrating at high densities with little movement. Only groups containing variable personalities (either from empirical data or taken randomly from the observed distribution) showed both group cohesiveness and patch exploration. In this way, our results support the theoretical predictions of a leader–follower polymorphism [12,14,15], with a small proportion of very exploratory individuals allowing for collective action while overall group coordination is maintained [14,17]. They also reflect recent studies in insects and social spiders, where groups consisting of variable behavioural types showed better overall success in measures such as foraging or reproduction [30–34].

There is an increasing body of evidence that social behaviour and collective decision-making may not just reflect immediate costs and benefits, but may also be an outcome of intrinsic behavioural differences between individuals [15,29,32]. We use novel automated data-collection methods to show that the well-understood personality trait ‘exploration behaviour’ is related to individual differences in both grouping tendencies and collective behaviour in wild great tits. Such differences should impact collective decision-making processes in groups, and we use computational models based on our empirical data to demonstrate that groups consisting of variable personality types show the most effective coordinated action when exploiting a habitat patch. Further research should aim to provide empirical support for the predictions of this model, manipulating the personality composition of large groups to observe how it influences patch exploitation and group movements. We demonstrate an experimental paradigm that can be generalized to allow collective behaviour research such as this to be conducted in wild naturalistic contexts, helping improve our understanding of the evolution and ecology of social behaviour.

## Supplementary Material

Electronic Supplementary Material

## Supplementary Material

ESM-Data

## Notes

Work was subjected to review by the Department of Zoology ethical committee, University of Oxford, and the Animal Experimentation Ethics Committee, Australian National University. Birds were held in captivity under Natural England licences 20104175 and 20114175.

## Data accessibility

Data is available as the electronic supplementary material.
