# Supplementary material for: Individual-level personality influences social foraging and collective behaviour in wild birds
Source: Proc Biol Sci. 2014 Aug 22;281(1789):20141016. doi: 10.1098/rspb.2014.1016 (PMC4100518; doi:10.1098/rspb.2014.1016)
Supplement: Electronic Supplementary Material [file rspb20141016supp1.docx]

**SUPPLEMENTARY MATERIAL:**

**S1.** Discussion of Possible Biases in Data Collection

*Variation in neophobic tendencies towards feeders*

Some previous research suggests that individuals of differing personality type may show variation in neophobic responses towards novel objects, with reactive individuals being more neophobic. If the four feeders installed in the artificial habitat patches were considered novel objects by feeding great tits, then it is possible that differences in collective behaviour and feeder choice could be influenced by differences in neophobia. We tested this possible bias in our data collection in three alternative ways. Firstly, changes in visitation rates were compared to personality scores, with the assumption that if reactive individuals were initially more neophobic, they should shower a greater relative increase in visitation rates from day 1 to day 2 than more proactive individuals (Table S1a). Secondly, the analysis was repeated with only data derived from the second and third day of data collection in each replicate (Table S1b). Thirdly, the individuals' value of *S* in the general linear model were weighted by the inverse of the confidence intervals calculated by the likelihood curve and the model rerun in order to control for differences in repeatability of individual arrival patterns (Table S1c). None of the three analyses provided any evidence for an effect of neophobia on feeding patterns.

*Overall proportion and distribution of PIT-tagged individuals*

The great tit population in Wytham Woods is comprised of two components: i) birds born in a nest box and fitted with PIT tags as nestlings; and ii) immigrant birds caught and fitted with a PIT tag as an adult. There are c. 1200 nest boxes in Wytham Woods that are monitored each spring in which almost every chick and adult caught breeding have been fitted with a PIT tag since 2007. In addition, intensive periods of mist-netting have been conducted throughout the winter in order to catch and PIT tag immigrant birds. In the autumn and winter of 2010/11, mist-netting was conducted 6 days per week for three months in order to capture all individuals present and ensure near 100% coverage by the time this study commenced. In the following winter, mist-netting was conducted one week per month in order to maintain the proportion of birds PIT tagged. Replicate habitat patches were always located at sites where recent intensive mist-netting had been conducted. In order to quantify the proportion of individuals fitted with PIT tags in the population, we calculated the proportion of unmarked birds in the breeding season immediately after the two winters. This gives the best estimate since each individual is captured only once, but is likely to over-estimate the proportion of unmarked individuals due to a final movement of immigrants into the woods just before breeding, when no mist-netting is conducted. In the spring of 2011, 17% of the birds (80 of 389) were untagged, while 8% of birds were untagged in the spring of 2012. Given that the replicates were conducted within areas that had recent catching effort, we estimate that significantly more than 90% of individuals in our study were tagged.

**S1a.** Generalised linear mixed model (GLMM) output shows no relationship between personality and the number of visits. There was a significant effect of day, with individuals visiting more often on the second day. However, the interaction term between day and personality suggests that there was no statistical difference in the visitation patterns between slow explorers and fast explorers. Individual identity explained 73% of the variance and we used a Poisson error distribution.

| **Model** | **Effect** | **Std Err** | ***P*** |
| --- | --- | --- | --- |
| Personality | 0.59 | 0.41 | 0.15 |
| + Day | 0.87 | 0.18 | <0.001 |
| + Personality * Day | -0.50 | 0.31 | 0.11 |
|  |  |  |  |

**S1b.** General linear model (GLM) output shows a consistent relationship between individuals' values of *S* and personality when data from the first day was excluded (n = 110 individuals with sufficient data for maximum likelihood estimation of *S*).

| **Model** | **Effect** | **Std Err** | ***P*** |
| --- | --- | --- | --- |
| Personality | -0.24 | 0.08 | 0.004 |
| + Count | 0.004 | 0.004 | 0.27 |
|  |  |  |  |

**S1c.** General linear model (GLM) output shows a consistent and significant relationship between individuals' values of *S* and personality when each point in the model was weighted by the inverse of the confidence intervals estimated from the shape of the negative log-likelihood curve. As the influence of changes in *S* decreases exponentially as *S* increases, we used the residual of the confidence interval from each point when fitted with an exponential model.

| **Model** | **Effect** | **Std Err** | ***P*** |
| --- | --- | --- | --- |
| Personality | -0.12 | 0.04 | 0.001 |
|  |  |  |  |

**S2.** Influence of the parameters *s* and *k* on the shape of the decision-making response by individuals given a choice of two feeders. (a) gives the probability that an individual sees option x as being a ‘good choice’ as a function of the number of conspecifics (given by equation 2 in the main text). (b) gives the probability of choosing option x after applying probability matching. Parameter *k* affects the offset of the curves from an even probability, such that low *k* values lead to a wider band of proportions at which the probability is equal for both sites. The *k* parameter was kept constant when estimating *S* at the individual level. Parameter *s* influences the shape of the response, in particular, low values of *s* have a smaller difference in the probability of choosing sites with a low or high proportions of individuals. When *k* < 1, we apply an additional step of probability matching, resulting in a potentially large area of indifference at medium densities.

**S3.** Model outputs for three social measures, personality score and dominance rank. Model includes data across 20 replicated experiments in 2010-2012 and *S* is estimated including all other conspecifics in patch.

**S3a**. General linear model comparing the social attraction parameter *S* for each focal individual (N = 134) with individual personality score, number of visits, and dominance rank. Personality and *S* parameter are negatively correlated. There is no relationship between the *S* parameter and dominance or visit count.

| **Test** | **Model** | **Effect** | **Std Err** | ***P*** |
| --- | --- | --- | --- | --- |
| **Parameter *S*** | | | | |
|  | Personality | -0.19 | 0.09 | 0.03 |
|  | Dominance Rank | 0.002 | 0.001 | 0.08 |
|  | Count | -0.005 | 0.003 | 0.08 |
|  |  |  |  |  |

**S3b**. General linear mixed models comparing the proportion of individuals at a feeder (relative local density) and the overall group size with individual personality score. N = 3494 arrivals, Individual ID is included as a random effect. While there is a negative relationship between personality and attraction to feeders with a relatively high proportion of individuals, there is no effect of personality on arrival decisions given the total number of individuals in the patch.

| **Test Model** | | | **Effect Std Err *P*** | | |
| --- | --- | --- | --- | --- | --- |
| **Proportion of individuals at feeder** | | |  | | |
|  | Personality | -0.07 | | 0.02 | <0.01 |
|  |  |  | |  |  |
| **Group size at patch** | | | | | |
|  | Personality | 0.36 | | 0.87 | 0.41 |
|  |  |  | |  |  |

**S4.** Probability of arriving at a feeder given the number of individuals present on the feeder. Each point is the observed probability of arriving at a feeder given the number of individuals on the feeder irrespective of the number of individuals on other feeders. This figure shows a sharp increase in the probability of arriving at a feeder that reaches 0.5 when just two conspecifics are present, and remains above 0.5 as the number of individuals reaches and exceeds the mean number of conspecifics in the patch (6). The numbers above each point represent the sample size (arrivals at feeders with that density).
