# Supplementary material for: Individual-level personality influences social foraging and collective behaviour in wild birds
Source: Proc Biol Sci. 2014 Aug 22;281(1789):20141016. doi: 10.1098/rspb.2014.1016 (PMC4100518; doi:10.1098/rspb.2014.1016)
Supplement: ESM-Data [file rspb20141016supp2.pdf]

ID	PERSONALIT	ARRIVAL.SIT	SITE1.N	SITE2.N	SITE3.N	SITE4.N
1	-0.4769452	4	9	0	0	0
1	-0.4769452	4	5	0	0	3
1	-0.4769452	1	0	0	0	6
1	-0.4769452	1	0	0	0	6
1	-0.4769452	1	5	0	0	0
1	-0.4769452	1	2	2	0	0
1	-0.4769452	3	0	10	0	0
1	-0.4769452	1	1	8	0	0
1	-0.4769452	3	3	3	2	0
1	-0.4769452	1	2	3	0	0
1	-0.4769452	1	2	0	4	2
1	-0.4769452	4	0	0	1	2
1	-0.4769452	4	1	2	1	3
1	-0.4769452	4	2	2	2	0
1	-0.4769452	1	0	0	0	6
1	-0.4769452	1	10	0	0	0
1	-0.4769452	1	3	0	1	0
1	-0.4769452	1	4	0	2	0
1	-0.4769452	3	0	0	15	0
1	-0.4769452	3	0	0	5	0
1	-0.4769452	3	0	0	7	0
1	-0.4769452	3	1	0	5	0
1	-0.4769452	3	0	0	4	0
1	-0.4769452	3	0	0	9	0
1	-0.4769452	3	0	0	9	0
1	-0.4769452	1	2	1	3	0
1	-0.4769452	3	0	0	8	0
1	-0.4769452	3	0	0	12	0
1	-0.4769452	3	0	2	8	0
1	-0.4769452	4	0	0	0	2
1	-0.4769452	3	0	0	10	0
2	-1.0982582	2	0	0	0	0
2	-1.0982582	2	0	8	0	0
2	-1.0982582	2	0	9	0	0
2	-1.0982582	2	6	3	0	0
2	-1.0982582	2	0	8	0	0
2	-1.0982582	2	0	7	0	0
2	-1.0982582	2	1	8	0	0
2	-1.0982582	3	2	2	0	0
2	-1.0982582	4	0	0	10	0
2	-1.0982582	2	0	7	0	0
2	-1.0982582	2	0	0	0	0
2	-1.0982582	3	0	0	5	3
2	-1.0982582	3	0	0	4	0
2	-1.0982582	3	0	0	2	0
2	-1.0982582	3	0	0	6	0
2	-1.0982582	3	0	0	6	2
2	-1.0982582	3	0	0	6	0
2	-1.0982582	3	0	0	7	0
2	-1.0982582	3	0	0	2	0
2	-1.0982582	3	0	0	12	0
2	-1.0982582	4	0	0	0	12
2	-1.0982582	4	0	0	0	5
2	-1.0982582	3	0	2	6	0
2	-1.0982582	4	0	0	2	6
3	-0.2037715	2	8	0	0	0
3	-0.2037715	2	2	4	0	0
3	-0.2037715	2	3	6	0	0
3	-0.2037715	3	2	3	2	0
3	-0.2037715	3	2	2	5	0
3	-0.2037715	3	0	0	7	0
3	-0.2037715	3	0	0	3	0
3	-0.2037715	2	0	0	0	0
3	-0.2037715	2	0	0	0	0
3	-0.2037715	2	0	0	0	0
3	-0.2037715	2	6	1	0	2
3	-0.2037715	3	10	0	0	0
3	-0.2037715	3	1	0	3	3
3	-0.2037715	3	1	0	1	5
3	-0.2037715	2	0	6	0	0
3	-0.2037715	3	4	0	0	0
3	-0.2037715	3	10	0	0	0
3	-0.2037715	3	0	0	6	0
3	-0.2037715	3	0	0	3	0
3	-0.2037715	3	0	0	5	0
3	-0.2037715	2	0	0	0	0
3	-0.2037715	3	3	0	6	0
3	-0.2037715	2	4	2	4	0
3	-0.2037715	2	0	0	0	0
3	-0.2037715	3	2	1	6	0
3	-0.2037715	3	0	6	1	0
3	-0.2037715	2	0	2	5	0
3	-0.2037715	3	0	1	9	0
3	-0.2037715	3	0	0	5	0
3	-0.2037715	3	0	0	5	0
3	-0.2037715	4	0	0	0	11
3	-0.2037715	3	0	0	1	7
4	0.58324324	2	0	4	1	0
4	0.58324324	3	3	2	3	0
4	0.58324324	3	0	0	0	0
4	0.58324324	2	4	0	1	0
4	0.58324324	3	2	5	0	0
4	0.58324324	2	3	4	0	0
4	0.58324324	2	12	0	0	0
4	0.58324324	1	5	0	0	0
4	0.58324324	1	5	0	0	4
4	0.58324324	1	8	0	1	0
4	0.58324324	1	5	0	0	0
4	0.58324324	3	0	0	4	3
4	0.58324324	3	0	0	0	0
4	0.58324324	3	0	0	6	0
4	0.58324324	3	0	0	1	0
4	0.58324324	3	1	0	7	0
4	0.58324324	3	0	0	5	0
4	0.58324324	3	9	0	0	0
4	0.58324324	1	0	4	2	0
5	-0.596579	3	0	0	6	0
5	-0.596579	3	0	0	8	2
5	-0.596579	3	0	7	0	0
5	-0.596579	1	4	3	1	0
5	-0.596579	3	0	0	3	5
5	-0.596579	3	0	0	0	4
5	-0.596579	2	0	8	0	0
5	-0.596579	1	2	0	0	3
5	-0.596579	1	8	0	1	0
5	-0.596579	1	0	0	0	0
5	-0.596579	3	0	0	7	0
5	-0.596579	3	0	0	7	0
5	-0.596579	3	0	0	10	0
5	-0.596579	3	1	0	5	0
5	-0.596579	3	0	0	5	0
5	-0.596579	3	0	0	3	0
5	-0.596579	3	0	0	5	0
5	-0.596579	3	7	0	2	0
5	-0.596579	3	2	1	4	0
5	-0.596579	3	0	4	2	0
5	-0.596579	2	0	7	0	0
5	-0.596579	3	0	0	6	0
5	-0.596579	3	0	4	3	0

ID	PERSONALIT	ARRIVAL.SIT	SITE1.N	SITE2.N	SITE3.N	SITE4.N
57	0.46721311	1	4	0	0	4
57	0.46721311	1	0	0	0	0
57	0.46721311	1	9	0	0	0
57	0.46721311	1	0	0	0	9
57	0.46721311	4	0	0	0	3
58	0.44441689	1	8	0	0	0
58	0.44441689	1	11	0	0	0
58	0.44441689	1	0	0	0	0
58	0.44441689	1	6	0	0	0
58	0.44441689	1	5	0	0	0
58	0.44441689	4	3	0	0	1
58	0.44441689	3	0	0	0	6
58	0.44441689	4	0	0	0	2
58	0.44441689	4	0	0	0	0
58	0.44441689	1	0	0	0	0
58	0.44441689	4	6	0	0	0
58	0.44441689	4	0	0	0	5
58	0.44441689	4	0	0	0	7
58	0.44441689	4	0	0	0	8
58	0.44441689	1	4	1	0	2
58	0.44441689	4	4	0	0	2
58	0.44441689	4	1	0	0	4
58	0.44441689	1	4	0	0	3
58	0.44441689	1	3	0	0	4
58	0.44441689	1	6	3	0	0
58	0.44441689	1	9	0	0	0
58	0.44441689	1	5	0	0	0
59	-0.6506817	1	0	0	0	0
59	-0.6506817	2	0	0	0	0
59	-0.6506817	1	4	3	0	0
59	-0.6506817	4	0	0	0	12
59	-0.6506817	4	7	0	0	0
59	-0.6506817	4	0	0	0	7
59	-0.6506817	4	0	0	8	0
59	-0.6506817	4	0	0	0	6
59	-0.6506817	4	5	0	0	2
59	-0.6506817	4	0	0	0	3
59	-0.6506817	4	0	0	0	0
59	-0.6506817	4	3	0	0	1
59	-0.6506817	4	7	0	0	0
59	-0.6506817	4	0	0	0	0
59	-0.6506817	4	5	0	0	2
59	-0.6506817	4	0	0	0	6
59	-0.6506817	4	2	0	0	2
59	-0.6506817	4	3	0	0	1
59	-0.6506817	4	0	0	0	0
59	-0.6506817	4	0	0	0	3
59	-0.6506817	4	0	0	0	5
59	-0.6506817	4	0	0	0	9
59	-0.6506817	4	0	0	0	6
59	-0.6506817	4	2	0	0	4
59	-0.6506817	4	2	0	0	5
59	-0.6506817	4	2	0	0	7
59	-0.6506817	1	4	0	2	0
59	-0.6506817	1	3	0	2	0
59	-0.6506817	1	5	0	0	0
59	-0.6506817	4	4	0	0	3
59	-0.6506817	4	0	0	0	0
59	-0.6506817	1	0	0	0	0
59	-0.6506817	1	6	0	0	0
59	-0.6506817	1	7	1	0	0
59	-0.6506817	1	5	0	0	0
59	-0.6506817	1	9	0	0	0
59	-0.6506817	1	3	0	0	0
59	-0.6506817	4	0	0	0	3
60	0.26196807	1	5	0	0	0
60	0.26196807	4	0	0	0	0
60	0.26196807	4	0	0	0	0
60	0.26196807	4	0	0	0	11
60	0.26196807	4	0	0	0	8
60	0.26196807	4	0	0	0	2
60	0.26196807	4	0	0	0	11
60	0.26196807	4	3	0	0	6
60	0.26196807	4	7	0	0	0
60	0.26196807	4	0	0	0	3
60	0.26196807	4	0	0	0	3
60	0.26196807	4	0	0	0	6
60	0.26196807	4	2	0	0	1
60	0.26196807	3	1	0	1	2
60	0.26196807	4	1	0	0	7
60	0.26196807	4	1	0	0	4
60	0.26196807	4	9	0	1	0
60	0.26196807	4	0	0	0	0
60	0.26196807	2	7	0	0	0
60	0.26196807	2	14	0	0	0
60	0.26196807	1	7	0	0	0
60	0.26196807	4	7	0	0	0
60	0.26196807	4	3	0	0	5
60	0.26196807	4	0	0	0	7
61	-0.2053642	4	8	0	0	0
61	-0.2053642	4	0	0	0	5
61	-0.2053642	4	1	0	0	6
61	-0.2053642	4	0	0	0	8
61	-0.2053642	4	0	0	0	5
61	-0.2053642	4	0	0	0	7
61	-0.2053642	4	0	0	0	10
61	-0.2053642	4	2	0	0	8
61	-0.2053642	4	2	0	0	6
61	-0.2053642	4	2	0	2	3
61	-0.2053642	4	0	0	0	6
61	-0.2053642	4	0	0	0	5
61	-0.2053642	4	3	0	0	1
61	-0.2053642	1	8	0	0	0
61	-0.2053642	1	8	0	0	0
61	-0.2053642	1	6	0	0	0
61	-0.2053642	1	1	1	0	0
61	-0.2053642	1	7	0	0	0
61	-0.2053642	1	6	0	0	0
61	-0.2053642	1	8	0	0	2
61	-0.2053642	4	0	0	0	11
62	-0.8150679	4	7	0	0	2
62	-0.8150679	1	0	0	0	4
63	-0.4580645	4	0	0	0	13
63	-0.4580645	4	3	0	0	5
63	-0.4580645	1	9	0	0	0
63	-0.4580645	4	2	0	0	5
63	-0.4580645	4	0	0	0	5
63	-0.4580645	4	0	0	0	4
63	-0.4580645	4	7	0	0	0
63	-0.4580645	4	2	0	0	4
63	-0.4580645	4	2	0	0	2
63	-0.4580645	4	2	0	0	5
63	-0.4580645	4	0	0	0	10
63	-0.4580645	1	8	0	0	0
63	-0.4580645	1	4	0	0	0
63	-0.4580645	1	4	2	0	0
5	-0.596579	2	5	0	0	4
5	-0.596579	1	0	0	0	9
5	-0.596579	1	0	0	0	13

ID	PERSONALIT	ARRIVAL.SIT	SITE1.N	SITE2.N	SITE3.N	SITE4.N
98	0.41604249	2	0	6	3	0
98	0.41604249	2	0	4	6	0
98	0.41604249	2	0	6	1	0
98	0.41604249	2	0	5	0	0
98	0.41604249	2	0	5	0	0
98	0.41604249	3	1	6	2	0
98	0.41604249	2	0	6	1	0
98	0.41604249	3	0	6	1	0
98	0.41604249	2	0	6	1	0
98	0.41604249	2	0	1	6	0
98	0.41604249	3	0	0	1	0
98	0.41604249	2	0	7	0	0
99	-0.3058689	2	0	3	2	0
99	-0.3058689	2	0	3	3	0
99	-0.3058689	2	0	9	0	0
99	-0.3058689	2	0	6	1	0
99	-0.3058689	2	0	4	1	0
99	-0.3058689	3	0	3	4	0
100	-0.2018082	2	0	3	3	0
100	-0.2018082	2	0	4	3	0
100	-0.2018082	3	0	5	1	0
100	-0.2018082	3	0	5	1	0
100	-0.2018082	2	0	2	3	1
100	-0.2018082	3	0	0	6	0
101	-0.5603354	2	0	7	0	0
101	-0.5603354	3	0	0	0	0
101	-0.5603354	2	0	0	0	7
101	-0.5603354	4	0	0	0	9
102	0.31547231	2	0	7	0	2
102	0.31547231	2	0	9	0	0
102	0.31547231	2	0	3	2	0
102	0.31547231	2	0	2	6	1
102	0.31547231	4	0	0	0	5
102	0.31547231	4	0	0	0	7
102	0.31547231	4	0	0	0	13
102	0.31547231	4	0	0	0	6
102	0.31547231	4	0	2	0	8
102	0.31547231	4	0	0	0	7
102	0.31547231	4	0	2	6	0
103	-0.6086034	3	0	6	4	0
103	-0.6086034	3	0	0	7	0
103	-0.6086034	2	0	0	0	6
103	-0.6086034	4	0	0	0	5
103	-0.6086034	4	0	0	1	4
104	-0.7008536	2	0	3	4	1
104	-0.7008536	4	0	0	0	9
104	-0.7008536	4	0	0	0	9
5	-0.596579	4	0	0	0	0
5	-0.596579	3	0	0	3	0
5	-0.596579	3	0	0	0	0
5	-0.596579	3	0	0	0	0
5	-0.596579	4	0	0	0	0
5	-0.596579	4	0	0	0	0
5	-0.596579	3	0	0	0	0
105	0.02320027	4	0	0	0	11
105	0.02320027	4	0	0	0	0
105	0.02320027	3	0	0	0	0
105	0.02320027	3	0	0	5	0
105	0.02320027	1	0	0	0	0
105	0.02320027	1	3	0	0	0
105	0.02320027	4	0	0	9	0
63	-0.4580645	1	0	0	0	0
63	-0.4580645	3	4	4	0	0
63	-0.4580645	3	0	0	0	0
63	-0.4580645	3	0	0	6	0
63	-0.4580645	3	0	0	8	0
88	-0.088144	3	3	2	4	0
106	-0.5067385	1	0	0	0	0
107	0.01269119	3	0	0	0	0
107	0.01269119	4	1	0	1	3
107	0.01269119	4	2	0	1	3
107	0.01269119	4	0	0	0	16
107	0.01269119	4	0	0	0	5
107	0.01269119	2	0	6	1	0
107	0.01269119	4	0	0	7	2
107	0.01269119	4	0	0	3	6
107	0.01269119	4	0	0	1	5
107	0.01269119	4	0	0	5	2
107	0.01269119	4	0	0	2	3
107	0.01269119	4	0	0	0	4
107	0.01269119	4	0	0	2	2
107	0.01269119	4	0	0	1	10
107	0.01269119	4	0	0	0	6
107	0.01269119	4	0	0	0	3
86	-0.9389541	4	0	0	0	0
86	-0.9389541	4	0	0	5	0
86	-0.9389541	4	0	0	5	3
86	-0.9389541	4	0	0	0	7
86	-0.9389541	2	4	1	1	3
86	-0.9389541	4	0	0	1	4
86	-0.9389541	2	0	5	0	4
86	-0.9389541	3	0	0	1	7
86	-0.9389541	2	0	1	1	4
86	-0.9389541	1	3	4	0	2
86	-0.9389541	2	4	0	0	0
86	-0.9389541	4	0	0	0	9
86	-0.9389541	3	0	0	7	1
86	-0.9389541	4	0	0	3	4
86	-0.9389541	4	0	0	2	2
86	-0.9389541	4	0	0	0	10
86	-0.9389541	1	0	0	4	1
86	-0.9389541	4	0	0	0	5
86	-0.9389541	1	1	0	2	3
86	-0.9389541	4	1	0	0	6
86	-0.9389541	4	0	0	0	12
86	-0.9389541	4	0	0	0	5
86	-0.9389541	4	0	0	0	8
6	0.04574931	4	0	0	0	6
6	0.04574931	2	0	0	0	5
6	0.04574931	4	1	0	0	3
6	0.04574931	1	3	3	0	0
6	0.04574931	2	0	6	1	0
6	0.04574931	4	0	0	1	8
6	0.04574931	1	6	0	0	0
6	0.04574931	4	0	0	0	9
6	0.04574931	1	1	0	3	1
6	0.04574931	4	0	0	3	5
84	-1.0300019	3	0	0	7	4
84	-1.0300019	1	1	0	1	5
84	-1.0300019	1	3	0	1	4
84	-1.0300019	1	4	0	0	1
84	-1.0300019	1	2	3	0	2
84	-1.0300019	2	0	7	1	1
84	-1.0300019	1	2	4	0	2
84	-1.0300019	4	0	0	3	8
84	-1.0300019	4	0	0	0	3
84	-1.0300019	3	0	0	4	2
84	-1.0300019	3	0	0	2	2
84	-1.0300019	1	4	0	0	0

5	-0.596579	3	0	0	7	0
5	-0.596579	3	0	0	6	0
5	-0.596579	3	0	0	9	0
5	-0.596579	3	0	0	10	0
5	-0.596579	3	0	0	9	1
5	-0.596579	4	0	0	1	8
5	-0.596579	4	0	0	0	9
5	-0.596579	4	0	0	2	8
5	-0.596579	4	0	0	0	7
6	0.04574931	1	4	0	4	0
6	0.04574931	3	0	0	7	0
6	0.04574931	3	0	0	12	0
6	0.04574931	4	0	0	0	8
6	0.04574931	4	0	0	1	8
6	0.04574931	4	0	0	0	9
6	0.04574931	4	0	0	1	4
7	0.33864873	1	2	4	0	0
7	0.33864873	1	3	3	5	0
7	0.33864873	2	0	4	0	0
7	0.33864873	3	0	0	12	0
7	0.33864873	3	0	0	9	0
7	0.33864873	3	0	0	5	0
7	0.33864873	3	0	0	0	11
7	0.33864873	4	0	0	1	6
7	0.33864873	4	0	0	2	6
8	0.20181715	3	0	0	0	0
8	0.20181715	3	0	0	0	0
8	0.20181715	3	0	0	0	0
8	0.20181715	2	0	3	0	0
8	0.20181715	2	0	0	0	0
8	0.20181715	2	0	9	0	0
8	0.20181715	2	0	0	0	0
8	0.20181715	3	0	9	0	0
8	0.20181715	3	0	0	0	0
8	0.20181715	3	0	0	0	0
8	0.20181715	3	0	0	7	0
8	0.20181715	3	0	0	0	0
8	0.20181715	3	0	8	0	0
8	0.20181715	3	0	0	0	0
8	0.20181715	3	0	0	0	0
8	0.20181715	3	0	0	0	0
8	0.20181715	3	0	0	0	0
8	0.20181715	3	0	0	2	4
8	0.20181715	3	0	0	0	0
8	0.20181715	3	0	0	10	0
8	0.20181715	3	0	0	4	4
8	0.20181715	2	0	3	0	0
8	0.20181715	2	0	0	0	0
8	0.20181715	2	0	0	9	0
8	0.20181715	2	0	0	10	0
8	0.20181715	2	0	2	5	0
8	0.20181715	2	0	0	0	0
8	0.20181715	3	0	0	4	0
8	0.20181715	2	0	4	2	0
8	0.20181715	2	0	0	10	0
8	0.20181715	2	0	0	10	0
8	0.20181715	2	0	0	4	0
8	0.20181715	2	0	0	1	1
8	0.20181715	2	0	0	4	0
8	0.20181715	3	0	0	4	0
8	0.20181715	3	0	0	6	0
8	0.20181715	2	0	0	0	8
8	0.20181715	4	0	0	0	7
8	0.20181715	1	0	0	0	14
8	0.20181715	2	0	0	0	4
8	0.20181715	4	0	0	0	7
9	0.12832241	2	0	0	0	0
9	0.12832241	2	0	0	0	0
9	0.12832241	2	0	14	0	0
9	0.12832241	2	0	0	0	0
9	0.12832241	2	0	0	0	0
9	0.12832241	2	0	0	0	0
9	0.12832241	3	0	0	0	0
9	0.12832241	3	0	7	0	0
9	0.12832241	3	0	0	4	4
9	0.12832241	3	0	0	0	0
9	0.12832241	2	0	5	0	0
9	0.12832241	3	0	1	6	0
9	0.12832241	3	0	0	8	0
9	0.12832241	3	0	0	9	0
9	0.12832241	4	0	2	0	5
9	0.12832241	4	0	0	0	0
9	0.12832241	4	0	0	0	6
9	0.12832241	4	0	0	0	0
10	-0.5315071	3	0	0	0	0
10	-0.5315071	2	0	0	8	0
10	-0.5315071	2	0	0	0	0
10	-0.5315071	2	0	0	10	0
10	-0.5315071	3	0	0	0	0
10	-0.5315071	3	0	0	0	0
10	-0.5315071	3	0	0	0	0
10	-0.5315071	3	0	0	0	0
10	-0.5315071	3	0	0	0	0
10	-0.5315071	3	0	0	0	0
10	-0.5315071	2	0	0	0	0
10	-0.5315071	3	0	0	3	1
10	-0.5315071	2	0	0	0	9
10	-0.5315071	2	0	0	0	0
10	-0.5315071	3	0	0	0	4
10	-0.5315071	3	0	0	0	0
10	-0.5315071	3	0	2	2	4
10	-0.5315071	3	0	8	0	0
10	-0.5315071	3	0	0	0	0
10	-0.5315071	3	0	0	0	0
10	-0.5315071	2	0	1	2	3
10	-0.5315071	3	0	0	4	2
10	-0.5315071	3	0	0	3	0
10	-0.5315071	3	0	0	8	0
10	-0.5315071	4	0	0	0	9
10	-0.5315071	4	0	7	0	0
10	-0.5315071	4	0	0	0	5
10	-0.5315071	4	0	0	0	9
10	-0.5315071	4	1	0	0	2
10	-0.5315071	4	0	0	0	8
10	-0.5315071	4	0	1	0	8
10	-0.5315071	4	0	3	0	7
10	-0.5315071	4	0	0	0	10
10	-0.5315071	4	0	0	0	6
11	-0.776814	3	0	0	13	0
11	-0.776814	3	0	0	0	0
11	-0.776814	3	0	0	8	0
11	-0.776814	4	0	0	0	0
11	-0.776814	4	0	0	0	4
11	-0.776814	4	0	0	0	5
11	-0.776814	4	0	0	0	6
11	-0.776814	4	1	0	0	6
11	-0.776814	4	0	0	0	5
11	-0.776814	4	0	4	0	6
11	-0.776814	4	0	0	0	9
11	-0.776814	4	0	0	0	5

5	-0.596579	4	3	0	0	5
5	-0.596579	4	4	0	0	0
5	-0.596579	4	4	0	0	4
5	-0.596579	4	3	0	0	3
5	-0.596579	3	0	0	0	0
5	-0.596579	4	7	0	0	0
5	-0.596579	1	6	0	0	0
5	-0.596579	1	9	0	0	0
13	-0.6454721	1	6	2	0	0
13	-0.6454721	1	9	0	0	0
13	-0.6454721	1	9	0	0	0
13	-0.6454721	1	7	0	0	0
13	-0.6454721	1	6	2	0	0
13	-0.6454721	1	2	3	0	2
13	-0.6454721	1	1	0	0	3
13	-0.6454721	4	0	0	0	4
64	-0.5598805	1	9	0	0	0
64	-0.5598805	2	0	9	0	0
64	-0.5598805	2	2	0	0	5
64	-0.5598805	1	5	0	0	0
64	-0.5598805	1	3	0	0	0
65	-0.5017981	4	0	2	0	3
65	-0.5017981	4	5	0	0	4
66	-0.9346919	1	15	0	0	0
66	-0.9346919	2	0	4	0	0
66	-0.9346919	1	13	0	0	0
66	-0.9346919	1	0	0	4	0
66	-0.9346919	1	0	0	5	0
66	-0.9346919	1	0	0	4	0
66	-0.9346919	1	4	0	4	0
66	-0.9346919	1	0	0	0	0
66	-0.9346919	4	2	0	1	2
66	-0.9346919	1	0	0	4	4
66	-0.9346919	1	5	3	0	0
66	-0.9346919	1	5	0	0	0
66	-0.9346919	1	0	0	0	0
66	-0.9346919	1	7	0	0	0
66	-0.9346919	1	0	0	0	0
66	-0.9346919	1	4	0	0	0
66	-0.9346919	1	9	0	0	0
66	-0.9346919	1	11	0	0	0
66	-0.9346919	1	0	0	0	0
66	-0.9346919	1	0	0	0	0
66	-0.9346919	1	0	0	0	0
67	0.28948928	1	3	0	2	0
67	0.28948928	1	0	0	0	0
67	0.28948928	1	6	0	0	0
67	0.28948928	1	9	0	0	0
67	0.28948928	1	8	0	0	0
67	0.28948928	1	0	0	0	0
67	0.28948928	1	0	0	0	0
68	-0.6331251	3	0	0	0	0
68	-0.6331251	3	0	0	6	0
68	-0.6331251	3	0	0	7	0
68	-0.6331251	3	0	0	6	0
68	-0.6331251	2	0	0	5	0
68	-0.6331251	3	0	0	6	0
68	-0.6331251	3	0	0	4	0
68	-0.6331251	3	0	0	5	0
68	-0.6331251	3	0	0	9	0
68	-0.6331251	3	0	0	0	0
68	-0.6331251	3	0	0	7	0
68	-0.6331251	3	0	0	11	0
68	-0.6331251	3	0	0	0	0
68	-0.6331251	3	0	0	6	2
68	-0.6331251	3	0	0	5	0
68	-0.6331251	3	0	0	6	0
68	-0.6331251	3	0	0	7	0
68	-0.6331251	3	0	0	2	5
68	-0.6331251	1	0	0	12	0
68	-0.6331251	3	0	0	10	0
68	-0.6331251	3	10	0	0	0
68	-0.6331251	3	0	0	7	0
68	-0.6331251	3	0	0	7	0
68	-0.6331251	1	5	0	0	3
69	-0.8855565	3	0	0	0	0
69	-0.8855565	3	0	0	0	0
69	-0.8855565	3	0	2	5	0
69	-0.8855565	3	0	0	7	0
69	-0.8855565	3	0	0	9	0
69	-0.8855565	3	0	0	7	0
69	-0.8855565	3	0	0	8	0
69	-0.8855565	3	0	0	8	0
69	-0.8855565	3	0	0	5	0
69	-0.8855565	2	0	6	0	0
69	-0.8855565	2	0	0	0	0
69	-0.8855565	2	0	0	0	0
69	-0.8855565	2	0	7	0	0
69	-0.8855565	2	0	0	0	0
69	-0.8855565	3	0	0	3	0
69	-0.8855565	3	0	0	7	0
69	-0.8855565	3	0	0	4	0
69	-0.8855565	1	6	0	2	0
69	-0.8855565	3	0	0	0	0
69	-0.8855565	3	0	0	8	0
69	-0.8855565	1	0	0	0	0
69	-0.8855565	1	0	0	10	0
69	-0.8855565	1	6	0	0	0
70	-0.1830321	2	0	0	0	0
70	-0.1830321	2	8	0	0	0
70	-0.1830321	4	0	0	0	11
70	-0.1830321	4	0	0	0	0
70	-0.1830321	4	0	0	0	0
70	-0.1830321	4	0	0	0	8
70	-0.1830321	3	0	0	0	9
70	-0.1830321	3	0	0	0	2
70	-0.1830321	4	0	0	0	5
70	-0.1830321	1	0	0	0	0
70	-0.1830321	4	0	0	0	0
70	-0.1830321	3	0	0	0	9
70	-0.1830321	4	0	0	0	0
70	-0.1830321	1	0	0	0	0
70	-0.1830321	4	0	0	0	13
70	-0.1830321	4	0	0	0	6
70	-0.1830321	4	0	0	0	7
70	-0.1830321	4	0	0	0	2
70	-0.1830321	4	0	0	0	11
70	-0.1830321	4	0	0	0	7
70	-0.1830321	4	0	0	0	0
70	-0.1830321	4	0	0	0	0
70	-0.1830321	4	0	0	0	10
70	-0.1830321	4	0	0	0	6
70	-0.1830321	4	0	0	0	6
70	-0.1830321	2	1	3	0	2
70	-0.1830321	1	6	0	0	0
70	-0.1830321	2	0	6	0	0
70	-0.1830321	2	0	7	0	0
70	-0.1830321	2	0	4	0	0
70	-0.1830321	2	2	3	0	0
70	-0.1830321	4	0	0	0	9

84	-1.0300019	1	6	0	0	0
84	-1.0300019	4	1	0	1	2
84	-1.0300019	4	0	0	0	13
84	-1.0300019	4	0	0	2	6
84	-1.0300019	4	0	0	3	4
84	-1.0300019	4	0	0	1	7
84	-1.0300019	4	1	0	3	6
84	-1.0300019	4	4	0	2	1
84	-1.0300019	4	0	0	2	9
108	-0.0297885	4	0	0	2	2
108	-0.0297885	2	2	1	1	3
108	-0.0297885	4	0	1	4	0
108	-0.0297885	4	0	0	2	4
108	-0.0297885	1	2	0	1	3
108	-0.0297885	1	2	0	1	6
108	-0.0297885	1	0	1	3	3
108	-0.0297885	4	0	0	1	3
108	-0.0297885	1	12	1	0	0
108	-0.0297885	2	0	13	0	0
108	-0.0297885	2	0	7	1	0
108	-0.0297885	3	0	0	2	0
108	-0.0297885	3	0	0	10	0
108	-0.0297885	3	0	0	6	0
108	-0.0297885	1	7	0	0	0
108	-0.0297885	3	2	0	2	1
108	-0.0297885	4	0	0	3	7
108	-0.0297885	4	0	0	1	2
108	-0.0297885	4	1	0	1	5
108	-0.0297885	4	1	0	2	2
108	-0.0297885	4	2	0	0	5
109	0.13340497	4	0	0	0	6
109	0.13340497	4	0	0	2	5
109	0.13340497	1	2	0	1	2
109	0.13340497	4	3	0	1	3
109	0.13340497	4	2	0	0	7
109	0.13340497	4	1	0	0	5
109	0.13340497	2	1	1	0	4
109	0.13340497	3	0	1	1	3
109	0.13340497	1	5	2	0	0
109	0.13340497	1	6	1	0	0
109	0.13340497	4	0	0	1	4
109	0.13340497	4	0	0	0	8
109	0.13340497	3	0	0	8	0
109	0.13340497	3	0	0	5	5
109	0.13340497	4	0	0	0	3
109	0.13340497	4	0	0	5	4
109	0.13340497	3	0	0	7	0
109	0.13340497	3	0	0	4	0
109	0.13340497	3	0	0	10	0
109	0.13340497	1	6	0	0	0
109	0.13340497	1	4	0	2	0
109	0.13340497	4	1	0	3	3
109	0.13340497	4	0	0	2	7
109	0.13340497	4	0	0	2	4
109	0.13340497	4	0	0	1	8
109	0.13340497	4	2	0	2	3
109	0.13340497	4	2	0	0	7
109	0.13340497	4	0	0	3	7
109	0.13340497	4	0	0	0	4
109	0.13340497	4	0	0	0	5
110	-0.2786646	4	0	0	0	12
110	-0.2786646	1	2	0	1	2
110	-0.2786646	1	2	1	1	3
110	-0.2786646	4	0	0	1	2
110	-0.2786646	1	5	0	1	2
110	-0.2786646	1	4	0	1	3
110	-0.2786646	4	0	0	0	8
110	-0.2786646	4	1	0	0	7
110	-0.2786646	1	3	0	0	4
110	-0.2786646	4	1	1	0	2
110	-0.2786646	4	1	3	0	1
110	-0.2786646	4	0	0	0	6
110	-0.2786646	4	0	0	0	12
110	-0.2786646	3	0	0	1	4
110	-0.2786646	3	0	0	7	0
110	-0.2786646	3	0	0	3	2
110	-0.2786646	3	6	0	1	0
110	-0.2786646	3	0	0	2	3
110	-0.2786646	4	0	0	1	6
110	-0.2786646	4	0	0	2	6
110	-0.2786646	4	0	0	1	7
110	-0.2786646	4	0	0	0	4
110	-0.2786646	3	1	0	1	4
110	-0.2786646	3	0	0	2	4
110	-0.2786646	3	0	0	1	10
110	-0.2786646	3	0	0	4	5
110	-0.2786646	4	0	0	0	3
111	-0.0589009	4	0	0	0	6
111	-0.0589009	4	0	1	1	7
111	-0.0589009	4	1	0	1	1
111	-0.0589009	4	5	0	0	3
111	-0.0589009	3	1	0	1	3
111	-0.0589009	4	1	0	0	6
111	-0.0589009	4	1	0	0	6
111	-0.0589009	1	5	0	0	3
111	-0.0589009	1	2	1	1	3
111	-0.0589009	1	4	1	0	3
111	-0.0589009	3	0	1	0	2
111	-0.0589009	4	0	2	1	3
111	-0.0589009	1	4	3	0	1
111	-0.0589009	1	5	1	0	0
111	-0.0589009	3	0	0	3	2
111	-0.0589009	3	0	0	10	0
111	-0.0589009	4	0	0	6	4
111	-0.0589009	4	0	0	2	2
111	-0.0589009	3	0	0	5	0
111	-0.0589009	4	1	0	2	3
111	-0.0589009	4	0	0	2	6
111	-0.0589009	3	0	0	2	7
111	-0.0589009	3	2	0	2	1
111	-0.0589009	4	0	0	2	6
111	-0.0589009	3	0	0	2	5
111	-0.0589009	3	0	0	0	2
111	-0.0589009	4	0	0	0	8
111	-0.0589009	4	0	0	0	7
112	-0.2892918	4	0	2	2	8
112	-0.2892918	4	0	0	3	4
112	-0.2892918	4	2	0	0	6
112	-0.2892918	1	1	0	0	7
112	-0.2892918	4	3	1	1	4
112	-0.2892918	4	3	1	0	4
112	-0.2892918	4	0	0	0	3
112	-0.2892918	4	0	1	0	5
112	-0.2892918	2	0	3	0	0
112	-0.2892918	4	0	0	0	5
112	-0.2892918	4	0	0	2	2
112	-0.2892918	4	0	0	0	8
113	-0.3337907	4	0	2	1	4
113	-0.3337907	1	2	0	2	2
113	-0.3337907	1	1	0	0	5

11	-0.776814	4	0	0	0	7
11	-0.776814	3	0	0	2	3
11	-0.776814	2	0	0	0	0
11	-0.776814	2	0	0	0	0
12	0.03251334	2	0	8	3	0
12	0.03251334	2	2	2	1	0
12	0.03251334	2	6	0	0	0
12	0.03251334	1	9	0	0	0
12	0.03251334	1	0	0	0	0
12	0.03251334	1	4	0	0	2
12	0.03251334	1	13	0	0	0
12	0.03251334	1	5	0	0	0
12	0.03251334	4	3	0	4	0
12	0.03251334	1	8	0	0	0
12	0.03251334	1	4	0	1	2
12	0.03251334	1	6	0	1	0
12	0.03251334	1	3	4	1	0
12	0.03251334	1	2	0	0	2
12	0.03251334	1	2	0	2	5
12	0.03251334	1	7	0	2	0
12	0.03251334	4	3	0	1	1
12	0.03251334	4	0	0	1	4
12	0.03251334	1	4	0	0	3
12	0.03251334	4	0	0	0	3
12	0.03251334	4	0	0	0	11
12	0.03251334	1	4	0	3	0
12	0.03251334	1	2	0	0	7
12	0.03251334	1	5	0	0	3
12	0.03251334	1	6	0	0	0
12	0.03251334	1	0	0	0	0
12	0.03251334	1	10	0	0	0
12	0.03251334	1	0	0	3	9
12	0.03251334	1	0	0	3	7
12	0.03251334	4	0	0	0	7
12	0.03251334	4	0	0	0	4
12	0.03251334	4	0	2	0	9
12	0.03251334	4	0	2	0	4
12	0.03251334	4	3	0	0	1
12	0.03251334	2	0	0	0	0
13	-0.6454721	3	0	7	3	0
13	-0.6454721	3	3	3	0	0
13	-0.6454721	3	0	9	0	0
13	-0.6454721	1	4	0	0	0
13	-0.6454721	1	9	0	1	0
13	-0.6454721	4	0	0	6	0
13	-0.6454721	4	0	0	0	4
13	-0.6454721	3	0	0	4	2
13	-0.6454721	3	4	0	2	0
13	-0.6454721	4	4	0	0	1
13	-0.6454721	4	8	0	0	0
13	-0.6454721	4	0	0	0	8
13	-0.6454721	4	3	0	0	6
13	-0.6454721	3	0	4	0	0
13	-0.6454721	4	0	0	0	5
13	-0.6454721	4	8	0	0	3
13	-0.6454721	4	0	0	1	6
13	-0.6454721	4	0	0	0	8
13	-0.6454721	4	0	0	0	8
13	-0.6454721	2	0	0	9	0
13	-0.6454721	2	0	0	6	0
13	-0.6454721	2	0	0	0	5
13	-0.6454721	4	2	0	1	4
14	0.29399424	1	8	0	0	0
14	0.29399424	2	0	4	2	0
14	0.29399424	1	8	0	0	1
14	0.29399424	1	5	0	1	2
14	0.29399424	1	2	0	0	3
14	0.29399424	4	0	0	0	0
14	0.29399424	4	0	0	0	4
14	0.29399424	4	0	0	1	5
14	0.29399424	1	0	0	0	0
14	0.29399424	4	0	0	5	0
14	0.29399424	4	0	0	2	6
14	0.29399424	4	0	0	8	0
14	0.29399424	4	0	0	0	4
14	0.29399424	4	0	0	0	8
15	0.14608837	3	0	0	0	0
15	0.14608837	4	0	0	3	5
15	0.14608837	3	0	0	0	0
15	0.14608837	3	0	0	0	0
15	0.14608837	4	0	0	4	2
15	0.14608837	3	0	0	0	2
15	0.14608837	4	0	0	0	8
15	0.14608837	4	5	0	0	0
15	0.14608837	4	0	0	0	4
15	0.14608837	4	0	0	0	9
15	0.14608837	3	0	0	11	0
15	0.14608837	3	0	0	0	0
15	0.14608837	3	0	0	9	0
15	0.14608837	1	8	0	0	0
15	0.14608837	4	0	0	5	2
15	0.14608837	3	0	0	0	9
15	0.14608837	4	0	0	6	1
15	0.14608837	3	0	0	0	9
15	0.14608837	4	0	0	6	2
15	0.14608837	4	0	0	0	7
15	0.14608837	3	0	0	7	0
15	0.14608837	4	0	0	0	0
15	0.14608837	3	0	0	0	0
15	0.14608837	4	4	0	0	0
15	0.14608837	4	0	0	0	7
15	0.14608837	4	0	0	0	6
15	0.14608837	4	0	0	0	3
15	0.14608837	1	10	0	0	0
15	0.14608837	4	0	0	0	0
15	0.14608837	1	5	0	0	0
15	0.14608837	1	0	0	0	7
15	0.14608837	3	0	0	0	7
15	0.14608837	3	0	0	0	0
15	0.14608837	4	0	0	0	8
15	0.14608837	3	0	0	0	5
15	0.14608837	3	0	0	3	0
15	0.14608837	4	0	0	6	0
15	0.14608837	4	0	0	0	0
15	0.14608837	4	0	0	0	8
15	0.14608837	4	0	0	0	0
15	0.14608837	4	0	0	0	8
15	0.14608837	3	0	0	0	0
15	0.14608837	3	0	0	6	0
15	0.14608837	3	0	0	0	0
15	0.14608837	1	0	0	0	0
15	0.14608837	4	0	0	0	0
15	0.14608837	3	0	0	11	0
15	0.14608837	4	0	0	0	0
15	0.14608837	3	0	0	3	7
15	0.14608837	1	0	0	0	0
15	0.14608837	4	5	0	0	2
15	0.14608837	3	0	0	8	0
15	0.14608837	3	0	0	4	2
15	0.14608837	3	1	0	10	0
70	-0.1830321	4	0	0	0	9
70	-0.1830321	4	0	0	0	9
70	-0.1830321	1	0	0	0	0
70	-0.1830321	4	2	0	0	2
70	-0.1830321	4	0	0	0	4
70	-0.1830321	4	0	0	0	4
70	-0.1830321	4	0	0	0	0
70	-0.1830321	4	0	0	0	11
70	-0.1830321	4	0	0	0	6
70	-0.1830321	1	0	0	0	0
70	-0.1830321	1	0	0	0	0
70	-0.1830321	4	0	0	0	9
70	-0.1830321	4	0	0	0	6
70	-0.1830321	4	0	0	0	0
70	-0.1830321	4	0	0	0	6
70	-0.1830321	4	0	0	0	6
70	-0.1830321	4	0	0	0	11
70	-0.1830321	4	0	0	0	0
70	-0.1830321	4	0	0	0	0
70	-0.1830321	4	8	0	0	0
70	-0.1830321	4	8	0	0	0
70	-0.1830321	2	4	4	0	0
70	-0.1830321	3	0	0	10	0
70	-0.1830321	4	0	0	0	10
70	-0.1830321	4	0	0	0	0
70	-0.1830321	4	0	0	0	13
70	-0.1830321	4	0	0	0	8
70	-0.1830321	4	7	0	0	0
4	0.58324324	4	0	0	0	7
4	0.58324324	4	0	0	0	0
4	0.58324324	4	0	0	0	7
4	0.58324324	4	0	0	0	0
4	0.58324324	4	0	0	2	2
4	0.58324324	4	0	0	0	0
4	0.58324324	4	0	0	0	0
4	0.58324324	4	0	0	0	0
4	0.58324324	2	0	8	0	0
4	0.58324324	2	0	5	0	0
4	0.58324324	2	0	2	0	0
4	0.58324324	4	0	0	0	0
4	0.58324324	4	0	0	0	0
4	0.58324324	4	0	0	0	0
4	0.58324324	4	3	0	0	0
4	0.58324324	4	6	0	0	0
4	0.58324324	4	2	1	0	2
4	0.58324324	4	0	0	4	6
4	0.58324324	4	0	0	0	9
4	0.58324324	4	0	0	0	3
4	0.58324324	4	0	0	0	0
4	0.58324324	1	8	0	0	0
4	0.58324324	4	0	0	0	8
4	0.58324324	4	5	0	0	0
60	0.26196807	2	2	0	0	3
60	0.26196807	1	0	0	0	0
60	0.26196807	1	0	3	0	0
60	0.26196807	2	0	6	0	0
60	0.26196807	2	8	0	0	0
60	0.26196807	2	0	6	0	0
60	0.26196807	1	0	0	0	0
60	0.26196807	1	0	0	0	3
60	0.26196807	1	0	0	0	0
60	0.26196807	1	0	0	0	0
60	0.26196807	1	0	0	0	0
60	0.26196807	1	0	0	0	5
60	0.26196807	4	0	0	0	5
60	0.26196807	4	0	0	0	8
60	0.26196807	1	3	0	0	0
60	0.26196807	1	0	0	0	0
60	0.26196807	1	0	9	0	0
60	0.26196807	4	0	0	0	0
60	0.26196807	4	0	0	0	9
60	0.26196807	1	0	0	0	0
60	0.26196807	1	0	0	0	6
60	0.26196807	1	5	4	0	0
60	0.26196807	1	0	0	0	0
60	0.26196807	3	0	0	0	7
60	0.26196807	4	0	0	0	4
60	0.26196807	1	5	0	0	1
71	-1.1600835	2	0	5	0	0
71	-1.1600835	2	3	0	0	0
71	-1.1600835	1	9	0	0	0
71	-1.1600835	2	0	0	0	0
71	-1.1600835	2	0	4	0	0
71	-1.1600835	2	0	4	0	0
71	-1.1600835	2	6	0	0	0
71	-1.1600835	4	0	0	0	7
71	-1.1600835	4	3	0	0	2
71	-1.1600835	1	1	0	0	6
71	-1.1600835	4	0	0	0	8
71	-1.1600835	4	0	0	0	5
71	-1.1600835	4	0	0	0	9
71	-1.1600835	4	0	0	0	0
71	-1.1600835	1	8	0	0	0
71	-1.1600835	4	0	0	2	5
71	-1.1600835	4	0	0	0	10
71	-1.1600835	4	0	0	0	12
71	-1.1600835	4	0	0	0	0
71	-1.1600835	2	4	3	0	0
71	-1.1600835	2	1	2	0	0
71	-1.1600835	4	0	0	0	0
71	-1.1600835	4	0	0	0	5
71	-1.1600835	4	0	0	0	5
71	-1.1600835	1	4	0	0	2
72	0.02524535	1	0	0	8	0
72	0.02524535	1	4	0	1	0
72	0.02524535	4	4	0	4	0
72	0.02524535	4	0	0	5	2
72	0.02524535	4	1	0	5	3
72	0.02524535	1	12	0	0	0
72	0.02524535	4	0	0	3	1
72	0.02524535	4	4	0	0	4
72	0.02524535	4	5	0	0	3
72	0.02524535	1	4	0	1	6
72	0.02524535	1	10	0	0	0
72	0.02524535	1	8	0	0	0
72	0.02524535	1	4	0	2	0
72	0.02524535	1	6	0	1	1
72	0.02524535	4	0	0	2	3
72	0.02524535	1	0	0	0	9
72	0.02524535	1	4	0	0	1
72	0.02524535	1	3	0	0	2
72	0.02524535	4	2	0	0	2
72	0.02524535	1	6	0	0	2
72	0.02524535	1	5	0	3	0
72	0.02524535	1	1	0	0	2
72	0.02524535	1	4	0	0	0
72	0.02524535	3	2	0	7	0
72	0.02524535	3	3	0	1	0
72	0.02524535	3	0	0	9	0
72	0.02524535	3	0	0	3	0
113	-0.3337907	3	0	0	5	0
113	-0.3337907	1	7	0	0	0
113	-0.3337907	1	2	0	1	2
114	-0.4855974	4	0	1	1	6
114	-0.4855974	4	0	0	2	5
114	-0.4855974	3	1	0	1	4
114	-0.4855974	4	0	0	2	5
114	-0.4855974	4	1	0	0	6
114	-0.4855974	4	0	0	0	10
114	-0.4855974	4	0	1	1	6
114	-0.4855974	2	0	6	1	0
114	-0.4855974	4	0	0	0	9
114	-0.4855974	4	0	0	0	8
114	-0.4855974	3	0	0	2	8
114	-0.4855974	4	0	0	0	12
114	-0.4855974	3	0	0	2	5
114	-0.4855974	4	0	0	0	5
114	-0.4855974	4	0	0	3	3
114	-0.4855974	4	0	0	4	4
114	-0.4855974	4	0	0	1	3
114	-0.4855974	4	0	0	0	5
114	-0.4855974	4	0	0	2	8
114	-0.4855974	4	2	0	3	4
114	-0.4855974	1	2	0	2	0
114	-0.4855974	4	0	0	0	11
115	-0.6635707	4	0	1	1	5
115	-0.6635707	2	1	4	0	2
115	-0.6635707	4	3	0	2	2
115	-0.6635707	4	0	0	0	7
115	-0.6635707	4	1	1	0	1
115	-0.6635707	4	0	0	0	3
115	-0.6635707	3	0	0	1	3
115	-0.6635707	4	0	0	2	2
115	-0.6635707	3	0	0	0	5
58	0.44441689	4	0	1	1	6
58	0.44441689	4	0	1	2	2
58	0.44441689	4	0	0	1	2
58	0.44441689	1	1	0	1	4
58	0.44441689	1	2	0	0	4
58	0.44441689	4	3	0	1	2
58	0.44441689	4	0	0	0	6
58	0.44441689	4	1	0	0	5
58	0.44441689	4	1	3	0	1
58	0.44441689	4	1	0	1	4
58	0.44441689	4	0	0	2	6
58	0.44441689	4	0	0	2	10
58	0.44441689	3	1	0	1	4
58	0.44441689	4	0	0	1	12
58	0.44441689	4	0	0	0	4
58	0.44441689	3	0	0	1	9
58	0.44441689	4	0	0	0	11
58	0.44441689	4	0	0	0	7
58	0.44441689	4	0	0	0	9
116	-0.3189659	4	0	0	2	6
116	-0.3189659	4	0	0	2	9
116	-0.3189659	4	0	2	2	3
116	-0.3189659	4	0	0	0	8
116	-0.3189659	4	0	0	0	0
116	-0.3189659	4	0	0	0	4
116	-0.3189659	4	0	0	4	1
116	-0.3189659	4	0	0	6	1
116	-0.3189659	3	0	0	3	0
116	-0.3189659	4	1	0	2	8
116	-0.3189659	4	0	0	2	5
116	-0.3189659	4	0	0	1	5
116	-0.3189659	4	0	0	3	7
116	-0.3189659	4	0	0	1	5
116	-0.3189659	4	1	0	1	6
116	-0.3189659	4	0	0	0	7
117	-0.4636981	4	0	0	1	6
117	-0.4636981	4	1	0	2	5
117	-0.4636981	4	3	0	1	5
117	-0.4636981	4	2	0	0	5
117	-0.4636981	4	2	0	0	5
117	-0.4636981	4	0	4	0	2
117	-0.4636981	2	0	3	1	4
117	-0.4636981	4	0	0	2	5
117	-0.4636981	4	0	0	0	12
117	-0.4636981	4	0	0	1	6
117	-0.4636981	4	0	0	2	1
117	-0.4636981	4	0	0	2	6
117	-0.4636981	3	0	0	8	0
117	-0.4636981	3	1	0	1	4
117	-0.4636981	3	1	0	4	2
117	-0.4636981	4	0	0	3	5
117	-0.4636981	4	0	0	1	6
117	-0.4636981	3	1	0	1	3
117	-0.4636981	4	0	0	1	8
117	-0.4636981	4	0	0	0	2
118	-0.0647231	4	1	0	0	4
118	-0.0647231	4	0	0	3	4
118	-0.0647231	4	0	3	1	3
118	-0.0647231	2	0	5	0	0
118	-0.0647231	1	3	0	0	0
118	-0.0647231	3	0	0	2	6
119	0.06088944	1	1	0	1	4
119	0.06088944	4	2	0	1	4
119	0.06088944	4	1	0	0	7
119	0.06088944	1	2	0	0	6
119	0.06088944	4	1	1	1	3
119	0.06088944	4	0	0	2	4
119	0.06088944	4	0	0	1	9
119	0.06088944	1	0	1	1	5
119	0.06088944	2	6	3	0	0
119	0.06088944	2	6	0	0	0
119	0.06088944	1	0	0	0	3
119	0.06088944	3	0	6	1	0
119	0.06088944	3	0	0	2	3
119	0.06088944	3	0	0	1	1
119	0.06088944	4	0	0	2	5
119	0.06088944	3	1	0	1	5
119	0.06088944	4	0	0	0	5
120	0.26063918	3	1	0	1	4
120	0.26063918	4	1	0	0	4
120	0.26063918	3	5	1	0	4
120	0.26063918	2	3	0	0	3
120	0.26063918	2	3	1	0	2
120	0.26063918	3	0	0	0	0
120	0.26063918	3	0	0	1	2
120	0.26063918	1	5	1	0	0
120	0.26063918	3	0	1	0	8
120	0.26063918	3	0	0	0	3
120	0.26063918	3	0	0	0	13
120	0.26063918	3	0	0	5	0
120	0.26063918	3	0	0	3	2
120	0.26063918	3	0	0	1	3
120	0.26063918	3	0	0	10	0
120	0.26063918	3	8	0	0	0
120	0.26063918	3	4	0	0	0
120	0.26063918	3	1	0	3	3

15	0.14608837	3	0	0	5	0
15	0.14608837	3	0	0	3	0
15	0.14608837	3	0	0	4	0
15	0.14608837	1	1	0	7	0
15	0.14608837	3	9	0	3	0
15	0.14608837	1	6	0	0	0
15	0.14608837	3	0	0	0	9
15	0.14608837	3	0	0	4	0
15	0.14608837	3	0	0	4	0
15	0.14608837	3	0	0	10	0
15	0.14608837	3	0	0	5	0
15	0.14608837	3	0	0	4	0
15	0.14608837	3	0	0	7	0
15	0.14608837	3	0	0	0	0
15	0.14608837	1	0	0	0	0
15	0.14608837	3	0	0	0	0
15	0.14608837	3	0	0	11	0
15	0.14608837	3	0	0	10	0
15	0.14608837	3	0	0	7	0
15	0.14608837	3	0	0	8	0
15	0.14608837	1	0	0	10	0
15	0.14608837	3	0	0	0	0
15	0.14608837	3	0	0	0	0
15	0.14608837	1	0	0	11	0
15	0.14608837	3	0	0	6	0
15	0.14608837	3	0	0	5	0
15	0.14608837	3	0	0	6	0
15	0.14608837	3	0	0	0	0
15	0.14608837	3	0	0	0	0
15	0.14608837	3	0	0	6	0
15	0.14608837	3	6	0	0	0
15	0.14608837	1	5	0	0	0
15	0.14608837	1	11	0	0	0
15	0.14608837	1	4	0	1	0
15	0.14608837	1	8	0	0	0
15	0.14608837	1	6	0	0	0
15	0.14608837	1	12	0	0	0
15	0.14608837	1	7	0	0	0
15	0.14608837	1	10	0	0	0
15	0.14608837	1	0	0	0	0
15	0.14608837	1	0	0	0	0
15	0.14608837	1	0	0	0	0
15	0.14608837	1	11	0	0	0
15	0.14608837	1	0	0	0	0
15	0.14608837	1	0	0	0	0
16	0.26298722	4	0	0	0	0
16	0.26298722	4	0	0	7	0
16	0.26298722	4	0	0	0	9
16	0.26298722	3	0	0	6	0
16	0.26298722	3	0	0	12	0
16	0.26298722	3	0	0	2	5
16	0.26298722	4	0	0	0	9
16	0.26298722	4	0	0	6	3
16	0.26298722	4	0	0	0	9
16	0.26298722	4	0	0	0	8
16	0.26298722	4	0	0	0	0
16	0.26298722	4	0	0	7	0
16	0.26298722	4	0	0	3	5
16	0.26298722	4	0	0	9	0
16	0.26298722	1	6	0	0	0
16	0.26298722	1	7	0	0	0
17	-0.5629747	4	0	0	4	8
17	-0.5629747	4	0	0	0	0
17	-0.5629747	4	4	0	0	4
17	-0.5629747	4	0	0	0	4
17	-0.5629747	4	0	0	0	0
17	-0.5629747	4	0	0	0	0
17	-0.5629747	4	0	0	8	0
17	-0.5629747	4	0	0	1	8
17	-0.5629747	4	0	0	12	0
17	-0.5629747	4	0	0	0	0
17	-0.5629747	4	0	0	0	0
17	-0.5629747	4	0	0	2	2
17	-0.5629747	4	2	0	2	3
17	-0.5629747	4	0	0	2	5
17	-0.5629747	3	0	0	5	0
17	-0.5629747	3	0	0	7	0
17	-0.5629747	3	2	0	4	0
17	-0.5629747	3	0	0	9	0
17	-0.5629747	3	0	0	4	0
17	-0.5629747	1	8	0	0	0
17	-0.5629747	1	5	0	2	0
17	-0.5629747	1	7	0	0	0
17	-0.5629747	1	6	0	0	0
18	-0.4790092	4	0	0	2	2
18	-0.4790092	4	0	0	0	0
18	-0.4790092	3	0	0	4	0
18	-0.4790092	4	0	0	0	0
18	-0.4790092	4	0	0	7	0
18	-0.4790092	4	0	0	3	2
18	-0.4790092	4	0	0	10	0
18	-0.4790092	4	0	0	6	2
18	-0.4790092	4	0	0	5	0
18	-0.4790092	4	0	0	0	11
18	-0.4790092	4	0	0	12	0
18	-0.4790092	4	6	0	0	0
18	-0.4790092	4	0	0	0	0
18	-0.4790092	4	4	0	0	0
18	-0.4790092	4	0	0	0	6
18	-0.4790092	4	0	0	2	3
18	-0.4790092	4	4	0	0	4
18	-0.4790092	4	0	0	0	3
18	-0.4790092	4	0	0	9	0
18	-0.4790092	3	0	0	4	4
18	-0.4790092	4	0	0	7	0
18	-0.4790092	4	0	0	0	6
18	-0.4790092	4	0	0	0	11
18	-0.4790092	4	0	0	0	0
18	-0.4790092	4	4	0	5	0
18	-0.4790092	4	0	0	0	7
18	-0.4790092	4	0	0	7	0
18	-0.4790092	3	0	0	4	0
18	-0.4790092	3	0	0	4	0
18	-0.4790092	3	0	0	0	0
18	-0.4790092	3	1	0	4	0
18	-0.4790092	1	4	0	3	0
18	-0.4790092	3	4	0	2	0
18	-0.4790092	3	3	0	6	0
18	-0.4790092	3	0	0	5	0
18	-0.4790092	3	0	0	12	0
18	-0.4790092	3	0	0	9	0
18	-0.4790092	3	0	0	12	0
18	-0.4790092	3	0	0	8	0
18	-0.4790092	3	0	0	8	0
18	-0.4790092	3	0	0	8	0
18	-0.4790092	3	0	0	6	0
18	-0.4790092	3	0	0	9	0
18	-0.4790092	3	0	0	7	0
18	-0.4790092	3	6	0	0	0
18	-0.4790092	1	10	0	0	0

72	0.02524535	1	8	0	0	0
72	0.02524535	1	5	0	0	0
72	0.02524535	4	5	0	0	0
73	-1.1141344	3	3	0	0	0
73	-1.1141344	3	0	0	0	4
73	-1.1141344	1	0	0	0	0
73	-1.1141344	1	3	0	0	0
73	-1.1141344	1	8	0	0	0
73	-1.1141344	1	6	0	0	0
73	-1.1141344	3	0	0	4	2
73	-1.1141344	1	9	0	1	0
73	-1.1141344	1	6	0	0	1
73	-1.1141344	1	10	0	0	1
73	-1.1141344	1	2	0	0	2
73	-1.1141344	1	5	0	0	1
73	-1.1141344	1	5	0	1	0
73	-1.1141344	1	2	0	2	0
73	-1.1141344	1	10	0	0	0
73	-1.1141344	1	8	0	0	0
73	-1.1141344	1	10	0	0	0
73	-1.1141344	1	6	0	0	0
73	-1.1141344	1	4	0	0	0
40	-0.9621317	3	0	0	2	2
40	-0.9621317	4	0	0	4	2
40	-0.9621317	1	6	0	0	0
40	-0.9621317	1	7	0	0	0
40	-0.9621317	1	6	0	0	0
40	-0.9621317	1	5	0	0	1
40	-0.9621317	4	0	0	4	2
40	-0.9621317	1	4	0	0	4
40	-0.9621317	1	8	0	0	0
40	-0.9621317	1	2	0	2	0
40	-0.9621317	1	5	0	0	0
40	-0.9621317	1	7	0	1	0
40	-0.9621317	1	7	0	0	2
40	-0.9621317	1	1	0	0	3
40	-0.9621317	1	3	0	0	3
40	-0.9621317	1	8	0	0	0
40	-0.9621317	1	6	0	0	3
40	-0.9621317	1	3	0	0	0
40	-0.9621317	3	4	0	4	0
40	-0.9621317	1	5	0	0	0
40	-0.9621317	3	0	0	0	0
40	-0.9621317	3	0	0	4	0
40	-0.9621317	3	0	0	5	0
40	-0.9621317	3	0	0	7	0
40	-0.9621317	1	2	0	4	0
40	-0.9621317	1	9	0	0	0
40	-0.9621317	1	6	0	0	2
40	-0.9621317	1	10	0	0	0
40	-0.9621317	1	3	0	0	4
35	0.08994369	1	0	0	1	4
35	0.08994369	1	3	0	0	4
35	0.08994369	1	1	0	3	2
35	0.08994369	1	11	0	0	0
35	0.08994369	1	6	0	0	0
35	0.08994369	1	3	0	0	2
35	0.08994369	4	0	0	2	2
35	0.08994369	1	0	0	0	3
35	0.08994369	4	0	0	4	0
35	0.08994369	1	5	0	0	2
35	0.08994369	1	4	0	3	0
35	0.08994369	1	6	0	2	0
35	0.08994369	1	5	0	2	0
35	0.08994369	1	7	0	2	0
35	0.08994369	1	4	0	1	2
35	0.08994369	4	1	0	0	5
35	0.08994369	4	4	0	0	4
35	0.08994369	1	4	0	0	4
35	0.08994369	1	7	0	0	1
35	0.08994369	1	4	0	0	2
35	0.08994369	1	5	0	1	2
35	0.08994369	1	8	0	0	0
35	0.08994369	4	0	0	2	5
35	0.08994369	3	0	0	10	0
35	0.08994369	3	0	0	2	0
35	0.08994369	3	0	0	5	0
35	0.08994369	1	2	0	0	5
35	0.08994369	1	7	0	0	2
32	-0.8084256	3	0	0	2	4
32	-0.8084256	3	2	0	2	0
32	-0.8084256	3	1	0	2	2
32	-0.8084256	1	9	0	0	0
32	-0.8084256	1	5	0	0	0
32	-0.8084256	3	2	0	1	4
32	-0.8084256	3	0	0	0	5
32	-0.8084256	3	5	0	2	0
32	-0.8084256	3	1	0	3	0
32	-0.8084256	1	8	0	0	0
32	-0.8084256	3	3	0	8	0
32	-0.8084256	4	2	0	1	6
32	-0.8084256	4	0	0	0	5
32	-0.8084256	3	1	0	0	6
32	-0.8084256	1	2	0	0	4
32	-0.8084256	1	4	0	0	0
32	-0.8084256	4	7	0	0	1
32	-0.8084256	4	3	0	0	6
32	-0.8084256	3	5	0	1	3
32	-0.8084256	3	0	0	0	0
32	-0.8084256	3	0	0	7	3
32	-0.8084256	3	2	0	0	4
32	-0.8084256	3	5	0	5	0
32	-0.8084256	1	12	0	0	0
32	-0.8084256	3	0	0	6	0
32	-0.8084256	3	0	0	4	0
32	-0.8084256	3	0	0	6	0
32	-0.8084256	1	8	0	0	1
32	-0.8084256	1	6	0	0	0
31	0.27911465	3	0	0	5	2
31	0.27911465	3	0	0	0	5
31	0.27911465	3	0	0	3	3
31	0.27911465	3	0	0	4	2
31	0.27911465	3	0	0	1	10
31	0.27911465	3	0	0	4	3
31	0.27911465	3	0	0	2	4
31	0.27911465	3	4	0	0	5
31	0.27911465	3	5	0	2	0
31	0.27911465	3	10	0	1	0
31	0.27911465	3	2	0	4	2
31	0.27911465	3	0	0	5	0
31	0.27911465	3	0	0	0	4
31	0.27911465	4	4	0	0	3
31	0.27911465	4	0	0	0	7
31	0.27911465	4	2	0	0	5
31	0.27911465	4	6	0	0	2
31	0.27911465	4	0	0	0	10
31	0.27911465	4	0	0	0	5
31	0.27911465	4	4	0	0	2
31	0.27911465	4	4	0	0	0
31	0.27911465	3	5	0	0	0

120	0.26063918	4	0	0	1	5
120	0.26063918	4	0	0	0	0
120	0.26063918	4	0	0	1	5
120	0.26063918	4	0	0	0	9
120	0.26063918	3	0	0	1	5
120	0.26063918	3	0	0	0	4
120	0.26063918	3	1	0	0	4
120	0.26063918	3	0	0	1	6
120	0.26063918	4	0	0	0	12
121	-0.7947767	1	1	0	0	3
121	-0.7947767	4	3	0	0	3
121	-0.7947767	4	4	0	2	2
121	-0.7947767	4	3	1	0	2
121	-0.7947767	2	1	1	1	5
121	-0.7947767	2	2	1	0	0
121	-0.7947767	2	1	1	1	4
121	-0.7947767	2	0	7	3	0
121	-0.7947767	4	1	0	2	4
121	-0.7947767	3	0	0	1	6
121	-0.7947767	3	0	0	1	7
121	-0.7947767	3	0	0	1	3
121	-0.7947767	3	0	0	1	6
121	-0.7947767	4	0	0	0	0
121	-0.7947767	4	0	0	0	11
122	-0.1521684	4	1	0	1	3
122	-0.1521684	1	2	0	2	5
122	-0.1521684	4	1	0	0	7
122	-0.1521684	4	1	0	0	6
122	-0.1521684	4	2	0	0	2
122	-0.1521684	4	2	1	1	3
122	-0.1521684	4	1	3	0	5
122	-0.1521684	4	0	0	2	6
122	-0.1521684	3	0	6	1	0
122	-0.1521684	3	0	0	3	2
122	-0.1521684	4	0	0	0	7
122	-0.1521684	4	0	0	2	5
122	-0.1521684	4	0	0	1	3
122	-0.1521684	1	8	0	1	0
122	-0.1521684	4	0	0	3	6
122	-0.1521684	4	0	0	2	4
122	-0.1521684	4	0	0	0	4
122	-0.1521684	4	0	0	0	5
123	0.10404049	4	0	1	4	5
123	0.10404049	4	0	0	1	9
123	0.10404049	4	4	0	1	3
123	0.10404049	4	0	0	0	9
123	0.10404049	4	2	2	2	2
123	0.10404049	4	1	2	1	2
123	0.10404049	3	0	0	8	0
123	0.10404049	4	0	0	0	4
123	0.10404049	4	0	0	1	8
123	0.10404049	4	0	0	1	6
123	0.10404049	4	0	0	1	6
123	0.10404049	4	0	0	0	10
61	-0.2053642	4	0	0	2	6
61	-0.2053642	4	0	0	0	4
61	-0.2053642	4	0	0	4	6
61	-0.2053642	4	0	0	2	7
61	-0.2053642	4	0	0	2	6
61	-0.2053642	4	0	0	1	7
61	-0.2053642	4	0	0	1	5
61	-0.2053642	4	0	0	1	5
61	-0.2053642	3	0	0	1	8
61	-0.2053642	4	0	0	0	3
61	-0.2053642	4	0	0	0	0
124	-0.1525137	4	0	0	1	4
124	-0.1525137	4	2	1	1	6
124	-0.1525137	4	4	4	0	1
124	-0.1525137	1	10	0	0	0
124	-0.1525137	1	3	0	1	0
57	0.46721311	4	1	0	1	4
57	0.46721311	4	0	1	0	2
57	0.46721311	3	0	0	9	0
57	0.46721311	3	0	0	5	3
57	0.46721311	1	4	0	0	0
57	0.46721311	4	4	0	2	0
57	0.46721311	4	0	0	1	9
57	0.46721311	4	1	0	3	6
125	-1.0960776	4	4	0	1	5
125	-1.0960776	2	2	1	1	3
125	-1.0960776	2	1	3	0	2
125	-1.0960776	4	0	0	1	2
125	-1.0960776	4	0	0	0	5
125	-1.0960776	4	0	0	5	1
125	-1.0960776	4	0	0	2	4
125	-1.0960776	4	0	0	1	6
125	-1.0960776	4	1	0	1	5
125	-1.0960776	4	0	0	0	9
125	-1.0960776	4	0	0	0	9
125	-1.0960776	4	0	0	0	10
125	-1.0960776	4	0	0	0	6
126	-0.5074377	4	1	0	0	8
126	-0.5074377	2	1	2	0	2
126	-0.5074377	4	0	0	0	7
127	0.21650526	4	1	0	0	4
127	0.21650526	4	2	0	0	0
127	0.21650526	4	3	0	0	4
127	0.21650526	1	3	2	0	4
127	0.21650526	1	1	1	0	2
127	0.21650526	4	3	1	0	1
127	0.21650526	3	0	0	4	2
127	0.21650526	3	0	0	5	0
127	0.21650526	4	0	0	7	1
127	0.21650526	3	0	0	8	0
127	0.21650526	1	9	0	0	0
127	0.21650526	1	1	0	2	4
127	0.21650526	1	1	0	3	5
62	-0.8150679	4	2	0	0	7
62	-0.8150679	3	0	1	2	6
62	-0.8150679	4	0	0	0	5
62	-0.8150679	3	0	0	5	0
62	-0.8150679	4	0	0	1	5
62	-0.8150679	3	0	0	9	0
62	-0.8150679	4	0	0	1	8
62	-0.8150679	4	0	0	2	10
62	-0.8150679	4	0	0	1	4
62	-0.8150679	3	0	0	1	5
62	-0.8150679	1	1	0	2	3
62	-0.8150679	4	0	0	1	5
128	0.60853009	2	0	3	2	0
128	0.60853009	2	0	8	2	0
128	0.60853009	4	0	0	1	8
128	0.60853009	4	0	0	0	0
128	0.60853009	4	0	0	0	6
128	0.60853009	4	0	0	0	9
128	0.60853009	4	0	0	1	2
128	0.60853009	3	0	0	1	5
128	0.60853009	3	0	0	4	2
128	0.60853009	4	0	0	0	6
128	0.60853009	4	1	0	2	3

18	-0.4790092	1	5	0	0	0
18	-0.4790092	1	0	0	2	5
18	-0.4790092	1	2	0	3	0
18	-0.4790092	1	4	0	0	0
18	-0.4790092	1	9	0	1	0
18	-0.4790092	1	0	0	0	0
19	0.1133087	1	2	0	5	0
19	0.1133087	1	0	0	0	0
19	0.1133087	4	7	0	0	0
19	0.1133087	1	10	0	0	0
19	0.1133087	4	0	0	7	0
19	0.1133087	3	0	0	6	0
19	0.1133087	3	0	0	11	0
19	0.1133087	3	0	0	4	0
19	0.1133087	3	0	0	6	0
19	0.1133087	1	4	0	2	0
19	0.1133087	3	3	0	3	0
19	0.1133087	3	0	0	8	0
19	0.1133087	3	0	0	6	0
19	0.1133087	3	0	0	3	0
19	0.1133087	3	4	0	4	0
19	0.1133087	3	3	0	8	0
19	0.1133087	3	0	0	4	0
19	0.1133087	3	8	0	0	0
19	0.1133087	3	1	0	6	0
19	0.1133087	1	0	0	0	0
6	0.04574931	1	7	0	0	0
6	0.04574931	1	2	0	2	0
20	0.26275188	3	0	0	0	0
21	-0.5652193	1	0	0	11	0
21	-0.5652193	2	0	10	0	0
21	-0.5652193	1	2	0	2	3
21	-0.5652193	1	0	0	3	2
21	-0.5652193	3	0	8	0	0
21	-0.5652193	2	0	4	0	4
21	-0.5652193	1	0	6	0	0
21	-0.5652193	1	0	3	2	1
21	-0.5652193	2	0	6	0	0
21	-0.5652193	2	0	2	4	0
21	-0.5652193	1	2	4	0	0
21	-0.5652193	1	7	0	0	3
21	-0.5652193	1	1	6	0	1
21	-0.5652193	1	0	2	0	4
22	0.01992373	4	0	8	0	0
22	0.01992373	4	0	0	0	0
22	0.01992373	4	3	4	0	0
22	0.01992373	4	4	0	0	0
22	0.01992373	4	2	0	4	0
22	0.01992373	4	0	0	0	0
22	0.01992373	1	0	4	0	0
22	0.01992373	4	0	11	0	0
22	0.01992373	4	0	0	0	0
22	0.01992373	1	10	0	0	0
22	0.01992373	4	0	0	0	7
22	0.01992373	4	0	2	4	3
22	0.01992373	2	0	0	8	2
22	0.01992373	4	0	0	5	0
22	0.01992373	4	4	1	0	3
22	0.01992373	4	0	0	3	2
22	0.01992373	4	0	0	4	7
22	0.01992373	4	0	0	2	3
22	0.01992373	4	0	0	3	5
22	0.01992373	4	0	0	4	4
22	0.01992373	4	0	0	2	1
22	0.01992373	4	0	0	4	0
22	0.01992373	4	0	5	1	1
22	0.01992373	4	0	8	0	2
22	0.01992373	4	0	4	0	0
22	0.01992373	4	0	7	0	0
22	0.01992373	4	0	0	0	0
22	0.01992373	4	0	5	3	0
22	0.01992373	4	0	5	0	0
22	0.01992373	4	0	0	0	8
22	0.01992373	4	0	1	0	9
22	0.01992373	4	0	0	0	5
22	0.01992373	4	0	3	0	3
22	0.01992373	4	0	2	2	2
22	0.01992373	4	0	0	0	7
22	0.01992373	4	0	6	0	4
22	0.01992373	4	0	7	0	0
22	0.01992373	4	0	4	0	2
22	0.01992373	1	8	3	0	0
22	0.01992373	4	4	1	0	2
22	0.01992373	4	2	4	0	2
22	0.01992373	4	2	0	0	3
22	0.01992373	4	0	0	0	7
22	0.01992373	4	0	0	0	10
22	0.01992373	4	0	0	0	8
22	0.01992373	4	1	0	0	7
22	0.01992373	1	2	0	0	3
23	-0.918747	2	0	0	0	12
23	-0.918747	2	3	0	0	4
23	-0.918747	2	4	0	0	0
23	-0.918747	2	0	0	0	0
23	-0.918747	2	5	0	0	0
23	-0.918747	2	0	7	0	0
23	-0.918747	2	0	6	0	0
23	-0.918747	3	7	0	0	0
23	-0.918747	3	4	0	0	4
23	-0.918747	3	0	0	1	3
23	-0.918747	3	0	0	0	0
23	-0.918747	2	0	4	0	2
23	-0.918747	2	0	7	0	0
23	-0.918747	2	0	7	0	0
23	-0.918747	2	0	3	0	3
23	-0.918747	2	0	0	0	7
23	-0.918747	2	0	1	0	7
23	-0.918747	2	0	0	0	6
23	-0.918747	2	0	0	0	7
23	-0.918747	2	0	3	4	1
23	-0.918747	2	0	2	0	6
23	-0.918747	2	0	4	0	3
23	-0.918747	2	2	3	0	0
23	-0.918747	2	2	4	0	3
23	-0.918747	2	4	3	0	2
23	-0.918747	2	2	0	0	4
23	-0.918747	4	6	1	0	1
23	-0.918747	4	2	4	0	2
23	-0.918747	4	4	0	0	4
23	-0.918747	4	0	0	0	7
23	-0.918747	4	0	1	2	6
23	-0.918747	2	0	0	0	10
23	-0.918747	1	3	0	0	5
23	-0.918747	1	0	4	0	6
24	0.13049266	2	0	0	0	6
24	0.13049266	2	0	9	0	0
24	0.13049266	2	1	0	2	2
24	0.13049266	3	0	0	0	0
24	0.13049266	2	0	9	0	0
24	0.13049266	2	0	5	0	0
31	0.27911465	3	12	0	0	0
31	0.27911465	3	0	0	4	4
31	0.27911465	3	0	0	9	0
31	0.27911465	3	0	0	7	0
31	0.27911465	4	0	0	0	0
36	-0.6623215	1	0	0	9	0
36	-0.6623215	1	8	0	0	0
36	-0.6623215	1	1	0	0	4
36	-0.6623215	1	8	0	0	4
36	-0.6623215	1	3	0	3	0
36	-0.6623215	1	1	0	0	1
36	-0.6623215	1	3	0	0	2
36	-0.6623215	1	6	0	0	0
29	-0.4265799	3	0	0	4	3
29	-0.4265799	3	3	0	1	4
29	-0.4265799	1	10	0	0	0
29	-0.4265799	1	4	0	0	3
29	-0.4265799	3	0	0	9	0
29	-0.4265799	4	0	0	0	8
29	-0.4265799	3	2	0	0	5
29	-0.4265799	4	8	0	0	1
29	-0.4265799	1	6	0	0	2
29	-0.4265799	4	3	0	0	1
29	-0.4265799	4	0	0	0	9
29	-0.4265799	4	5	0	0	2
29	-0.4265799	1	5	0	0	0
29	-0.4265799	3	0	0	4	3
29	-0.4265799	3	0	0	8	0
29	-0.4265799	3	0	0	4	0
29	-0.4265799	1	3	0	0	0
46	-0.5263299	1	0	0	3	2
46	-0.5263299	1	7	0	0	0
46	-0.5263299	1	9	0	0	1
46	-0.5263299	1	2	0	0	5
46	-0.5263299	1	7	0	0	0
46	-0.5263299	1	2	0	4	0
46	-0.5263299	1	7	0	1	0
46	-0.5263299	4	3	0	1	3
46	-0.5263299	4	0	0	1	4
46	-0.5263299	4	3	0	0	4
46	-0.5263299	1	3	0	0	3
46	-0.5263299	4	10	0	0	1
46	-0.5263299	1	7	0	0	0
46	-0.5263299	1	5	0	0	4
46	-0.5263299	1	5	0	0	2
46	-0.5263299	1	9	0	1	1
46	-0.5263299	1	6	0	0	0
46	-0.5263299	1	5	0	0	0
46	-0.5263299	4	0	0	4	3
46	-0.5263299	3	0	0	6	0
46	-0.5263299	4	6	0	0	2
46	-0.5263299	4	4	0	0	4
46	-0.5263299	4	0	0	0	0
46	-0.5263299	1	7	0	0	0
46	-0.5263299	1	6	0	0	0
46	-0.5263299	4	0	0	0	9
74	-0.0456928	4	2	0	2	1
74	-0.0456928	1	6	0	0	0
74	-0.0456928	1	11	0	0	0
74	-0.0456928	1	2	0	0	0
74	-0.0456928	4	5	0	0	3
74	-0.0456928	4	0	0	0	5
74	-0.0456928	4	1	0	2	3
74	-0.0456928	4	4	0	0	4
74	-0.0456928	1	4	0	1	0
74	-0.0456928	3	0	0	4	0
74	-0.0456928	4	0	0	0	10
74	-0.0456928	4	0	0	0	0
74	-0.0456928	4	2	0	0	4
74	-0.0456928	4	3	0	0	5
74	-0.0456928	4	4	0	1	1
74	-0.0456928	4	8	0	0	3
74	-0.0456928	4	0	0	0	0
74	-0.0456928	4	8	0	0	1
74	-0.0456928	4	3	0	1	3
74	-0.0456928	4	3	0	1	2
74	-0.0456928	3	2	0	0	4
74	-0.0456928	3	0	0	9	0
74	-0.0456928	3	0	0	7	0
74	-0.0456928	3	2	0	5	0
74	-0.0456928	4	0	0	9	2
74	-0.0456928	3	0	0	6	0
74	-0.0456928	3	0	0	8	0
74	-0.0456928	3	0	0	8	0
74	-0.0456928	3	0	0	6	0
74	-0.0456928	3	0	0	8	0
74	-0.0456928	4	0	0	0	0
74	-0.0456928	4	9	0	0	3
74	-0.0456928	1	9	0	0	0
74	-0.0456928	4	4	0	0	3
44	-0.8338155	1	4	0	0	0
44	-0.8338155	1	6	0	0	0
44	-0.8338155	1	5	0	0	0
44	-0.8338155	1	6	0	0	1
44	-0.8338155	4	4	0	0	1
44	-0.8338155	3	0	0	0	3
44	-0.8338155	3	0	0	2	5
44	-0.8338155	4	0	0	3	3
44	-0.8338155	3	1	0	1	3
44	-0.8338155	3	0	0	2	2
44	-0.8338155	3	0	0	6	2
44	-0.8338155	4	0	0	4	2
44	-0.8338155	4	0	0	0	8
44	-0.8338155	1	1	0	0	2
44	-0.8338155	1	9	0	1	0
44	-0.8338155	1	3	0	0	0
44	-0.8338155	1	7	0	1	0
44	-0.8338155	3	6	0	0	0
44	-0.8338155	3	2	0	4	0
44	-0.8338155	4	2	0	3	1
44	-0.8338155	4	3	0	2	2
44	-0.8338155	4	0	0	0	5
44	-0.8338155	4	1	0	1	3
44	-0.8338155	4	6	0	0	2
44	-0.8338155	1	2	0	0	5
44	-0.8338155	4	8	0	0	2
44	-0.8338155	3	4	0	0	4
44	-0.8338155	3	4	0	0	2
44	-0.8338155	4	5	0	0	5
44	-0.8338155	4	10	0	0	0
44	-0.8338155	4	7	0	0	0
44	-0.8338155	1	9	0	0	0
44	-0.8338155	3	7	0	0	0
44	-0.8338155	3	2	0	3	0
44	-0.8338155	3	0	0	4	4
44	-0.8338155	3	11	0	0	0
44	-0.8338155	3	2	0	3	0
44	-0.8338155	3	0	0	3	5
44	-0.8338155	3	0	0	7	0
44	-0.8338155	3	0	0	7	0
128	0.60853009	1	2	0	2	3
128	0.60853009	4	0	0	0	1
128	0.60853009	4	0	0	0	4
128	0.60853009	4	0	0	0	13
129	-0.7467868	4	0	0	1	5
129	-0.7467868	4	0	0	1	3
129	-0.7467868	3	0	0	4	2
129	-0.7467868	4	0	0	2	8
129	-0.7467868	1	1	0	3	4
129	-0.7467868	3	0	0	2	3
129	-0.7467868	4	0	0	0	7
130	0.38891174	4	0	0	1	6
130	0.38891174	3	0	0	8	1
130	0.38891174	1	4	0	0	0
130	0.38891174	3	1	0	2	3
13	-0.6454721	3	0	0	1	3
13	-0.6454721	3	0	0	9	1
13	-0.6454721	3	0	0	11	0
13	-0.6454721	3	0	0	7	1
13	-0.6454721	1	6	0	1	0
13	-0.6454721	3	0	0	2	9
13	-0.6454721	4	1	0	1	7
13	-0.6454721	4	0	0	1	3
65	-0.5017981	1	7	0	0	0
65	-0.5017981	4	0	0	2	2
63	-0.4580645	3	3	0	3	0
63	-0.4580645	4	0	0	2	5
63	-0.4580645	4	0	0	2	5
63	-0.4580645	4	1	0	3	6
63	-0.4580645	3	0	0	0	7
63	-0.4580645	4	0	0	0	3
63	-0.4580645	3	0	0	2	6
63	-0.4580645	3	0	0	2	7
131	0.04935728	4	0	0	3	4
131	0.04935728	4	1	0	1	4
131	0.04935728	4	1	0	2	5
131	0.04935728	4	0	0	0	4
131	0.04935728	4	0	0	0	0
17	-0.5629747	4	0	0	1	5
17	-0.5629747	4	0	0	2	7
17	-0.5629747	4	0	0	2	5
17	-0.5629747	4	1	0	2	5
17	-0.5629747	1	1	0	2	3
132	0.16354351	4	0	0	1	6
132	0.16354351	4	2	0	2	1
132	0.16354351	4	0	0	0	0
59	-0.6506817	4	0	0	0	9
59	-0.6506817	4	0	0	0	5
60	0.26196807	4	0	0	0	5
60	0.26196807	4	0	0	0	0
13	-0.6454721	4	0	0	0	0
13	-0.6454721	4	0	0	0	0
13	-0.6454721	3	0	6	0	0
13	-0.6454721	3	0	0	5	0
13	-0.6454721	4	0	0	7	0
13	-0.6454721	3	0	0	9	0
13	-0.6454721	3	0	0	9	0
13	-0.6454721	3	0	2	5	0
13	-0.6454721	3	0	2	5	0
13	-0.6454721	4	3	2	0	0
13	-0.6454721	4	0	0	0	8
13	-0.6454721	3	0	2	1	0
13	-0.6454721	3	2	2	4	0
13	-0.6454721	3	0	1	4	0
13	-0.6454721	3	0	0	6	0
13	-0.6454721	3	0	3	3	0
13	-0.6454721	3	0	0	8	0
13	-0.6454721	4	0	0	4	2
13	-0.6454721	3	0	9	0	0
13	-0.6454721	3	0	6	0	0
13	-0.6454721	1	0	10	0	0
13	-0.6454721	1	1	1	4	0
13	-0.6454721	3	0	1	5	0
13	-0.6454721	3	0	0	10	0
13	-0.6454721	4	1	0	3	2
13	-0.6454721	3	0	0	6	0
13	-0.6454721	4	1	0	5	0
13	-0.6454721	4	1	0	1	0
13	-0.6454721	4	3	0	0	4
13	-0.6454721	4	0	0	4	2
13	-0.6454721	4	0	0	0	13
13	-0.6454721	4	0	0	0	10
13	-0.6454721	4	0	0	0	10
13	-0.6454721	4	0	0	0	7
13	-0.6454721	4	0	0	7	0
13	-0.6454721	4	0	0	0	4
13	-0.6454721	3	5	0	1	0
13	-0.6454721	4	2	0	0	4
133	-0.1391433	2	0	0	0	0
133	-0.1391433	2	0	3	0	0
133	-0.1391433	3	0	2	7	0
133	-0.1391433	3	0	0	0	0
133	-0.1391433	2	0	0	8	1
133	-0.1391433	2	0	10	0	0
133	-0.1391433	2	0	1	6	0
133	-0.1391433	2	0	1	3	0
133	-0.1391433	2	0	9	0	0
133	-0.1391433	2	0	5	2	0
133	-0.1391433	2	0	6	1	0
133	-0.1391433	2	0	5	0	0
133	-0.1391433	2	0	4	0	0
133	-0.1391433	2	0	1	3	0
133	-0.1391433	2	0	0	11	0
133	-0.1391433	3	0	1	7	0
133	-0.1391433	2	0	2	8	0
133	-0.1391433	2	0	1	6	0
133	-0.1391433	2	0	5	0	2
133	-0.1391433	2	0	2	2	0
133	-0.1391433	2	0	0	0	0
133	-0.1391433	2	0	5	0	0
133	-0.1391433	2	0	4	2	0
133	-0.1391433	2	0	8	0	0
133	-0.1391433	2	2	2	2	0
133	-0.1391433	2	0	2	1	0
133	-0.1391433	2	2	3	0	0
133	-0.1391433	2	2	0	1	0
133	-0.1391433	3	2	1	4	0
133	-0.1391433	1	5	0	2	0
133	-0.1391433	3	0	0	2	6
133	-0.1391433	3	0	0	4	0
133	-0.1391433	3	0	1	7	0
133	-0.1391433	3	0	0	7	0
133	-0.1391433	3	0	0	4	0
133	-0.1391433	3	0	0	11	0
133	-0.1391433	3	0	0	9	0
133	-0.1391433	4	1	0	4	4
133	-0.1391433	4	0	0	0	5
133	-0.1391433	4	3	0	0	5
133	-0.1391433	4	0	0	3	7
109	0.13340497	2	0	6	0	0

24	0.13049266	3	0	0	0	3
24	0.13049266	2	2	6	0	0
24	0.13049266	3	0	6	0	1
24	0.13049266	3	0	4	0	2
24	0.13049266	3	0	0	1	3
24	0.13049266	3	0	0	0	0
24	0.13049266	3	0	0	12	0
24	0.13049266	3	0	6	0	1
24	0.13049266	2	0	1	0	10
24	0.13049266	2	1	2	1	1
24	0.13049266	2	0	2	1	5
24	0.13049266	2	0	2	1	4
24	0.13049266	2	0	3	0	6
24	0.13049266	2	2	0	0	3
24	0.13049266	1	2	4	0	1
24	0.13049266	1	2	1	0	2
24	0.13049266	1	1	0	0	5
24	0.13049266	1	0	4	0	2
24	0.13049266	4	4	0	0	4
24	0.13049266	4	0	1	0	5
25	0.05050057	4	0	5	0	4
25	0.05050057	1	4	0	0	0
25	0.05050057	4	0	3	0	6
25	0.05050057	4	8	0	0	0
25	0.05050057	4	0	5	1	1
25	0.05050057	4	0	1	4	2
25	0.05050057	4	0	8	0	0
25	0.05050057	4	0	0	5	0
25	0.05050057	1	5	0	0	3
25	0.05050057	4	0	4	4	0
25	0.05050057	4	0	4	0	0
25	0.05050057	4	0	3	0	1
25	0.05050057	4	0	6	0	4
25	0.05050057	4	3	1	0	2
25	0.05050057	4	0	2	0	7
25	0.05050057	4	0	2	1	4
25	0.05050057	4	0	2	0	2
25	0.05050057	4	0	3	0	4
25	0.05050057	4	0	3	0	3
25	0.05050057	4	0	3	0	6
25	0.05050057	1	3	11	0	0
25	0.05050057	4	0	0	0	7
25	0.05050057	1	2	0	0	4
26	-0.4862185	2	0	4	2	0
26	-0.4862185	2	0	0	11	0
26	-0.4862185	2	0	4	0	0
26	-0.4862185	3	0	4	1	2
26	-0.4862185	2	0	6	0	0
26	-0.4862185	2	0	4	0	6
26	-0.4862185	2	0	0	0	0
26	-0.4862185	2	3	0	0	5
26	-0.4862185	2	1	0	2	2
26	-0.4862185	4	0	0	0	5
26	-0.4862185	4	0	0	0	6
26	-0.4862185	4	0	0	0	3
27	0.14236623	2	0	1	0	3
27	0.14236623	3	4	0	3	2
27	0.14236623	2	0	4	2	0
27	0.14236623	2	0	2	1	0
27	0.14236623	2	0	2	2	2
27	0.14236623	3	0	2	5	0
27	0.14236623	3	0	0	0	5
27	0.14236623	3	0	2	0	8
27	0.14236623	3	0	4	2	3
27	0.14236623	3	0	0	4	4
27	0.14236623	3	0	0	0	5
27	0.14236623	3	0	4	0	0
27	0.14236623	3	0	3	0	3
27	0.14236623	2	0	0	2	2
27	0.14236623	2	0	1	3	3
27	0.14236623	2	0	3	0	2
27	0.14236623	2	0	6	0	3
27	0.14236623	2	0	5	0	3
27	0.14236623	2	4	4	0	0
27	0.14236623	2	0	0	0	0
27	0.14236623	2	0	2	0	4
27	0.14236623	2	0	0	0	6
27	0.14236623	4	0	0	4	5
28	-0.164918	2	0	7	0	0
28	-0.164918	2	0	3	3	0
28	-0.164918	2	0	0	6	1
28	-0.164918	2	3	4	3	1
28	-0.164918	2	0	0	2	0
28	-0.164918	2	0	1	3	1
28	-0.164918	2	0	0	0	6
28	-0.164918	2	0	0	2	4
28	-0.164918	2	0	1	2	3
28	-0.164918	2	0	0	7	0
28	-0.164918	2	0	7	0	0
28	-0.164918	2	0	2	0	4
28	-0.164918	2	0	2	0	2
28	-0.164918	2	0	5	2	0
28	-0.164918	2	0	4	0	6
28	-0.164918	2	0	2	0	6
28	-0.164918	2	0	3	0	5
28	-0.164918	2	4	0	0	2
28	-0.164918	2	2	3	0	1
28	-0.164918	2	1	0	0	4
28	-0.164918	2	4	0	0	6
28	-0.164918	2	2	1	0	3
28	-0.164918	2	0	0	3	5
29	-0.4265799	4	4	2	1	0
29	-0.4265799	4	0	0	7	0
29	-0.4265799	4	5	0	0	0
29	-0.4265799	4	0	0	7	0
29	-0.4265799	4	0	0	0	0
29	-0.4265799	4	0	0	0	0
29	-0.4265799	4	0	0	5	3
29	-0.4265799	4	0	5	3	0
29	-0.4265799	4	0	6	0	0
29	-0.4265799	4	0	0	0	3
29	-0.4265799	4	0	0	0	0
29	-0.4265799	4	0	6	0	2
29	-0.4265799	4	0	0	0	0
29	-0.4265799	4	0	1	0	4
29	-0.4265799	4	0	6	0	0
29	-0.4265799	4	0	7	0	4
29	-0.4265799	4	3	2	0	0
29	-0.4265799	4	3	3	0	3
29	-0.4265799	4	0	6	0	0
29	-0.4265799	4	0	0	0	8
29	-0.4265799	4	0	1	1	5
29	-0.4265799	4	0	0	0	7
29	-0.4265799	4	0	0	0	0
29	-0.4265799	4	0	0	0	5
29	-0.4265799	4	0	0	0	4
30	-0.1017257	2	0	2	2	2
30	-0.1017257	3	0	0	6	0
30	-0.1017257	2	0	2	4	2
30	-0.1017257	2	3	2	2	2

44	-0.8338155	3	0	0	13	0
44	-0.8338155	3	0	0	6	0
44	-0.8338155	3	0	0	4	0
44	-0.8338155	3	0	0	0	0
44	-0.8338155	1	5	0	1	0
44	-0.8338155	1	5	0	0	0
44	-0.8338155	1	3	0	0	0
44	-0.8338155	1	7	0	0	0
44	-0.8338155	4	6	0	0	0
28	-0.164918	1	4	0	0	0
28	-0.164918	1	9	0	0	0
28	-0.164918	3	2	0	0	2
28	-0.164918	3	0	0	6	4
28	-0.164918	3	6	0	0	2
28	-0.164918	1	4	0	0	0
28	-0.164918	1	3	0	2	0
28	-0.164918	1	3	0	3	1
28	-0.164918	1	7	0	1	1
28	-0.164918	4	0	0	0	7
28	-0.164918	4	0	0	1	7
28	-0.164918	4	1	0	1	5
28	-0.164918	4	0	0	0	7
28	-0.164918	4	4	0	0	5
28	-0.164918	4	0	0	0	0
28	-0.164918	1	4	0	0	2
28	-0.164918	4	3	0	0	4
28	-0.164918	4	2	0	0	2
28	-0.164918	4	0	0	0	7
28	-0.164918	1	2	0	0	4
28	-0.164918	1	2	0	0	4
28	-0.164918	1	1	0	2	2
28	-0.164918	1	8	0	0	1
28	-0.164918	1	2	0	0	3
28	-0.164918	1	5	0	0	2
28	-0.164918	1	0	0	5	0
28	-0.164918	1	1	0	5	2
28	-0.164918	3	4	0	4	0
28	-0.164918	3	7	0	0	0
28	-0.164918	3	3	0	1	0
28	-0.164918	3	0	0	9	0
28	-0.164918	3	0	0	6	0
28	-0.164918	3	0	0	5	0
28	-0.164918	3	0	0	7	0
28	-0.164918	3	0	0	6	0
28	-0.164918	1	8	0	0	0
28	-0.164918	1	5	0	0	0
28	-0.164918	1	3	0	0	1
28	-0.164918	1	7	0	0	0
23	-0.918747	1	7	0	0	0
23	-0.918747	4	0	0	0	5
23	-0.918747	4	0	0	0	5
23	-0.918747	4	3	0	0	6
23	-0.918747	4	0	0	3	7
23	-0.918747	3	0	0	7	0
24	0.13049266	1	6	0	0	1
24	0.13049266	4	0	0	0	0
24	0.13049266	4	0	0	2	5
24	0.13049266	1	3	0	0	5
24	0.13049266	4	4	0	0	2
24	0.13049266	1	6	0	0	0
24	0.13049266	1	1	0	5	0
24	0.13049266	1	4	0	1	0
24	0.13049266	4	7	0	1	1
24	0.13049266	4	0	0	0	7
24	0.13049266	4	2	0	0	6
24	0.13049266	4	0	0	0	4
24	0.13049266	1	3	0	0	2
24	0.13049266	1	6	0	0	0
24	0.13049266	1	2	0	0	3
24	0.13049266	4	1	0	0	5
24	0.13049266	4	2	0	0	3
24	0.13049266	4	4	0	3	1
24	0.13049266	4	3	0	0	4
24	0.13049266	4	4	0	0	0
24	0.13049266	4	5	0	0	2
24	0.13049266	4	2	0	0	4
24	0.13049266	1	10	0	0	0
24	0.13049266	1	6	0	0	3
24	0.13049266	1	2	0	1	1
24	0.13049266	4	0	0	2	2
24	0.13049266	3	4	0	4	0
24	0.13049266	1	6	0	0	0
24	0.13049266	3	0	0	5	0
24	0.13049266	3	0	0	7	0
24	0.13049266	1	8	0	0	0
24	0.13049266	4	0	0	0	3
24	0.13049266	1	7	0	0	2
24	0.13049266	1	6	0	0	0
26	-0.4862185	1	2	0	2	1
26	-0.4862185	1	2	0	0	3
26	-0.4862185	1	2	0	0	2
26	-0.4862185	1	0	0	6	0
26	-0.4862185	1	3	0	2	0
26	-0.4862185	1	6	0	0	0
26	-0.4862185	1	3	0	0	2
26	-0.4862185	1	7	0	0	0
26	-0.4862185	1	0	0	0	0
26	-0.4862185	1	4	0	0	2
26	-0.4862185	1	11	0	0	0
26	-0.4862185	1	7	0	0	0
75	-0.8077959	4	12	0	0	0
75	-0.8077959	4	6	0	0	0
75	-0.8077959	4	0	0	0	0
75	-0.8077959	4	3	0	0	0
75	-0.8077959	1	7	0	0	4
75	-0.8077959	1	9	0	0	0
75	-0.8077959	1	5	0	2	0
75	-0.8077959	1	4	0	1	0
75	-0.8077959	4	3	0	5	0
75	-0.8077959	3	0	0	5	0
75	-0.8077959	3	2	0	4	0
75	-0.8077959	4	0	0	0	5
75	-0.8077959	1	2	0	0	3
75	-0.8077959	1	6	0	0	1
75	-0.8077959	4	1	0	0	7
75	-0.8077959	4	4	0	0	6
75	-0.8077959	4	4	0	0	1
75	-0.8077959	4	7	0	0	2
75	-0.8077959	1	4	0	0	1
75	-0.8077959	1	5	0	0	1
75	-0.8077959	1	5	0	0	3
75	-0.8077959	1	0	0	0	0
75	-0.8077959	4	8	0	0	0
75	-0.8077959	1	0	0	6	0
75	-0.8077959	1	5	0	0	0
75	-0.8077959	1	8	0	0	3
75	-0.8077959	1	8	0	0	0
75	-0.8077959	4	4	0	0	0
75	-0.8077959	1	4	0	4	0
75	-0.8077959	1	3	0	0	0

109	0.13340497	2	0	0	6	0
109	0.13340497	3	0	3	9	0
109	0.13340497	4	0	0	5	0
109	0.13340497	2	0	0	0	0
109	0.13340497	2	0	0	0	0
109	0.13340497	2	0	2	5	0
109	0.13340497	3	0	2	2	0
109	0.13340497	2	0	3	2	0
109	0.13340497	2	1	1	4	1
109	0.13340497	1	1	3	1	1
109	0.13340497	2	3	1	0	2
109	0.13340497	1	0	0	4	1
109	0.13340497	2	0	0	12	0
109	0.13340497	2	0	8	0	0
109	0.13340497	2	0	3	6	0
109	0.13340497	3	0	5	2	0
109	0.13340497	2	0	2	3	0
109	0.13340497	1	1	1	2	0
109	0.13340497	3	0	0	7	0
109	0.13340497	2	0	0	8	0
109	0.13340497	3	0	0	6	0
109	0.13340497	3	0	1	6	0
109	0.13340497	3	0	0	6	4
109	0.13340497	2	0	6	0	1
109	0.13340497	2	1	2	3	0
109	0.13340497	2	0	3	0	0
109	0.13340497	2	0	6	0	0
109	0.13340497	2	0	0	5	0
109	0.13340497	2	0	9	0	0
109	0.13340497	2	2	6	0	0
109	0.13340497	3	2	2	4	0
109	0.13340497	2	0	6	0	0
109	0.13340497	1	3	1	1	0
109	0.13340497	2	0	3	3	0
109	0.13340497	4	0	12	0	0
109	0.13340497	4	0	0	0	9
109	0.13340497	2	0	2	7	0
109	0.13340497	3	1	0	5	1
109	0.13340497	3	3	0	5	0
109	0.13340497	3	3	0	0	3
109	0.13340497	1	5	0	1	2
109	0.13340497	1	2	0	0	2
109	0.13340497	4	0	0	0	5
109	0.13340497	1	9	0	0	0
109	0.13340497	1	7	0	0	0
109	0.13340497	4	0	0	0	8
109	0.13340497	4	0	0	0	7
109	0.13340497	4	0	0	0	7
109	0.13340497	4	0	0	0	9
12	0.03251334	1	0	0	2	3
12	0.03251334	4	0	0	0	9
12	0.03251334	3	0	0	11	0
12	0.03251334	4	0	0	5	0
12	0.03251334	3	0	2	3	0
12	0.03251334	3	0	0	0	0
12	0.03251334	3	3	1	2	1
12	0.03251334	3	0	0	0	8
12	0.03251334	3	0	14	0	0
12	0.03251334	2	0	6	2	0
12	0.03251334	2	0	1	11	0
12	0.03251334	3	0	0	0	0
12	0.03251334	3	0	3	4	0
12	0.03251334	3	0	0	2	2
12	0.03251334	2	0	4	0	0
12	0.03251334	2	0	5	0	0
12	0.03251334	2	0	8	0	0
12	0.03251334	3	0	6	1	0
12	0.03251334	3	0	7	1	0
12	0.03251334	2	0	4	0	0
12	0.03251334	2	0	3	2	0
12	0.03251334	3	0	7	0	0
12	0.03251334	1	5	1	0	0
12	0.03251334	2	0	1	1	0
12	0.03251334	3	0	0	8	0
12	0.03251334	1	7	2	0	0
12	0.03251334	3	3	0	4	0
12	0.03251334	4	0	0	0	7
12	0.03251334	3	0	0	0	0
12	0.03251334	3	0	1	4	0
12	0.03251334	3	0	0	8	0
12	0.03251334	3	0	0	3	2
12	0.03251334	4	0	0	1	6
12	0.03251334	4	2	0	0	4
12	0.03251334	4	0	0	0	0
12	0.03251334	4	0	0	0	5
12	0.03251334	1	4	0	0	0
123	0.10404049	3	0	0	0	0
123	0.10404049	3	0	2	2	0
123	0.10404049	3	0	0	6	2
123	0.10404049	3	0	3	5	0
123	0.10404049	3	2	0	4	0
123	0.10404049	3	0	4	5	0
123	0.10404049	3	1	2	4	0
123	0.10404049	3	0	3	5	0
123	0.10404049	3	0	0	6	0
123	0.10404049	3	0	0	6	3
123	0.10404049	3	1	8	0	0
123	0.10404049	2	1	5	0	0
123	0.10404049	3	2	0	2	3
123	0.10404049	4	1	0	0	5
123	0.10404049	4	2	0	0	5
123	0.10404049	4	0	0	0	4
61	-0.2053642	3	0	0	1	0
61	-0.2053642	3	0	6	0	0
61	-0.2053642	3	0	2	5	0
61	-0.2053642	3	0	1	6	0
61	-0.2053642	3	0	0	11	0
61	-0.2053642	3	0	0	11	0
61	-0.2053642	3	0	0	6	0
61	-0.2053642	3	0	0	6	0
61	-0.2053642	3	0	4	2	0
61	-0.2053642	3	0	0	7	4
61	-0.2053642	3	0	0	0	8
61	-0.2053642	3	2	0	2	3
61	-0.2053642	3	0	0	3	0
61	-0.2053642	4	4	0	1	3
61	-0.2053642	4	1	0	0	10
61	-0.2053642	4	0	0	0	8
65	-0.5017981	3	0	0	4	1
65	-0.5017981	3	0	0	6	0
65	-0.5017981	3	0	3	4	0
65	-0.5017981	3	0	0	8	0
65	-0.5017981	3	0	0	7	0
108	-0.0297885	3	0	1	4	1
108	-0.0297885	3	0	0	0	0
108	-0.0297885	3	0	8	2	0
108	-0.0297885	2	2	1	4	0
108	-0.0297885	4	2	2	3	0
108	-0.0297885	4	2	4	2	0
108	-0.0297885	1	4	6	0	0

30	-0.1017257	2	0	2	6	0
30	-0.1017257	2	0	1	4	2
30	-0.1017257	2	0	0	10	0
30	-0.1017257	2	0	0	7	0
30	-0.1017257	2	0	3	0	6
30	-0.1017257	2	0	3	0	10
30	-0.1017257	2	0	7	2	0
30	-0.1017257	2	0	3	0	6
30	-0.1017257	2	0	3	0	3
30	-0.1017257	2	3	1	0	2
30	-0.1017257	1	2	0	0	4
30	-0.1017257	1	5	0	0	1
30	-0.1017257	4	0	0	0	0
30	-0.1017257	4	0	2	0	2
30	-0.1017257	2	0	1	0	6
31	0.27911465	4	0	3	1	2
31	0.27911465	4	0	0	3	4
31	0.27911465	4	0	0	3	2
31	0.27911465	4	2	1	0	7
31	0.27911465	4	0	0	0	12
31	0.27911465	4	0	2	0	7
31	0.27911465	4	0	0	0	5
31	0.27911465	4	0	6	0	0
31	0.27911465	4	0	8	0	0
31	0.27911465	4	0	0	2	6
31	0.27911465	4	0	2	0	2
31	0.27911465	4	0	5	0	2
31	0.27911465	4	3	2	0	3
31	0.27911465	4	0	0	0	7
31	0.27911465	4	0	0	0	6
31	0.27911465	4	0	0	0	7
31	0.27911465	4	2	0	0	6
32	-0.8084256	2	0	4	0	2
32	-0.8084256	2	0	0	0	5
33	-0.9658134	4	0	4	0	4
33	-0.9658134	4	2	2	0	2
33	-0.9658134	4	0	0	0	0
33	-0.9658134	4	0	0	0	8
33	-0.9658134	4	0	4	1	2
33	-0.9658134	4	0	0	0	0
33	-0.9658134	4	0	3	0	4
33	-0.9658134	4	0	0	0	16
33	-0.9658134	4	0	2	0	6
33	-0.9658134	4	0	0	0	0
33	-0.9658134	4	0	4	0	4
33	-0.9658134	4	0	0	0	11
33	-0.9658134	4	0	2	3	3
33	-0.9658134	4	0	9	1	0
33	-0.9658134	4	0	2	0	2
33	-0.9658134	4	0	5	0	2
33	-0.9658134	4	0	1	0	6
33	-0.9658134	4	0	9	0	3
33	-0.9658134	4	2	0	0	6
33	-0.9658134	4	0	0	0	0
33	-0.9658134	4	2	2	0	1
33	-0.9658134	4	2	2	0	0
33	-0.9658134	4	6	0	0	2
33	-0.9658134	4	5	0	0	2
33	-0.9658134	4	3	2	0	5
33	-0.9658134	4	2	0	0	4
33	-0.9658134	4	0	1	0	4
33	-0.9658134	4	0	1	2	5
33	-0.9658134	4	0	1	0	8
33	-0.9658134	4	0	0	0	15
33	-0.9658134	4	2	0	0	5
34	-0.0341764	2	0	2	0	6
34	-0.0341764	2	0	0	0	0
34	-0.0341764	2	0	0	0	11
34	-0.0341764	2	0	2	2	4
34	-0.0341764	2	3	3	0	0
34	-0.0341764	2	3	2	0	2
34	-0.0341764	2	3	2	0	4
34	-0.0341764	1	3	0	0	3
34	-0.0341764	1	0	3	0	5
34	-0.0341764	2	0	4	0	1
34	-0.0341764	4	0	0	0	7
35	0.08994369	4	0	0	0	5
35	0.08994369	1	1	0	0	5
35	0.08994369	1	7	0	0	3
35	0.08994369	1	9	0	0	1
35	0.08994369	1	8	0	0	0
35	0.08994369	1	8	0	0	0
35	0.08994369	1	8	0	0	0
35	0.08994369	1	1	0	0	5
35	0.08994369	1	0	0	0	2
35	0.08994369	1	0	0	0	8
35	0.08994369	1	4	0	1	0
35	0.08994369	2	0	12	0	0
35	0.08994369	2	0	4	0	0
35	0.08994369	3	0	6	3	0
35	0.08994369	4	0	0	0	8
35	0.08994369	4	0	0	0	6
35	0.08994369	4	1	0	0	3
35	0.08994369	4	0	0	0	6
35	0.08994369	4	2	0	0	2
35	0.08994369	4	0	0	0	0
35	0.08994369	4	3	0	0	3
35	0.08994369	1	11	0	0	0
35	0.08994369	2	1	2	0	0
35	0.08994369	2	0	0	0	5
35	0.08994369	2	0	0	0	4
35	0.08994369	2	0	1	0	5
35	0.08994369	4	0	0	0	8
35	0.08994369	4	0	0	0	4
35	0.08994369	4	0	0	0	7
35	0.08994369	4	0	0	0	6
35	0.08994369	1	5	0	0	0
35	0.08994369	1	8	0	0	0
36	-0.6623215	4	9	0	0	0
36	-0.6623215	4	0	0	0	0
36	-0.6623215	4	0	0	0	9
36	-0.6623215	4	0	0	4	2
36	-0.6623215	3	0	12	1	0
36	-0.6623215	4	0	0	0	6
36	-0.6623215	4	0	0	0	5
36	-0.6623215	4	3	0	0	3
36	-0.6623215	4	2	0	0	1
36	-0.6623215	2	0	5	0	0
36	-0.6623215	4	0	9	0	0
36	-0.6623215	4	0	0	0	5
36	-0.6623215	4	2	0	0	0
36	-0.6623215	4	3	0	0	4
37	-0.7675263	1	6	0	0	1
37	-0.7675263	1	5	0	0	0
37	-0.7675263	1	10	0	0	1
37	-0.7675263	1	0	0	0	7
37	-0.7675263	4	0	0	0	5
37	-0.7675263	4	0	0	0	6
37	-0.7675263	3	0	4	0	0
37	-0.7675263	2	0	5	2	3
75	-0.8077959	1	0	0	0	0
75	-0.8077959	3	0	0	10	0
75	-0.8077959	3	0	0	6	0
75	-0.8077959	4	5	0	0	3
75	-0.8077959	1	5	0	0	2
75	-0.8077959	1	12	0	0	0
75	-0.8077959	1	7	0	0	0
75	-0.8077959	1	7	0	0	0
75	-0.8077959	1	6	0	0	0
75	-0.8077959	4	5	0	0	1
76	-0.2397604	3	5	0	2	0
76	-0.2397604	3	2	0	3	0
76	-0.2397604	4	0	0	0	10
76	-0.2397604	4	0	0	0	9
45	-1.162141	1	6	0	1	1
45	-1.162141	1	0	0	6	0
45	-1.162141	3	3	0	6	0
45	-1.162141	4	3	0	0	2
45	-1.162141	4	3	0	0	4
45	-1.162141	1	7	0	0	0
45	-1.162141	4	0	0	0	9
45	-1.162141	1	7	0	0	0
45	-1.162141	1	4	0	0	2
45	-1.162141	1	4	0	1	3
45	-1.162141	3	6	0	0	0
45	-1.162141	3	0	0	5	0
45	-1.162141	3	0	0	7	0
45	-1.162141	3	0	0	5	0
45	-1.162141	3	0	0	7	0
45	-1.162141	3	0	0	4	0
45	-1.162141	3	0	0	2	0
45	-1.162141	1	7	0	0	0
45	-1.162141	1	8	0	0	0
45	-1.162141	1	2	0	0	0
77	-0.7695051	3	4	0	1	1
77	-0.7695051	4	0	0	0	9
77	-0.7695051	4	1	0	0	8
77	-0.7695051	1	4	0	0	3
77	-0.7695051	1	6	0	0	3
77	-0.7695051	1	8	0	0	0
77	-0.7695051	4	0	0	2	4
77	-0.7695051	3	0	0	9	0
77	-0.7695051	1	7	0	0	0
77	-0.7695051	1	5	0	0	0
34	-0.0341764	1	0	0	6	1
34	-0.0341764	1	0	0	0	6
34	-0.0341764	1	7	0	0	2
34	-0.0341764	1	4	0	0	0
34	-0.0341764	1	5	0	0	0
34	-0.0341764	1	4	0	0	0
78	0.1284139	4	0	0	0	0
78	0.1284139	3	0	0	0	0
78	0.1284139	3	0	0	4	0
79	-1.0914428	1	4	0	2	0
79	-1.0914428	1	8	0	0	0
79	-1.0914428	4	0	0	0	8
79	-1.0914428	1	4	0	0	2
79	-1.0914428	1	4	0	0	0
80	0.2570778	1	0	0	0	4
80	0.2570778	4	0	0	0	9
81	-0.5904784	1	5	0	0	0
81	-0.5904784	4	0	4	0	4
81	-0.5904784	2	0	4	5	0
81	-0.5904784	2	8	0	0	0
67	0.28948928	2	0	0	0	9
82	0.00219736	1	0	0	0	0
82	0.00219736	2	0	15	0	0
82	0.00219736	1	7	0	0	0
59	-0.6506817	3	0	0	0	0
59	-0.6506817	3	0	0	0	0
59	-0.6506817	3	0	0	0	0
59	-0.6506817	3	0	0	0	0
59	-0.6506817	3	0	0	0	0
59	-0.6506817	3	0	0	0	0
59	-0.6506817	3	0	0	0	0
59	-0.6506817	1	5	0	0	0
59	-0.6506817	1	4	0	0	0
59	-0.6506817	2	6	0	0	0
59	-0.6506817	1	9	0	0	0
59	-0.6506817	1	6	0	0	0
59	-0.6506817	1	9	0	0	0
59	-0.6506817	1	6	0	2	0
59	-0.6506817	1	8	0	0	0
59	-0.6506817	1	7	0	0	0
59	-0.6506817	1	4	0	0	0
59	-0.6506817	1	3	0	0	0
59	-0.6506817	1	8	0	1	0
59	-0.6506817	1	9	0	0	0
59	-0.6506817	3	11	0	0	0
59	-0.6506817	3	4	0	3	0
59	-0.6506817	1	6	0	4	0
59	-0.6506817	1	6	0	0	0
59	-0.6506817	1	1	4	2	0
61	-0.2053642	1	0	0	0	0
61	-0.2053642	1	0	0	0	0
61	-0.2053642	3	0	0	0	0
61	-0.2053642	1	5	0	0	0
61	-0.2053642	1	7	0	0	0
61	-0.2053642	1	0	0	0	0
61	-0.2053642	1	6	0	0	0
61	-0.2053642	1	6	0	0	0
61	-0.2053642	1	0	0	0	0
61	-0.2053642	1	5	0	0	0
61	-0.2053642	1	6	0	0	0
61	-0.2053642	1	0	0	0	0
61	-0.2053642	1	4	0	0	0
61	-0.2053642	1	4	0	0	0
61	-0.2053642	1	0	0	0	0
61	-0.2053642	1	9	0	0	0
61	-0.2053642	1	7	0	0	0
61	-0.2053642	1	0	0	6	0
61	-0.2053642	1	11	0	0	0
61	-0.2053642	1	0	0	0	0
12	0.03251334	1	13	0	0	0
12	0.03251334	1	6	0	0	0
12	0.03251334	1	4	0	0	0
12	0.03251334	1	8	0	0	0
12	0.03251334	1	6	0	0	0
12	0.03251334	1	8	0	0	0
12	0.03251334	1	6	0	0	0
12	0.03251334	1	9	0	0	0
12	0.03251334	1	5	0	0	0
12	0.03251334	1	0	0	0	0
12	0.03251334	1	8	0	0	0
12	0.03251334	1	0	0	0	0
12	0.03251334	1	0	0	0	0
12	0.03251334	1	8	0	0	0
12	0.03251334	1	7	0	0	0
12	0.03251334	1	10	0	0	0
12	0.03251334	1	11	0	0	0
108	-0.0297885	3	0	0	4	0
108	-0.0297885	2	0	3	0	0
108	-0.0297885	2	0	9	1	0
108	-0.0297885	2	0	2	3	0
108	-0.0297885	2	0	5	2	0
108	-0.0297885	2	0	0	10	0
108	-0.0297885	3	0	2	5	0
108	-0.0297885	2	0	1	4	0
108	-0.0297885	2	0	6	0	0
108	-0.0297885	3	0	0	8	0
108	-0.0297885	3	0	0	12	0
108	-0.0297885	3	0	0	5	0
108	-0.0297885	3	0	1	6	0
108	-0.0297885	3	0	0	4	3
108	-0.0297885	4	0	3	2	1
108	-0.0297885	2	0	6	0	0
108	-0.0297885	2	0	10	0	0
108	-0.0297885	3	0	0	0	0
108	-0.0297885	2	0	4	0	0
108	-0.0297885	3	0	7	0	0
108	-0.0297885	1	2	0	0	0
108	-0.0297885	1	4	2	0	0
108	-0.0297885	4	5	0	1	3
108	-0.0297885	4	1	0	0	8
108	-0.0297885	4	4	0	0	2
108	-0.0297885	4	0	0	0	5
108	-0.0297885	1	6	0	0	0
108	-0.0297885	4	0	0	0	8
108	-0.0297885	4	0	0	0	9
108	-0.0297885	4	4	0	0	1
108	-0.0297885	4	0	0	0	6
108	-0.0297885	4	0	0	0	0
83	-0.1574278	2	0	0	4	2
83	-0.1574278	3	0	9	0	0
83	-0.1574278	3	0	2	3	0
83	-0.1574278	2	0	4	1	0
83	-0.1574278	3	0	3	4	0
83	-0.1574278	2	0	0	5	0
83	-0.1574278	3	0	2	4	0
83	-0.1574278	3	3	0	6	0
83	-0.1574278	3	1	0	6	0
83	-0.1574278	4	0	0	8	2
83	-0.1574278	2	0	7	0	0
83	-0.1574278	2	0	6	1	0
83	-0.1574278	2	0	9	0	0
83	-0.1574278	2	0	5	1	0
83	-0.1574278	3	0	5	2	0
83	-0.1574278	3	4	2	3	0
83	-0.1574278	3	0	1	7	0
83	-0.1574278	1	7	2	0	0
83	-0.1574278	2	0	0	7	0
83	-0.1574278	1	0	0	7	1
83	-0.1574278	1	0	0	8	0
83	-0.1574278	1	4	0	0	4
83	-0.1574278	4	0	0	0	5
83	-0.1574278	1	2	0	0	8
83	-0.1574278	3	0	0	3	4
83	-0.1574278	4	5	0	0	2
83	-0.1574278	4	0	0	0	8
85	0.16154037	2	0	0	0	0
85	0.16154037	2	0	6	0	0
85	0.16154037	3	0	7	0	0
85	0.16154037	3	1	0	3	0
85	0.16154037	2	1	0	2	0
85	0.16154037	2	4	0	0	0
85	0.16154037	2	0	3	0	0
85	0.16154037	3	0	3	6	0
85	0.16154037	2	0	6	0	0
85	0.16154037	2	0	1	1	0
85	0.16154037	2	0	4	2	0
85	0.16154037	2	0	9	0	0
85	0.16154037	2	0	2	6	0
85	0.16154037	3	0	2	4	0
85	0.16154037	3	0	0	8	0
85	0.16154037	4	0	0	4	2
85	0.16154037	2	0	3	2	0
85	0.16154037	2	0	2	0	0
85	0.16154037	2	0	6	0	0
85	0.16154037	2	0	6	0	0
85	0.16154037	2	0	2	5	0
85	0.16154037	2	0	9	0	0
85	0.16154037	2	0	8	0	0
85	0.16154037	2	1	6	2	0
85	0.16154037	2	2	4	0	0
85	0.16154037	2	0	4	5	0
85	0.16154037	1	0	0	6	0
85	0.16154037	3	0	0	3	0
85	0.16154037	3	0	0	10	0
85	0.16154037	4	4	0	2	2
85	0.16154037	1	4	0	3	3
85	0.16154037	1	2	0	1	2
85	0.16154037	4	0	0	1	5
85	0.16154037	4	3	0	0	7
85	0.16154037	4	0	0	0	3
60	0.26196807	2	0	5	3	0
60	0.26196807	1	0	0	3	0
60	0.26196807	2	2	1	3	0
60	0.26196807	1	2	0	2	2
60	0.26196807	2	0	6	0	0
60	0.26196807	2	0	2	8	0
60	0.26196807	2	0	8	0	0
60	0.26196807	3	0	8	2	0
60	0.26196807	3	0	5	3	0
60	0.26196807	2	1	9	0	0
60	0.26196807	2	0	4	0	1
60	0.26196807	3	0	3	5	0
60	0.26196807	3	1	5	2	0
60	0.26196807	2	0	9	0	0
60	0.26196807	2	2	6	0	0
60	0.26196807	2	7	0	0	0
60	0.26196807	2	0	0	0	0
60	0.26196807	3	0	0	7	0
60	0.26196807	3	2	1	0	0
60	0.26196807	4	1	0	3	6
60	0.26196807	4	0	0	0	5
60	0.26196807	4	8	0	0	0
60	0.26196807	1	1	0	0	7
60	0.26196807	4	0	0	0	9
60	0.26196807	4	0	0	0	10
60	0.26196807	4	0	0	0	0
60	0.26196807	4	0	0	0	6
118	-0.0647231	3	0	2	4	0
118	-0.0647231	2	0	9	0	0
118	-0.0647231	3	0	2	5	0
118	-0.0647231	3	0	0	8	0
118	-0.0647231	3	0	11	0	0
118	-0.0647231	3	0	2	4	0
118	-0.0647231	3	0	0	6	0
118	-0.0647231	3	0	0	1	3
118	-0.0647231	3	0	0	2	5

37	-0.7675263	2	0	5	0	0
37	-0.7675263	4	0	0	0	6
37	-0.7675263	4	0	0	0	8
37	-0.7675263	1	4	0	0	3
37	-0.7675263	2	2	4	0	0
37	-0.7675263	3	0	2	4	0
37	-0.7675263	4	0	1	0	3
37	-0.7675263	4	0	1	0	3
37	-0.7675263	4	0	6	0	4
37	-0.7675263	4	0	0	0	8
37	-0.7675263	4	0	0	0	4
37	-0.7675263	4	0	0	0	4
37	-0.7675263	4	0	0	0	7
37	-0.7675263	4	0	0	0	6
37	-0.7675263	4	0	0	0	9
37	-0.7675263	4	0	0	0	0
37	-0.7675263	4	0	0	0	0
37	-0.7675263	1	10	0	0	0
37	-0.7675263	4	0	0	0	9
37	-0.7675263	4	0	0	0	0
37	-0.7675263	4	2	0	0	7
37	-0.7675263	4	0	0	0	11
37	-0.7675263	4	0	0	0	9
38	-0.6944448	1	8	0	0	1
38	-0.6944448	4	0	0	0	0
38	-0.6944448	4	1	0	0	4
38	-0.6944448	4	1	0	0	12
38	-0.6944448	2	2	3	0	0
38	-0.6944448	2	0	7	0	0
38	-0.6944448	2	0	2	1	1
38	-0.6944448	2	0	9	0	0
38	-0.6944448	2	0	3	5	0
38	-0.6944448	4	0	0	1	11
38	-0.6944448	4	0	0	0	3
38	-0.6944448	4	1	0	0	8
38	-0.6944448	4	0	0	0	7
38	-0.6944448	4	0	0	0	6
38	-0.6944448	4	0	0	0	10
38	-0.6944448	4	0	0	0	6
38	-0.6944448	4	0	0	0	0
38	-0.6944448	4	0	0	0	1
38	-0.6944448	4	0	0	0	8
38	-0.6944448	4	0	0	0	8
38	-0.6944448	4	0	0	0	3
38	-0.6944448	4	1	0	0	2
38	-0.6944448	1	3	0	0	0
38	-0.6944448	1	3	0	0	0
38	-0.6944448	4	6	0	0	1
38	-0.6944448	4	0	0	0	10
39	-0.8941953	4	0	0	0	10
39	-0.8941953	4	0	0	0	3
39	-0.8941953	4	0	0	0	6
39	-0.8941953	4	0	0	0	7
39	-0.8941953	4	0	0	0	7
39	-0.8941953	4	0	0	0	4
39	-0.8941953	4	0	0	0	7
39	-0.8941953	3	1	0	4	2
39	-0.8941953	4	1	0	0	10
39	-0.8941953	4	1	0	0	4
39	-0.8941953	4	1	0	1	3
39	-0.8941953	1	6	1	2	2
39	-0.8941953	2	2	4	0	0
39	-0.8941953	2	0	2	3	2
39	-0.8941953	2	0	11	0	0
39	-0.8941953	2	0	7	0	0
39	-0.8941953	2	0	8	0	1
39	-0.8941953	2	0	0	0	6
39	-0.8941953	4	0	0	0	4
39	-0.8941953	4	0	0	0	11
39	-0.8941953	4	0	0	0	11
39	-0.8941953	4	2	0	0	4
39	-0.8941953	4	0	0	0	4
39	-0.8941953	4	0	0	0	5
39	-0.8941953	4	4	0	0	3
39	-0.8941953	2	2	2	0	2
39	-0.8941953	2	1	3	0	0
39	-0.8941953	2	0	3	0	0
39	-0.8941953	4	0	2	3	0
39	-0.8941953	4	0	1	0	6
39	-0.8941953	4	0	0	0	4
39	-0.8941953	4	0	0	0	4
39	-0.8941953	4	5	0	0	3
39	-0.8941953	4	0	0	0	0
40	-0.9621317	1	5	0	0	0
40	-0.9621317	2	0	7	0	0
40	-0.9621317	2	0	6	0	2
40	-0.9621317	2	0	6	0	0
40	-0.9621317	2	0	8	0	0
40	-0.9621317	2	0	3	2	3
40	-0.9621317	2	0	3	0	1
40	-0.9621317	4	0	0	0	4
40	-0.9621317	4	0	0	0	4
40	-0.9621317	4	0	0	0	7
40	-0.9621317	4	0	6	0	2
40	-0.9621317	4	0	2	0	9
40	-0.9621317	4	0	0	0	7
40	-0.9621317	4	0	0	0	3
40	-0.9621317	4	0	0	0	9
40	-0.9621317	4	0	0	0	6
40	-0.9621317	4	0	0	0	7
40	-0.9621317	4	0	0	0	5
41	-0.7133892	4	0	0	0	8
41	-0.7133892	4	2	0	0	4
41	-0.7133892	4	4	0	0	7
41	-0.7133892	3	0	0	2	4
41	-0.7133892	1	8	1	0	0
41	-0.7133892	3	0	3	4	1
41	-0.7133892	2	0	7	0	0
41	-0.7133892	1	0	0	0	0
41	-0.7133892	2	0	0	0	0
41	-0.7133892	4	0	0	0	9
42	-0.700803	1	0	0	0	9
42	-0.700803	4	0	0	0	13
42	-0.700803	2	0	9	0	0
42	-0.700803	2	0	4	0	1
42	-0.700803	4	0	0	0	5
42	-0.700803	1	0	0	0	8
42	-0.700803	1	8	0	0	0
42	-0.700803	2	0	2	0	3
42	-0.700803	4	0	0	0	10
42	-0.700803	4	0	0	0	7
42	-0.700803	4	0	0	0	8
42	-0.700803	1	5	0	0	2
43	-0.2465452	3	0	0	6	2
43	-0.2465452	3	0	0	4	0
43	-0.2465452	4	1	0	0	4
43	-0.2465452	4	0	0	0	4
43	-0.2465452	4	0	0	2	2
43	-0.2465452	1	2	0	1	1
43	-0.2465452	1	1	4	0	0

12	0.03251334	2	0	0	0	0
13	-0.6454721	1	4	6	0	0
13	-0.6454721	2	6	0	0	0
13	-0.6454721	1	13	0	0	0
13	-0.6454721	1	3	0	0	0
13	-0.6454721	1	7	0	0	0
13	-0.6454721	1	0	0	0	0
13	-0.6454721	2	3	0	0	0
13	-0.6454721	2	0	5	1	2
83	-0.1574278	1	6	0	0	0
83	-0.1574278	1	11	0	0	0
83	-0.1574278	1	3	0	0	0
83	-0.1574278	1	13	0	0	0
83	-0.1574278	1	11	0	0	0
83	-0.1574278	1	8	0	0	0
83	-0.1574278	1	0	0	0	0
83	-0.1574278	1	0	0	0	0
83	-0.1574278	1	0	0	0	0
83	-0.1574278	1	7	0	0	0
83	-0.1574278	1	2	0	1	0
83	-0.1574278	1	11	0	0	0
83	-0.1574278	3	5	0	0	0
83	-0.1574278	1	12	0	0	0
83	-0.1574278	1	5	0	0	0
83	-0.1574278	1	8	0	0	0
58	0.44441689	2	4	0	0	0
58	0.44441689	1	4	0	0	0
58	0.44441689	1	10	0	0	0
58	0.44441689	1	5	0	0	0
58	0.44441689	1	3	0	0	0
58	0.44441689	1	5	0	1	0
58	0.44441689	1	9	0	0	0
58	0.44441689	1	0	0	0	0
58	0.44441689	1	7	0	0	0
58	0.44441689	1	12	0	0	0
58	0.44441689	1	12	0	0	0
58	0.44441689	2	0	0	0	8
57	0.46721311	1	12	0	0	0
57	0.46721311	1	9	0	0	0
57	0.46721311	2	0	8	0	0
57	0.46721311	4	0	0	0	0
62	-0.8150679	1	8	0	0	0
62	-0.8150679	1	8	0	0	0
62	-0.8150679	1	2	0	0	0
62	-0.8150679	1	11	0	0	0
62	-0.8150679	1	1	0	0	0
62	-0.8150679	1	5	0	0	0
62	-0.8150679	1	4	0	0	0
60	0.26196807	1	0	0	0	0
60	0.26196807	1	9	0	0	0
60	0.26196807	3	0	0	0	0
60	0.26196807	1	0	0	0	0
60	0.26196807	1	0	0	0	0
60	0.26196807	1	5	0	0	0
60	0.26196807	1	11	0	0	0
60	0.26196807	1	0	0	0	0
60	0.26196807	1	3	0	0	0
60	0.26196807	1	2	0	0	0
60	0.26196807	1	7	0	0	0
60	0.26196807	1	5	0	0	0
60	0.26196807	3	9	0	0	0
60	0.26196807	3	0	0	8	0
60	0.26196807	3	0	0	0	4
60	0.26196807	4	0	0	0	0
60	0.26196807	1	0	4	0	0
17	-0.5629747	1	6	0	0	0
17	-0.5629747	1	7	0	0	0
17	-0.5629747	1	8	0	0	0
17	-0.5629747	1	0	0	0	0
17	-0.5629747	1	6	0	0	0
17	-0.5629747	1	0	0	7	0
84	-1.0300019	1	9	0	0	0
84	-1.0300019	1	7	0	0	0
84	-1.0300019	1	0	0	0	0
84	-1.0300019	2	0	0	0	0
14	0.29399424	1	8	0	0	0
14	0.29399424	1	7	2	0	0
14	0.29399424	1	0	0	0	4
14	0.29399424	1	7	0	0	0
14	0.29399424	1	4	1	0	0
14	0.29399424	1	6	0	0	0
14	0.29399424	1	4	0	0	1
14	0.29399424	1	0	0	0	8
14	0.29399424	1	2	0	0	0
14	0.29399424	1	4	1	0	0
14	0.29399424	1	5	0	0	2
14	0.29399424	1	0	1	0	5
14	0.29399424	1	4	1	0	3
14	0.29399424	1	2	0	2	3
14	0.29399424	4	0	0	3	6
14	0.29399424	4	0	0	0	9
14	0.29399424	4	0	0	5	3
14	0.29399424	4	0	0	0	2
14	0.29399424	1	2	0	2	0
14	0.29399424	1	9	0	0	0
14	0.29399424	1	3	2	0	0
14	0.29399424	3	0	14	0	0
14	0.29399424	2	0	0	5	0
14	0.29399424	2	0	3	2	0
14	0.29399424	2	0	8	0	0
14	0.29399424	2	0	9	0	0
14	0.29399424	2	0	5	0	0
14	0.29399424	2	4	5	0	0
14	0.29399424	2	0	10	0	0
14	0.29399424	3	0	0	0	0
14	0.29399424	3	0	0	8	0
14	0.29399424	3	0	0	12	0
14	0.29399424	3	0	0	7	0
14	0.29399424	3	0	0	0	6
85	0.16154037	1	7	0	0	1
85	0.16154037	1	7	0	0	0
85	0.16154037	4	9	0	0	0
85	0.16154037	4	1	0	0	7
85	0.16154037	4	2	0	0	4
85	0.16154037	4	3	0	0	7
85	0.16154037	4	4	0	0	5
85	0.16154037	4	0	0	0	3
85	0.16154037	4	0	0	0	6
85	0.16154037	4	0	0	1	5
85	0.16154037	4	0	0	0	9
85	0.16154037	4	4	0	0	2
85	0.16154037	1	8	0	1	1
85	0.16154037	1	4	0	0	0
85	0.16154037	1	2	0	3	0
85	0.16154037	1	2	8	0	0
85	0.16154037	2	6	1	0	0
85	0.16154037	3	0	9	0	0
85	0.16154037	2	0	5	1	0
85	0.16154037	2	0	6	0	0
85	0.16154037	4	0	0	0	2

118	-0.0647231	4	0	0	0	11
86	-0.9389541	1	2	0	2	1
86	-0.9389541	3	0	0	11	0
86	-0.9389541	3	0	1	5	0
86	-0.9389541	3	0	0	8	0
86	-0.9389541	4	0	3	4	0
86	-0.9389541	3	0	4	3	0
86	-0.9389541	1	1	5	1	0
86	-0.9389541	3	0	0	5	0
86	-0.9389541	4	1	0	4	1
86	-0.9389541	3	0	0	0	3
86	-0.9389541	3	0	0	11	0
86	-0.9389541	3	0	0	10	0
86	-0.9389541	3	1	0	0	6
86	-0.9389541	4	2	0	0	2
86	-0.9389541	4	3	0	0	5
86	-0.9389541	4	0	0	0	0
58	0.44441689	3	3	1	2	1
58	0.44441689	3	1	7	1	0
58	0.44441689	2	0	2	2	0
58	0.44441689	3	0	4	4	0
58	0.44441689	1	0	8	0	0
58	0.44441689	3	0	0	0	6
58	0.44441689	4	4	0	0	4
58	0.44441689	1	4	0	0	4
58	0.44441689	4	2	0	0	2
58	0.44441689	4	0	0	0	9
58	0.44441689	4	0	0	0	10
57	0.46721311	3	2	2	5	1
57	0.46721311	4	3	4	1	1
57	0.46721311	3	2	0	2	2
57	0.46721311	3	0	8	2	0
57	0.46721311	2	1	2	1	0
57	0.46721311	2	0	3	0	0
57	0.46721311	2	0	4	4	0
57	0.46721311	2	0	4	0	0
57	0.46721311	4	0	2	2	2
57	0.46721311	3	0	2	1	0
57	0.46721311	2	0	2	1	0
57	0.46721311	3	0	0	9	0
57	0.46721311	3	1	0	2	1
57	0.46721311	4	5	0	0	0
57	0.46721311	1	5	0	0	2
57	0.46721311	4	1	0	0	4
57	0.46721311	1	11	0	0	0
57	0.46721311	4	0	0	0	9
57	0.46721311	4	0	0	0	2
57	0.46721311	4	0	0	0	8
57	0.46721311	4	0	0	0	11
57	0.46721311	4	0	0	0	8
57	0.46721311	4	0	0	0	4
115	-0.6635707	1	2	1	2	0
115	-0.6635707	1	0	0	0	0
115	-0.6635707	2	0	2	1	0
115	-0.6635707	2	0	3	5	0
115	-0.6635707	1	0	2	4	0
115	-0.6635707	3	0	1	5	0
115	-0.6635707	2	0	6	1	0
115	-0.6635707	2	0	4	0	0
115	-0.6635707	2	0	2	7	0
115	-0.6635707	3	0	0	7	0
115	-0.6635707	4	2	0	3	1
115	-0.6635707	1	3	0	3	2
115	-0.6635707	1	0	0	0	6
115	-0.6635707	4	1	0	0	7
115	-0.6635707	4	0	0	3	4
59	-0.6506817	3	0	6	0	0
59	-0.6506817	2	0	5	0	0
59	-0.6506817	2	0	0	0	0
59	-0.6506817	3	0	4	7	0
59	-0.6506817	2	0	0	5	0
59	-0.6506817	3	0	0	7	0
59	-0.6506817	2	0	0	7	0
59	-0.6506817	2	0	0	10	0
59	-0.6506817	2	0	0	5	3
59	-0.6506817	2	0	2	5	0
59	-0.6506817	2	0	11	0	0
59	-0.6506817	2	0	7	1	0
59	-0.6506817	3	0	4	4	0
59	-0.6506817	2	0	5	0	0
59	-0.6506817	3	1	7	0	0
59	-0.6506817	2	0	6	2	0
59	-0.6506817	2	3	8	1	0
59	-0.6506817	2	0	13	0	0
59	-0.6506817	3	0	3	4	0
59	-0.6506817	1	3	2	4	0
59	-0.6506817	2	0	0	8	0
59	-0.6506817	1	6	1	1	0
59	-0.6506817	3	0	0	0	0
59	-0.6506817	3	1	0	4	0
59	-0.6506817	3	0	0	6	1
59	-0.6506817	4	2	0	0	3
59	-0.6506817	4	3	0	0	3
59	-0.6506817	4	1	0	0	7
59	-0.6506817	1	4	0	0	2
59	-0.6506817	1	0	0	0	7
59	-0.6506817	4	0	0	1	7
59	-0.6506817	4	7	0	0	0
59	-0.6506817	4	0	0	0	8
59	-0.6506817	4	0	0	0	4
84	-1.0300019	2	0	4	2	0
84	-1.0300019	2	0	2	3	0
84	-1.0300019	2	0	5	2	0
84	-1.0300019	2	0	7	2	0
84	-1.0300019	3	0	4	2	0
84	-1.0300019	3	0	0	11	0
84	-1.0300019	3	4	0	0	3
84	-1.0300019	1	6	0	1	2
84	-1.0300019	1	4	0	0	4
84	-1.0300019	1	4	0	0	1
84	-1.0300019	4	0	0	0	5
84	-1.0300019	1	0	0	0	5
84	-1.0300019	4	0	0	0	7
112	-0.2892918	3	0	0	3	0
112	-0.2892918	3	0	1	6	0
112	-0.2892918	3	0	0	8	0
112	-0.2892918	3	0	4	1	1
112	-0.2892918	2	0	7	0	0
112	-0.2892918	2	0	7	0	0
112	-0.2892918	3	0	3	5	0
112	-0.2892918	3	0	1	5	0
112	-0.2892918	4	0	0	2	3
112	-0.2892918	3	2	0	8	0
112	-0.2892918	1	1	0	3	5
112	-0.2892918	4	0	0	0	4
126	-0.5074377	3	1	0	8	0
126	-0.5074377	2	0	5	0	0
126	-0.5074377	4	1	0	0	2
64	-0.5598805	3	0	1	6	0
64	-0.5598805	3	0	0	4	3

43	-0.2465452	2	0	9	0	0
43	-0.2465452	2	0	4	3	0
43	-0.2465452	2	0	7	0	0
43	-0.2465452	2	0	5	0	0
43	-0.2465452	2	0	7	0	1
43	-0.2465452	3	0	0	2	5
43	-0.2465452	4	0	0	0	7
43	-0.2465452	4	2	0	0	3
43	-0.2465452	1	1	0	0	3
43	-0.2465452	1	0	0	0	0
43	-0.2465452	3	0	3	4	0
43	-0.2465452	4	0	1	0	8
43	-0.2465452	4	0	0	0	3
43	-0.2465452	1	6	0	0	0
43	-0.2465452	1	2	0	0	4
43	-0.2465452	4	4	0	0	3
43	-0.2465452	1	0	0	0	3
43	-0.2465452	1	6	0	0	0
43	-0.2465452	1	0	0	0	3
44	-0.8338155	4	1	0	0	7
44	-0.8338155	4	0	0	0	11
44	-0.8338155	4	0	0	0	0
44	-0.8338155	4	0	0	0	7
44	-0.8338155	4	0	0	0	8
44	-0.8338155	4	0	0	0	7
45	-1.162141	4	1	0	1	2
45	-1.162141	2	3	9	0	0
45	-1.162141	2	0	9	0	0
45	-1.162141	4	0	1	3	2
45	-1.162141	2	0	8	0	0
45	-1.162141	2	0	3	0	2
45	-1.162141	2	0	9	0	0
45	-1.162141	2	0	9	0	0
45	-1.162141	2	0	6	0	0
45	-1.162141	2	0	5	0	0
45	-1.162141	2	0	4	0	4
45	-1.162141	4	0	0	2	4
45	-1.162141	4	0	0	0	12
45	-1.162141	4	0	0	0	10
45	-1.162141	4	4	0	0	4
45	-1.162141	4	0	0	0	0
45	-1.162141	4	0	0	0	10
45	-1.162141	4	0	0	0	9
45	-1.162141	4	0	0	0	12
45	-1.162141	4	0	0	0	5
45	-1.162141	4	3	0	0	4
45	-1.162141	2	7	0	0	0
45	-1.162141	2	1	3	0	0
45	-1.162141	2	0	3	4	0
45	-1.162141	4	0	0	0	8
45	-1.162141	4	0	0	0	8
45	-1.162141	4	0	0	0	8
45	-1.162141	4	0	0	0	10
45	-1.162141	4	0	0	0	7
45	-1.162141	4	0	0	0	5
45	-1.162141	4	0	0	0	4
46	-0.5263299	1	5	1	0	0
46	-0.5263299	3	0	6	3	0
46	-0.5263299	4	0	4	2	4
46	-0.5263299	2	0	8	0	0
46	-0.5263299	2	0	3	0	2
46	-0.5263299	4	0	0	0	11
46	-0.5263299	4	0	0	0	5
46	-0.5263299	4	0	0	0	11
46	-0.5263299	4	0	0	0	6
46	-0.5263299	4	0	0	0	6
46	-0.5263299	1	4	0	0	6
46	-0.5263299	4	0	0	0	9
46	-0.5263299	4	0	0	0	6
46	-0.5263299	4	0	0	0	2
46	-0.5263299	4	0	4	0	1
46	-0.5263299	4	0	0	0	6
46	-0.5263299	4	0	0	0	9
46	-0.5263299	4	0	0	0	5
46	-0.5263299	4	0	0	0	3
46	-0.5263299	4	0	0	0	8
46	-0.5263299	4	0	0	0	0
46	-0.5263299	4	4	0	0	3
47	-0.1698357	2	0	2	3	0
47	-0.1698357	2	0	7	0	0
47	-0.1698357	2	0	5	0	3
47	-0.1698357	3	0	3	3	0
47	-0.1698357	4	0	0	0	3
47	-0.1698357	4	0	0	0	7
47	-0.1698357	4	0	0	0	6
47	-0.1698357	4	4	0	0	2
47	-0.1698357	4	2	0	0	3
47	-0.1698357	2	0	0	0	4
47	-0.1698357	2	0	2	0	6
47	-0.1698357	2	0	0	0	7
47	-0.1698357	2	0	2	0	2
48	-0.632241	2	0	0	0	10
48	-0.632241	2	0	0	0	4
48	-0.632241	2	0	3	0	4
48	-0.632241	4	0	0	0	9
48	-0.632241	4	0	0	0	9
48	-0.632241	4	0	0	0	12
48	-0.632241	4	0	0	0	5
48	-0.632241	4	0	0	0	9
48	-0.632241	4	3	0	0	2
48	-0.632241	4	0	0	0	6
49	-0.0366355	4	0	0	0	0
49	-0.0366355	4	0	0	0	6
49	-0.0366355	3	0	0	0	0
49	-0.0366355	3	0	0	0	0
49	-0.0366355	4	0	0	0	0
49	-0.0366355	4	0	0	0	9
49	-0.0366355	3	0	0	0	0
49	-0.0366355	4	0	0	0	0
49	-0.0366355	4	0	0	0	0
49	-0.0366355	4	0	0	0	3
49	-0.0366355	4	0	0	0	5
49	-0.0366355	4	0	0	0	5
49	-0.0366355	3	0	0	0	9
49	-0.0366355	4	0	0	0	0
49	-0.0366355	4	0	0	0	0
49	-0.0366355	4	0	0	3	5
49	-0.0366355	4	0	0	0	3
49	-0.0366355	4	0	0	0	10
49	-0.0366355	4	0	0	0	0
49	-0.0366355	4	0	0	0	0
49	-0.0366355	4	0	0	0	0
49	-0.0366355	4	0	0	12	0
49	-0.0366355	4	0	0	9	0
50	-0.9402674	4	0	0	0	0
50	-0.9402674	4	0	0	0	6
50	-0.9402674	4	0	0	0	7
50	-0.9402674	4	0	0	0	7
50	-0.9402674	4	0	0	0	4
50	-0.9402674	3	0	0	0	0

85	0.16154037	3	0	0	7	0
85	0.16154037	3	0	1	3	0
82	0.00219736	4	0	0	0	11
82	0.00219736	4	4	0	0	4
82	0.00219736	4	0	0	0	8
82	0.00219736	4	2	1	0	1
82	0.00219736	1	4	0	0	2
82	0.00219736	4	4	1	0	2
82	0.00219736	1	2	0	0	2
82	0.00219736	4	2	0	0	4
82	0.00219736	3	0	0	0	3
82	0.00219736	4	1	0	1	2
82	0.00219736	1	0	0	0	6
82	0.00219736	4	0	0	1	9
82	0.00219736	4	0	0	1	5
82	0.00219736	4	0	0	0	6
82	0.00219736	4	0	0	1	3
82	0.00219736	4	0	0	0	9
82	0.00219736	4	0	0	0	9
82	0.00219736	4	2	0	0	1
82	0.00219736	3	3	0	2	0
82	0.00219736	2	8	0	0	0
82	0.00219736	2	3	4	0	0
82	0.00219736	3	0	3	2	0
82	0.00219736	2	0	5	0	0
82	0.00219736	2	0	5	1	0
82	0.00219736	2	0	4	3	0
82	0.00219736	3	0	3	0	0
82	0.00219736	2	7	0	0	0
82	0.00219736	2	6	0	0	0
82	0.00219736	3	0	0	0	0
82	0.00219736	2	2	5	0	0
82	0.00219736	3	0	2	0	0
82	0.00219736	3	4	3	0	0
82	0.00219736	3	0	4	3	0
82	0.00219736	2	0	0	0	7
82	0.00219736	2	2	3	4	0
82	0.00219736	3	0	4	5	0
82	0.00219736	3	0	0	3	0
82	0.00219736	3	0	0	6	0
82	0.00219736	3	0	0	7	0
82	0.00219736	3	0	0	6	0
82	0.00219736	3	0	0	9	0
59	-0.6506817	2	0	2	2	0
59	-0.6506817	2	6	0	0	0
59	-0.6506817	2	0	1	1	0
59	-0.6506817	3	0	0	4	0
59	-0.6506817	3	0	0	7	0
59	-0.6506817	3	0	0	0	0
59	-0.6506817	3	0	12	0	0
59	-0.6506817	3	0	3	2	0
59	-0.6506817	3	0	0	0	0
59	-0.6506817	3	0	12	0	0
59	-0.6506817	3	0	1	6	0
59	-0.6506817	3	1	0	3	0
59	-0.6506817	3	0	0	8	0
86	-0.9389541	3	2	5	0	0
86	-0.9389541	2	0	8	0	0
86	-0.9389541	3	0	4	3	0
86	-0.9389541	2	1	7	1	0
86	-0.9389541	2	0	3	2	0
87	0.1696964	1	0	8	0	0
87	0.1696964	1	0	0	0	0
10	-0.5315071	2	0	13	0	0
10	-0.5315071	3	1	4	1	0
10	-0.5315071	3	0	3	1	0
10	-0.5315071	4	0	0	7	0
10	-0.5315071	4	4	0	0	3
10	-0.5315071	2	0	3	0	1
10	-0.5315071	2	0	11	0	0
10	-0.5315071	3	0	5	2	0
10	-0.5315071	4	0	0	0	5
10	-0.5315071	3	0	0	2	0
10	-0.5315071	3	0	0	11	0
71	-1.1600835	3	0	2	6	0
71	-1.1600835	2	0	4	2	0
71	-1.1600835	1	0	0	0	3
71	-1.1600835	3	0	0	10	0
71	-1.1600835	3	0	0	9	0
60	0.26196807	1	0	5	0	0
60	0.26196807	2	0	10	0	0
60	0.26196807	2	0	3	1	0
60	0.26196807	3	0	5	2	0
60	0.26196807	1	0	0	4	0
60	0.26196807	3	0	0	6	0
60	0.26196807	3	0	0	4	0
60	0.26196807	3	0	0	0	0
60	0.26196807	3	0	0	4	0
60	0.26196807	3	0	0	5	0
60	0.26196807	4	0	0	0	0
60	0.26196807	4	0	0	0	0
60	0.26196807	3	0	0	6	0
88	-0.088144	3	4	0	2	0
88	-0.088144	3	0	0	0	0
88	-0.088144	3	0	0	8	0
88	-0.088144	3	0	0	6	0
88	-0.088144	3	0	0	4	0
88	-0.088144	3	0	0	13	0
88	-0.088144	3	0	0	9	0
88	-0.088144	3	0	0	5	0
88	-0.088144	3	0	0	8	0
88	-0.088144	4	0	0	0	0
88	-0.088144	3	0	0	7	2
89	0.45437887	3	0	0	5	1
89	0.45437887	2	0	0	4	2
90	-0.1162193	3	0	0	0	0
90	-0.1162193	4	0	0	5	0
90	-0.1162193	1	2	3	0	0
90	-0.1162193	2	0	8	2	0
90	-0.1162193	3	0	0	7	0
90	-0.1162193	2	0	9	0	0
76	-0.2397604	3	0	0	7	0
76	-0.2397604	1	0	4	2	0
76	-0.2397604	1	0	5	2	0
76	-0.2397604	2	0	8	0	0
76	-0.2397604	2	0	7	0	0
76	-0.2397604	4	0	7	2	0
76	-0.2397604	2	0	5	4	0
76	-0.2397604	2	0	11	0	0
76	-0.2397604	2	0	3	2	0
76	-0.2397604	3	0	0	3	0
76	-0.2397604	2	0	4	2	0
76	-0.2397604	4	1	7	1	0
76	-0.2397604	2	0	4	2	0
76	-0.2397604	4	0	3	3	0
91	-0.9607165	3	0	0	0	0
91	-0.9607165	2	0	0	0	0
91	-0.9607165	2	0	4	3	0
91	-0.9607165	2	0	8	1	0
91	-0.9607165	2	0	4	1	0

64	-0.5598805	2	2	4	0	0
64	-0.5598805	3	0	2	4	0
64	-0.5598805	4	1	0	0	3
113	-0.3337907	2	0	1	7	1
113	-0.3337907	3	0	0	9	0
113	-0.3337907	4	0	0	0	5
81	-0.5904784	3	0	0	6	0
81	-0.5904784	3	0	0	5	0
81	-0.5904784	3	2	0	0	4
81	-0.5904784	4	3	0	0	3
81	-0.5904784	4	0	0	0	7
119	0.06088944	3	2	0	8	0
119	0.06088944	3	0	0	9	0
119	0.06088944	4	4	0	3	1
119	0.06088944	4	1	0	0	7
119	0.06088944	4	0	0	4	5
119	0.06088944	4	0	0	0	8
119	0.06088944	4	0	0	0	5
119	0.06088944	4	0	0	0	2
62	-0.8150679	4	3	0	0	4
62	-0.8150679	4	5	0	0	1
62	-0.8150679	4	0	0	0	7
62	-0.8150679	4	0	0	0	7
62	-0.8150679	3	8	0	0	0
114	-0.4855974	4	4	0	0	2
114	-0.4855974	3	0	0	4	6
114	-0.4855974	4	0	0	0	4
88	-0.088144	1	0	0	0	0
88	-0.088144	4	0	0	0	4
134	-0.5095728	3	3	0	2	0
134	-0.5095728	3	0	0	4	0
134	-0.5095728	3	0	0	4	0
134	-0.5095728	3	0	0	0	0
134	-0.5095728	3	0	0	0	0
134	-0.5095728	3	0	0	1	1
134	-0.5095728	4	0	0	0	0
134	-0.5095728	3	0	0	3	2
134	-0.5095728	3	0	0	3	4
134	-0.5095728	3	0	0	6	0
134	-0.5095728	3	0	0	0	7
134	-0.5095728	3	1	0	0	9
134	-0.5095728	3	0	0	6	0
134	-0.5095728	3	0	0	5	0
134	-0.5095728	3	0	1	2	1
134	-0.5095728	3	4	2	1	0
134	-0.5095728	3	0	0	7	0
134	-0.5095728	3	2	0	4	0
135	-0.1308638	3	0	0	10	0
135	-0.1308638	2	0	0	0	0
135	-0.1308638	3	0	0	3	0
135	-0.1308638	3	0	0	12	2
135	-0.1308638	3	0	0	6	2
135	-0.1308638	3	0	0	0	0
135	-0.1308638	4	0	0	0	0
135	-0.1308638	3	0	0	0	9
135	-0.1308638	3	2	1	2	1
135	-0.1308638	3	0	0	6	4
135	-0.1308638	3	0	0	0	6
135	-0.1308638	4	0	0	0	8
135	-0.1308638	4	0	0	5	3
135	-0.1308638	4	0	0	4	4
135	-0.1308638	3	0	0	3	4
135	-0.1308638	3	3	0	0	2
135	-0.1308638	2	3	2	0	1
135	-0.1308638	3	5	0	6	0
135	-0.1308638	3	0	3	3	0
135	-0.1308638	2	10	0	0	0
135	-0.1308638	2	0	7	0	0
135	-0.1308638	3	1	1	5	0
135	-0.1308638	1	0	0	5	0
135	-0.1308638	1	10	0	0	0
135	-0.1308638	3	3	2	2	0
135	-0.1308638	3	0	0	6	0
135	-0.1308638	2	0	6	0	0
136	-0.0402076	3	0	0	6	0
136	-0.0402076	3	0	0	6	0
136	-0.0402076	3	0	0	0	0
136	-0.0402076	3	0	0	2	1
136	-0.0402076	3	0	0	10	0
136	-0.0402076	3	0	1	7	0
136	-0.0402076	3	0	0	5	0
77	-0.7695051	1	0	0	8	0
77	-0.7695051	4	6	0	0	1
77	-0.7695051	4	0	0	5	0
77	-0.7695051	4	0	0	0	9
77	-0.7695051	4	0	0	2	5
77	-0.7695051	1	7	0	0	0
77	-0.7695051	1	0	0	7	0
77	-0.7695051	2	0	8	0	0
77	-0.7695051	1	4	1	4	0
137	-0.2592855	4	0	0	0	12
137	-0.2592855	4	0	0	5	0
137	-0.2592855	4	0	0	0	6
137	-0.2592855	4	0	0	0	0
137	-0.2592855	4	0	0	10	0
137	-0.2592855	4	0	0	0	4
137	-0.2592855	4	2	0	2	2
137	-0.2592855	4	0	0	8	0
137	-0.2592855	4	0	0	0	9
137	-0.2592855	4	4	0	0	4
137	-0.2592855	4	0	0	3	4
137	-0.2592855	4	0	0	0	3
137	-0.2592855	1	4	0	2	2
137	-0.2592855	1	6	0	0	0
137	-0.2592855	3	0	0	8	0
137	-0.2592855	3	2	0	6	0
137	-0.2592855	3	0	0	7	0
137	-0.2592855	3	0	0	11	0
137	-0.2592855	1	7	0	0	0
137	-0.2592855	1	0	7	0	0
138	0.00681655	3	0	0	6	1
138	0.00681655	3	0	0	0	0
138	0.00681655	4	0	0	0	0
138	0.00681655	3	0	0	0	0
138	0.00681655	3	0	0	8	0
138	0.00681655	4	0	0	7	0
138	0.00681655	4	0	4	0	3
138	0.00681655	4	1	0	1	9
138	0.00681655	4	0	0	2	4
138	0.00681655	4	3	0	0	0
138	0.00681655	3	3	2	0	1
138	0.00681655	3	0	3	0	2
138	0.00681655	3	1	0	6	0
138	0.00681655	3	0	0	0	0
138	0.00681655	3	0	3	3	2
138	0.00681655	3	0	0	10	0
138	0.00681655	3	0	0	0	0
138	0.00681655	3	0	0	6	0
138	0.00681655	3	2	5	0	0
138	0.00681655	3	0	2	4	0

51	-0.9536858	4	0	0	0	5
51	-0.9536858	4	0	0	0	6
51	-0.9536858	4	0	0	0	0
51	-0.9536858	4	0	0	0	3
51	-0.9536858	4	0	0	6	0
51	-0.9536858	4	0	0	0	0
51	-0.9536858	4	0	0	0	0
51	-0.9536858	4	0	0	0	0
51	-0.9536858	4	0	0	0	6
51	-0.9536858	4	0	0	0	11
51	-0.9536858	4	0	0	0	5
51	-0.9536858	4	0	0	2	0
51	-0.9536858	4	0	0	0	5
51	-0.9536858	4	0	0	9	2
51	-0.9536858	4	0	0	0	0
51	-0.9536858	4	0	0	0	0
51	-0.9536858	4	0	0	0	4
51	-0.9536858	4	0	0	0	14
51	-0.9536858	4	0	0	0	7
51	-0.9536858	4	0	0	0	4
51	-0.9536858	4	0	0	0	0
51	-0.9536858	4	0	0	0	0
51	-0.9536858	4	0	0	0	0
51	-0.9536858	4	0	0	0	5
51	-0.9536858	4	0	0	0	12
51	-0.9536858	4	0	0	0	7
51	-0.9536858	4	0	0	2	1
51	-0.9536858	4	0	0	0	9
51	-0.9536858	3	0	0	5	0
51	-0.9536858	4	0	0	0	0
51	-0.9536858	4	0	0	0	0
51	-0.9536858	4	0	0	0	0
51	-0.9536858	4	0	0	2	6
51	-0.9536858	4	0	0	0	10
51	-0.9536858	4	0	0	4	3
51	-0.9536858	4	0	0	3	3
51	-0.9536858	3	0	0	3	2
51	-0.9536858	3	0	0	0	0
51	-0.9536858	3	0	0	6	0
51	-0.9536858	3	0	0	3	0
51	-0.9536858	3	0	0	6	0
51	-0.9536858	3	0	0	8	0
51	-0.9536858	3	0	0	0	0
52	-0.9820581	4	0	0	0	0
52	-0.9820581	3	0	0	5	0
53	-0.1890367	4	0	0	0	5
53	-0.1890367	3	0	0	8	0
53	-0.1890367	3	0	0	0	7
53	-0.1890367	3	0	0	0	7
53	-0.1890367	3	0	0	0	0
53	-0.1890367	3	0	0	4	1
53	-0.1890367	4	0	0	6	4
53	-0.1890367	3	0	0	0	0
53	-0.1890367	3	0	0	6	0
53	-0.1890367	3	0	0	0	0
53	-0.1890367	3	0	0	6	0
53	-0.1890367	3	0	0	0	0
54	-0.7758386	4	0	0	7	3
54	-0.7758386	4	0	0	0	2
54	-0.7758386	4	0	0	0	0
54	-0.7758386	4	0	0	0	8
54	-0.7758386	4	0	0	0	4
54	-0.7758386	4	0	0	2	5
54	-0.7758386	4	0	0	0	3
54	-0.7758386	4	0	0	0	4
54	-0.7758386	4	0	0	0	4
54	-0.7758386	4	0	0	0	0
54	-0.7758386	4	0	0	0	4
54	-0.7758386	4	0	0	0	3
54	-0.7758386	4	0	0	0	6
54	-0.7758386	4	0	0	0	2
54	-0.7758386	3	0	0	0	12
54	-0.7758386	4	0	0	4	0
54	-0.7758386	4	0	0	0	10
54	-0.7758386	4	0	0	1	3
54	-0.7758386	4	0	0	0	3
54	-0.7758386	4	0	0	0	7
54	-0.7758386	3	0	0	1	8
54	-0.7758386	3	0	0	6	0
54	-0.7758386	3	0	0	9	0
54	-0.7758386	3	0	0	14	0
54	-0.7758386	3	0	0	8	1
54	-0.7758386	3	0	0	7	0
54	-0.7758386	3	0	0	8	0
54	-0.7758386	3	0	0	8	0
54	-0.7758386	3	0	0	6	0
55	-0.5781918	4	0	0	0	5
55	-0.5781918	4	0	0	0	5
55	-0.5781918	4	0	0	0	4
55	-0.5781918	3	0	0	6	0
55	-0.5781918	3	0	0	16	0
55	-0.5781918	3	0	0	8	0
55	-0.5781918	4	0	0	6	0
55	-0.5781918	3	0	0	5	2
55	-0.5781918	3	0	0	3	3
55	-0.5781918	3	0	0	10	0
56	-0.4904883	4	0	0	10	0
56	-0.4904883	4	0	0	4	5
56	-0.4904883	3	0	0	0	10
56	-0.4904883	3	0	0	5	2
56	-0.4904883	3	0	0	7	0
56	-0.4904883	3	0	0	3	0
56	-0.4904883	3	0	0	0	0
56	-0.4904883	3	0	0	8	0
56	-0.4904883	3	0	0	9	0
12	0.03251334	1	0	0	0	0
12	0.03251334	1	0	0	0	0
12	0.03251334	1	5	0	0	0
12	0.03251334	1	7	0	0	0
12	0.03251334	2	0	0	0	0
12	0.03251334	1	5	0	0	0
12	0.03251334	1	4	0	0	0
12	0.03251334	1	0	0	0	8
12	0.03251334	1	0	0	0	0
12	0.03251334	1	2	0	0	2
12	0.03251334	1	10	0	0	0
12	0.03251334	1	0	0	0	5
12	0.03251334	1	0	0	0	11
12	0.03251334	1	3	0	0	2
12	0.03251334	1	3	0	0	2
12	0.03251334	4	0	0	0	9
12	0.03251334	1	6	0	0	0
12	0.03251334	1	0	0	0	5
12	0.03251334	1	2	0	4	1
12	0.03251334	1	2	0	0	2
12	0.03251334	1	0	0	0	8
12	0.03251334	1	5	0	0	1
12	0.03251334	1	3	0	2	0
12	0.03251334	1	0	0	0	0
12	0.03251334	1	11	0	0	0

91	-0.9607165	2	0	3	0	0
91	-0.9607165	2	0	0	0	0
91	-0.9607165	2	1	3	2	0
91	-0.9607165	2	1	2	0	0
91	-0.9607165	2	0	3	0	0
91	-0.9607165	2	0	4	2	0
91	-0.9607165	2	0	5	3	0
91	-0.9607165	2	0	4	3	0
91	-0.9607165	2	0	5	2	1
91	-0.9607165	2	0	3	1	0
91	-0.9607165	3	0	0	0	0
92	-0.7912643	2	2	3	2	0
92	-0.7912643	1	1	1	3	0
92	-0.7912643	1	2	0	1	3
92	-0.7912643	2	0	6	0	0
93	-1.0759657	3	3	0	5	0
93	-1.0759657	3	2	2	3	0
93	-1.0759657	3	0	6	2	0
93	-1.0759657	3	0	8	1	0
93	-1.0759657	4	0	8	0	0
93	-1.0759657	3	0	0	0	0
93	-1.0759657	3	0	3	3	0
93	-1.0759657	3	0	0	6	0
93	-1.0759657	3	0	0	0	0
93	-1.0759657	3	0	2	0	0
93	-1.0759657	4	0	9	0	0
93	-1.0759657	4	0	7	0	1
93	-1.0759657	3	1	0	5	0
93	-1.0759657	3	1	1	2	0
93	-1.0759657	3	1	5	1	0
93	-1.0759657	3	0	9	0	0
93	-1.0759657	3	0	8	1	0
93	-1.0759657	2	0	10	0	0
93	-1.0759657	3	0	6	1	0
93	-1.0759657	3	0	3	0	0
93	-1.0759657	2	0	2	2	0
93	-1.0759657	2	0	2	0	0
93	-1.0759657	2	0	8	0	0
93	-1.0759657	4	0	0	5	0
93	-1.0759657	3	0	6	1	0
93	-1.0759657	3	0	2	1	0
93	-1.0759657	3	0	0	0	0
93	-1.0759657	3	0	8	0	0
93	-1.0759657	3	0	1	9	0
93	-1.0759657	3	0	6	2	0
93	-1.0759657	4	0	3	0	0
93	-1.0759657	3	0	5	0	0
93	-1.0759657	3	0	2	3	0
93	-1.0759657	3	0	8	0	0
93	-1.0759657	3	0	1	4	1
93	-1.0759657	3	0	3	6	0
93	-1.0759657	2	0	10	0	0
93	-1.0759657	4	0	0	0	7
93	-1.0759657	4	0	0	0	0
93	-1.0759657	4	0	0	0	10
93	-1.0759657	4	0	1	0	6
93	-1.0759657	4	0	0	0	7
94	0.40549992	1	3	0	4	0
94	0.40549992	1	4	0	0	0
94	0.40549992	2	0	3	2	0
94	0.40549992	2	0	4	2	0
94	0.40549992	2	0	4	2	0
94	0.40549992	2	0	2	3	0
94	0.40549992	1	0	5	1	0
94	0.40549992	2	1	2	4	0
94	0.40549992	2	1	2	2	0
94	0.40549992	2	0	4	0	0
94	0.40549992	2	0	3	2	0
94	0.40549992	2	0	6	2	0
94	0.40549992	2	0	8	1	0
94	0.40549992	2	0	6	0	0
94	0.40549992	2	0	0	9	0
94	0.40549992	2	0	2	8	0
94	0.40549992	2	0	7	2	0
94	0.40549992	2	0	8	0	0
94	0.40549992	2	0	4	2	0
94	0.40549992	2	0	5	2	1
94	0.40549992	4	0	0	1	3
95	-0.6891233	2	1	2	2	0
95	-0.6891233	2	0	6	4	0
95	-0.6891233	2	0	3	1	0
95	-0.6891233	2	0	6	0	0
95	-0.6891233	2	0	6	1	0
95	-0.6891233	2	0	4	2	1
95	-0.6891233	2	0	5	0	0
95	-0.6891233	2	0	5	1	0
95	-0.6891233	2	0	3	3	0
95	-0.6891233	2	0	5	0	0
95	-0.6891233	2	0	2	1	0
95	-0.6891233	2	0	7	2	0
95	-0.6891233	2	1	2	6	0
96	-0.8769027	2	0	3	2	0
96	-0.8769027	3	0	0	6	0
96	-0.8769027	2	0	4	2	0
96	-0.8769027	3	3	4	0	0
96	-0.8769027	2	0	2	2	0
96	-0.8769027	2	2	3	3	0
96	-0.8769027	1	0	6	0	0
96	-0.8769027	2	0	2	1	0
96	-0.8769027	2	0	4	1	0
96	-0.8769027	2	0	4	0	0
96	-0.8769027	2	0	4	2	0
96	-0.8769027	2	0	2	5	1
96	-0.8769027	2	0	6	3	0
96	-0.8769027	2	0	4	3	0
96	-0.8769027	2	0	2	4	0
96	-0.8769027	2	0	3	4	0
96	-0.8769027	3	0	0	9	0
96	-0.8769027	3	0	0	7	0
96	-0.8769027	3	0	0	5	0
96	-0.8769027	2	0	0	0	0
96	-0.8769027	3	0	0	7	0
96	-0.8769027	4	0	0	0	2
96	-0.8769027	4	0	0	0	9
96	-0.8769027	4	0	0	0	4
96	-0.8769027	2	0	4	0	2
96	-0.8769027	4	0	1	1	4
97	0.10978981	2	0	3	5	0
97	0.10978981	3	0	2	6	0
97	0.10978981	3	0	2	3	0
97	0.10978981	1	3	0	1	2
97	0.10978981	2	2	4	0	0
97	0.10978981	3	0	7	2	0
97	0.10978981	2	0	7	0	0
97	0.10978981	2	0	7	1	0
97	0.10978981	2	0	5	2	0
97	0.10978981	2	0	11	1	0
97	0.10978981	2	0	2	0	0
97	0.10978981	2	0	0	7	0
97	0.10978981	2	0	10	0	0

138	0.00681655	3	0	9	0	0
139	-0.4657482	2	0	0	4	0
139	-0.4657482	4	0	0	3	4
139	-0.4657482	4	0	0	0	0
139	-0.4657482	1	0	0	0	7
139	-0.4657482	1	2	0	0	4
139	-0.4657482	1	4	0	0	4
139	-0.4657482	1	0	0	0	6
139	-0.4657482	1	0	0	0	8
139	-0.4657482	1	0	0	7	0
139	-0.4657482	1	0	0	8	0
139	-0.4657482	1	0	0	2	4
139	-0.4657482	1	0	0	1	3
139	-0.4657482	1	0	0	0	0
139	-0.4657482	4	2	0	0	5
139	-0.4657482	1	6	0	0	0
139	-0.4657482	1	0	0	0	5
139	-0.4657482	1	3	0	3	4
139	-0.4657482	2	0	0	5	0
139	-0.4657482	1	5	0	0	0
139	-0.4657482	1	7	0	0	0
139	-0.4657482	1	4	0	3	0
139	-0.4657482	2	2	0	0	0
139	-0.4657482	1	3	0	3	0
139	-0.4657482	2	0	0	7	0
139	-0.4657482	1	3	3	0	0
139	-0.4657482	1	0	0	7	0
139	-0.4657482	2	7	0	0	0
139	-0.4657482	4	0	0	6	0
139	-0.4657482	2	0	0	4	0
139	-0.4657482	1	8	0	0	0
139	-0.4657482	1	2	2	0	0
96	-0.8769027	4	0	0	14	0
96	-0.8769027	4	0	0	3	2
96	-0.8769027	4	0	0	4	2
96	-0.8769027	4	0	0	0	8
96	-0.8769027	4	0	0	0	8
96	-0.8769027	4	0	0	0	6
96	-0.8769027	4	2	0	0	6
96	-0.8769027	4	0	0	0	0
96	-0.8769027	4	0	0	3	3
96	-0.8769027	4	0	0	0	8
96	-0.8769027	4	0	0	0	8
96	-0.8769027	3	3	0	2	0
96	-0.8769027	3	6	0	1	0
96	-0.8769027	3	0	2	2	1
96	-0.8769027	3	0	2	5	0
96	-0.8769027	3	0	1	8	0
96	-0.8769027	3	0	0	10	0
96	-0.8769027	4	0	0	9	0
96	-0.8769027	2	11	0	0	0
44	-0.8338155	1	2	0	4	0
44	-0.8338155	4	0	13	0	0
44	-0.8338155	4	0	0	0	11
44	-0.8338155	4	0	0	0	4
44	-0.8338155	1	3	0	0	0
44	-0.8338155	1	8	0	0	0
44	-0.8338155	1	1	0	4	0
44	-0.8338155	2	4	0	0	0
44	-0.8338155	1	0	0	7	0
44	-0.8338155	3	0	2	3	1
44	-0.8338155	3	0	1	5	0
44	-0.8338155	3	5	2	0	0
44	-0.8338155	3	4	0	4	0
44	-0.8338155	2	0	7	0	0
44	-0.8338155	4	7	0	0	0
44	-0.8338155	1	6	0	4	0
44	-0.8338155	1	3	0	0	0
46	-0.5263299	4	4	2	0	0
46	-0.5263299	4	0	0	4	6
46	-0.5263299	4	1	0	4	2
46	-0.5263299	4	0	0	0	9
46	-0.5263299	4	0	0	0	8
46	-0.5263299	4	0	0	7	0
46	-0.5263299	1	0	0	11	0
46	-0.5263299	1	6	1	0	0
46	-0.5263299	1	0	0	7	0
46	-0.5263299	1	10	0	0	0
46	-0.5263299	1	2	0	4	0
46	-0.5263299	1	2	4	0	0
46	-0.5263299	1	6	0	0	0
46	-0.5263299	1	4	4	0	0
140	0.11305079	1	1	2	3	1
140	0.11305079	1	2	0	2	3
140	0.11305079	4	0	0	0	6
140	0.11305079	1	7	0	2	0
140	0.11305079	4	0	2	2	0
140	0.11305079	3	9	3	0	0
140	0.11305079	2	0	5	0	0
140	0.11305079	3	3	0	0	4
140	0.11305079	1	8	0	0	0
140	0.11305079	1	1	6	0	0
35	0.08994369	4	0	0	3	2
35	0.08994369	4	0	0	7	2
35	0.08994369	4	0	0	0	0
35	0.08994369	4	0	0	1	2
35	0.08994369	4	0	0	0	7
35	0.08994369	4	0	0	2	4
35	0.08994369	4	0	0	0	0
35	0.08994369	4	0	0	0	0
35	0.08994369	4	0	0	4	2
35	0.08994369	4	0	0	4	5
35	0.08994369	4	3	3	0	0
35	0.08994369	1	0	0	4	0
35	0.08994369	1	0	0	5	0
35	0.08994369	1	0	0	5	0
35	0.08994369	1	4	0	3	0
35	0.08994369	1	4	1	2	0
35	0.08994369	1	2	0	3	0
35	0.08994369	1	4	0	2	0
35	0.08994369	1	2	3	0	0
35	0.08994369	1	11	0	0	0
141	0.0553104	4	0	1	0	2
141	0.0553104	4	0	2	0	4
141	0.0553104	4	2	0	0	2
141	0.0553104	4	0	0	0	5
141	0.0553104	4	5	0	0	2
141	0.0553104	4	0	0	0	6
141	0.0553104	4	0	0	0	6
141	0.0553104	4	0	0	0	4
141	0.0553104	1	3	0	7	0
141	0.0553104	3	0	0	9	0
141	0.0553104	3	0	1	5	1
141	0.0553104	3	0	3	5	0
141	0.0553104	3	2	0	5	0
141	0.0553104	3	0	0	4	0
141	0.0553104	3	0	0	0	0
141	0.0553104	3	0	0	5	0
141	0.0553104	3	2	4	0	0
141	0.0553104	1	0	3	0	0

12	0.03251334	1	0	0	0	0
12	0.03251334	1	0	0	0	0
12	0.03251334	1	6	0	0	0
12	0.03251334	1	7	0	0	0
12	0.03251334	1	10	0	0	0
12	0.03251334	1	0	0	0	0
57	0.46721311	1	8	0	0	0
57	0.46721311	1	6	0	0	0
57	0.46721311	1	4	0	0	0
57	0.46721311	1	13	0	0	0
57	0.46721311	1	0	0	0	0
57	0.46721311	1	8	0	0	0
57	0.46721311	1	0	0	0	5
57	0.46721311	1	0	0	0	6
57	0.46721311	1	11	0	0	0
57	0.46721311	1	6	0	0	1
57	0.46721311	1	4	0	0	4
57	0.46721311	4	0	0	0	12
57	0.46721311	1	4	0	0	1
57	0.46721311	1	6	0	0	0

97	0.10978981	3	0	2	7	0
97	0.10978981	3	0	3	2	0
97	0.10978981	3	0	1	3	0
97	0.10978981	3	0	7	2	0
97	0.10978981	4	0	7	0	0
97	0.10978981	2	0	5	2	0
97	0.10978981	2	0	4	3	0
97	0.10978981	3	0	2	5	0
97	0.10978981	3	0	7	0	0
97	0.10978981	3	0	0	6	0
97	0.10978981	4	0	0	0	5
98	0.41604249	2	0	6	0	0
98	0.41604249	2	0	9	0	0
98	0.41604249	3	0	9	0	0
98	0.41604249	2	0	10	0	0
98	0.41604249	2	0	5	1	0
98	0.41604249	3	0	7	1	0
98	0.41604249	2	0	0	0	0
98	0.41604249	2	0	1	6	0
98	0.41604249	3	0	3	3	0

141	0.0553104	2	2	7	0	0
142	-0.4354791	4	0	0	0	10
142	-0.4354791	4	0	0	0	7
142	-0.4354791	4	0	0	4	1
142	-0.4354791	3	2	0	3	0
142	-0.4354791	1	5	0	2	0
142	-0.4354791	3	0	1	5	0
142	-0.4354791	3	2	1	2	0
142	-0.4354791	3	4	0	4	0
142	-0.4354791	3	0	6	0	0
38	-0.6944448	4	0	0	3	4
38	-0.6944448	4	0	0	1	5
38	-0.6944448	4	0	0	0	8
38	-0.6944448	4	0	0	7	0
38	-0.6944448	3	5	0	4	0
38	-0.6944448	2	0	0	10	0
38	-0.6944448	2	3	1	4	0
38	-0.6944448	3	1	0	7	0
143	0.31324494	1	8	0	0	0
143	0.31324494	1	0	0	8	0
143	0.31324494	1	0	3	5	0
143	0.31324494	1	3	3	0	0
143	0.31324494	1	0	0	0	0
143	0.31324494	1	8	0	0	0
143	0.31324494	1	4	2	1	0
143	0.31324494	2	5	2	0	0
